# Machine learning for predicting neurodegenerative diseases in the general older population: a cohort study

**DOI:** 10.1186/s12874-023-01837-4

**Published:** 2023-01-11

**Authors:** Gloria A. Aguayo, Lu Zhang, Michel Vaillant, Moses Ngari, Magali Perquin, Valerie Moran, Laetitia Huiart, Rejko Krüger, Francisco Azuaje, Cyril Ferdynus, Guy Fagherazzi

**Affiliations:** 1 Deep Digital Phenotyping Research Unit, Department of Precision Health, Luxembourg Institute of Health, Strassen, Luxembourg; 2 Bioinformatics Platform, Luxembourg Institute of Health, Strassen, Luxembourg; 3 Competence Center for Methodology and Statistics, Translational Medicine Operations Hub, Luxembourg Institute of Health, Strassen, Luxembourg; 4 KEMRI/Wellcome Trust Research Programme, Kilifi, Kenya; 5 Department of Precision Health, Luxembourg Institute of Health, Strassen, Luxembourg; 6 Living Conditions Department, Luxembourg Institute of Socio-Economic Research, Esch-Sur-Alzette, Luxembourg; 7 LCSB, Luxembourg Centre for System Biomedicine, University of Luxembourg, Esch-Sur-Alzette, Luxembourg; 8 Parkinson Research Clinic, Centre Hospitalier de Luxembourg, Luxembourg, Luxembourg; 9 Transversal Translational Medicine, Luxembourg Institute of Health, Strassen, Luxembourg; 10 Genomics England, London, UK; 11 Methodological Support Unit, Félix Guyon University Hospital Center, Saint-Denis, La Réunion France

**Keywords:** Deep neural networks, Cox models, Parkinson disease, Alzheimer, Dementia, Prediction, Tabular data, Older general population

## Abstract

**Background:**

In the older general population, neurodegenerative diseases (NDs) are associated with increased disability, decreased physical and cognitive function. Detecting risk factors can help implement prevention measures. Using deep neural networks (DNNs), a machine-learning algorithm could be an alternative to Cox regression in tabular datasets with many predictive features. We aimed to compare the performance of different types of DNNs with regularized Cox proportional hazards models to predict NDs in the older general population.

**Methods:**

We performed a longitudinal analysis with participants of the English Longitudinal Study of Ageing. We included men and women with no NDs at baseline, aged 60 years and older, assessed every 2 years from 2004 to 2005 (wave2) to 2016–2017 (wave 8). The features were a set of 91 epidemiological and clinical baseline variables. The outcome was new events of Parkinson’s, Alzheimer or dementia. After applying multiple imputations, we trained three DNN algorithms: Feedforward, TabTransformer, and Dense Convolutional (Densenet). In addition, we trained two algorithms based on Cox models: Elastic Net regularization (CoxEn) and selected features (CoxSf).

**Results:**

5433 participants were included in wave 2. During follow-up, 12.7% participants developed NDs. Although the five models predicted NDs events, the discriminative ability was superior using TabTransformer (Uno’s C-statistic (coefficient (95% confidence intervals)) 0.757 (0.702, 0.805). TabTransformer showed superior time-dependent balanced accuracy (0.834 (0.779, 0.889)) and specificity (0.855 (0.0.773, 0.909)) than the other models. With the CoxSf (hazard ratio (95% confidence intervals)), age (10.0 (6.9, 14.7)), poor hearing (1.3 (1.1, 1.5)) and weight loss 1.3 (1.1, 1.6)) were associated with a higher DNN risk. In contrast, executive function (0.3 (0.2, 0.6)), memory (0, 0, 0.1)), increased gait speed (0.2, (0.1, 0.4)), vigorous physical activity (0.7, 0.6, 0.9)) and higher BMI (0.4 (0.2, 0.8)) were associated with a lower DNN risk.

**Conclusion:**

TabTransformer is promising for prediction of NDs with heterogeneous tabular datasets with numerous features. Moreover, it can handle censored data. However, Cox models perform well and are easier to interpret than DNNs. Therefore, they are still a good choice for NDs.

**Supplementary Information:**

The online version contains supplementary material available at 10.1186/s12874-023-01837-4.

## Background

Neurodegenerative diseases (NDs) are a leading cause of disability in the older population [1]. Alzheimer’s disease (AD) and Parkinson’s disease (PD) are the two most common NDs, and their prevalence increases with increasing age [2]. NDs have long prodromal periods that can manifest many years before the onset of the respective disease [3, 4]. Parkinson’s disease, Alzheimer and other types of dementia are diseases that have heterogeneity in their clinical presentation, physiological mechanisms and some predictors. However, recent evidence has shown that these diseases may share some relevant aspects, such as genetic susceptibility, underlying mechanisms and other predictors [5, 6]. Besides rare monogenic forms of these diseases, most cases with NDs are due to an interplay of genetic susceptibility factors and some environmental risk factors [7, 8]. Identifying these risk factors is crucial for early intervention and can help delay disease onset.

It exists already research on the prediction of neurodegenerative diseases. For example, in a large cohort study, researchers have reported results on the prediction of NDs using traditional statistical analyses (hypothesis-driven approaches) [9]. Another cohort study assessed 14,066 older participants free of cognitive decline with a follow-up of 4.5 years. Using Cox models, they found that subjective cognitive decline and anxiety were independently associated with mild cognitive impairment and dementia [10]. Cohort studies based on samples from the general population can providing information with less selection and recall bias than case-control studies [4]. Reinke et al. studied dementia risk in a population with German claims data in 117,895 individuals during a 10-year follow-up. They performed three different ML algorithms obtaining moderated discriminate accuracy from 0.64 (random forests) to 0.7 (logistic regression and gradient boosting) [11]. However, prediction models of NDs in cohort studies (designed to answer specific questions and have subjective and objective for-purposed information) with participants from the general population are not frequently performed due to the difficulty of obtaining funding, having an adequate sample size and an extended follow-up.

In the last years, researchers started to use data-driven approaches with machine learning (ML) for NDs prediction [12]. A potential advantage of ML models over traditional statistical analysis in prediction is the ability to handle high dimensional data [13]. Despite this evidence, it is unclear whether ML algorithms would have a superior discriminative ability in predicting NDs in cohort studies compared to traditional statistical methods. Among ML algorithms, deep neural networks (DNNs) have advantages over other methods. DNNs are more flexible and able to include images and any input data. In addition, they can easily handle missing data, model non-linear and complex relationships [14]. DNNs also can handle survival time if the DNN algorithm is tailored to censored data by with the appropriate censoring unbiased loss functions [15–17]. The disadvantages are that most DNNs do not perform appropriately with heterogeneous tabular data [18]. Researchers have recently developed algorithms with different structures that can deal with tabular-heterogeneous data to fill this gap [18] Still, these algorithms have not been widely investigated yet to time-to-event outcomes.

This study aims to test different algorithms for NDs prediction in the older general population using Cox models with a selection of variables and deep learning techniques. Another objective is to discover predictors for NDs that can be informative for public health prevention of these diseases.

We hypothesized that DNNs fitted for tabular data would perform better than other neural networks in predicting neurodegenerative diseases and perform as well as regularized Cox models.

## Methodology

### Participants, inclusion criteria and study design

We analysed participants of the English Longitudinal Study of Ageing (ELSA) [19], an ongoing cohort study representative of the general population over 50 years of age living in England. ELSA collects health data, including socio-economic, cognitive, behavioural, psychological, and lifestyle information. Participants are assessed every 2 years (waves) with computer-assisted interviews and self-reported questionnaires. Each biennial assessment is called a “wave”. In addition, every 4 years, participants undertake a physical exam and provide blood samples. The data collection goes from wave 1 (baseline for the ELSA study, performed in 2002–2003) to wave 9 (2018/− 2019). Ethical approval was obtained from the Multicentre Research and Ethics Committee [20].

### Eligibility criteria

We included participants 60 years and older at wave 2 (2004–2005) because some crucial variables were measured from wave 2 (nurse visit) and not in people younger than 60. We excluded all participants that, at wave 2 (the baseline of this study), had a diagnosis of NDs (PD, AD or dementia) or had a score < = 1 in questions about the date from the Mini-Mental Status Examination score. At the moment of the analysis, the last available assessment was wave 8. Consequently, we followed up on the participants’ outcomes from wave 3 to wave 8.

### Study design

This study is an observational retrospective longitudinal secondary analysis of ELSA and no formal written analysis plan exists. We analysed a period of 12 years of follow-up from 2004 to 2005 (wave 2, baseline of this analysis) to 2016–2017 (wave 8).

### Outcome

The outcome was any new event of NDs during the follow-up. The composite variable “NDs” was defined as ever reported PD or AD, dementia or high memory impairment. The question was as follows: “Has a doctor ever told you that you [have/have had] any of the conditions in this card? (PD, AD, dementia or high memory impairment)”. Dementia was additionally defined with the questions about the date of the Mini-Mental Status Examination score 16 less or equal to 1 (0 worst, 4 = best).

### Features

Based on the literature [7, 8], we chose possible predictors (features) of NDs that were available in the ELSA Wave 2 dataset. We selected 95 baseline variables (features) associated with the occurrence of NDs or expected outcomes. We identified 27 comorbidities, 15 psychosocial, 11 biomarkers, 9 symptoms, 7 lifestyle, seven environmental, 6 physical functioning tests, 6 disability, 4 cognition tests and three demographic variables (Supplementary Table 1). Eleven of the 13 risk factors for dementia reported by the Lancet commission for dementia prevention [21] are among the 91 features. The two not included features (traumatic brain injury and air pollution) are unavailable in wave 2 of the ELSA study. Among the 95 selected features, four variables with variance inflation factor (VIF) > 10 were excluded from the analysis. We analysed and selected 91 features in our five final models (input of the models).

### Statistical analysis

#### Missing data

We checked missingness in every variable of interest. Assuming a missing at-random mechanism, a complete-case analysis would introduce bias [22]. Consequently, we applied multiple imputations to deal with the missing data issue. We imputed only the baseline predictor variables and not the outcome. We built the imputation model with the full dataset by selecting the best missing data predictors. The function “Quickpred” from the “mice” R package allows a selection of predictors according to correlations and usable cases. We selected the best predictors among the available variables with the function “Quickpred” and included the outcome and possible confounders such as age and sex [23]. To decide the number of imputations, we used the maximum percentage of observed missing data [24]. Then, we checked the imputations by comparing imputation with non-imputation means and calculating the percentage of bias. A value of 5% or less is considered acceptable [25].

#### Data pre-processing

Categorical predictors were dichotomized into 0, and 1. To deal with numerous continuous predictors with a skewed distribution, we transformed them with logarithms to the base 2 + 1 with the following formula: y = log_2_ (x) + 1. y = transformed predictor; x = original non-transformed predictor. We used logarithms to the base 2 because of its binary nature, which makes the computation of machine learning more performant.

Nested cross-validation was carried out to reduce the risk of model overfitting. In the nested cross-validation, we used two repeated 5-fold cross-validation in each of the datasets obtained from the multiple imputation stage to have ten datasets to train (80% of data) the model and ten datasets to test (20% of data) the model. Then, we performed feature selection and hyper-parameter tuning only on the training datasets. We normalized the training and test data using the minimum and maximum values for each variable computed from training data during the analysis.

The time at risk was defined from the baseline (wave 2) in 2004/2005 to the follow up (wave 8) in 2016/2017. We tested the proportional hazard assumption by using Schoenfeld residuals. Using VIF” (“rms” R package), we sequentially removed the variables with high multi-collinearity (VIF > 10). The set of baseline variables that were not removed in this process was modelled as predictors in Cox models and was the input of the DNNs models (Supplementary Table 1).

After the standard processes of selecting variables, multiple imputations and pre-processing, the analytical approaches are presented separately.

#### Cox models

We generated two different Cox models.

#### Cox models with elastic net regularization (CoxEn)

Regularisation is a machine learning technique that penalises coefficients that deviates from zero. It may help avoid overfitting and increase computation performance and interpretability of the results [26]. Lasso (L1 regularisation that restricts the size of the coefficients) and Ridge (L2 regularisation that restricts the square of the magnitude of the coefficients) regressions are two well-known regularisation techniques [27]. Elastic Net is a technique which combines both Lasso and Ridge techniques for better performance [28]. Using the pre-selected features as predictors, we performed Elastic Net regularization. The process that we used has two tuning parameters: the regularization parameter lambda and the mixing parameter alpha for moderating between Lasso and Ridge [29]. The optimal model should specify alpha and lambda, for which the two repeated 5-fold cross-validated penalized log-likelihood deviance is minimal after comparing all the training datasets from 40 imputed datasets. The “c060” and “glmnet” R packages provided parameter tuning and C-index computing functions.

#### Cox models with selected features (CoxSf)

We applied Elastic Net regularisation in 10 randomly chosen training datasets from each of the 40-imputed datasets. We kept the features selected (i.e. having coefficients not equal to 0) at least eight times from the 10 training datasets of each imputed dataset. We selected the variables according to how often each variable appeared in the imputed datasets. We computed the number of imputed datasets that a variable appeared. We wanted to use the variables that occurred in at least 30 imputed datasets in a Cox model. For example, a variable “x” appeared in all 40 imputed datasets. In consequence, it was kept for the Cox model. In contrast, a variable “y”, which occurred only in 24 imputed datasets, was excluded from the model. Using the variables that occurred in 30 to 40 imputed datasets, we applied Cox regression models to each imputed dataset and pooled them with Rubin’s rules (pool function from the “mice” R package) [23]. The pooled models with different variables were compared using the Wald test (pool.compare function from the “mice” R package). We kept the variables in the model with the lowest *p*-value compared to the other models.

#### Deep neural networks

**Fig. 1:**
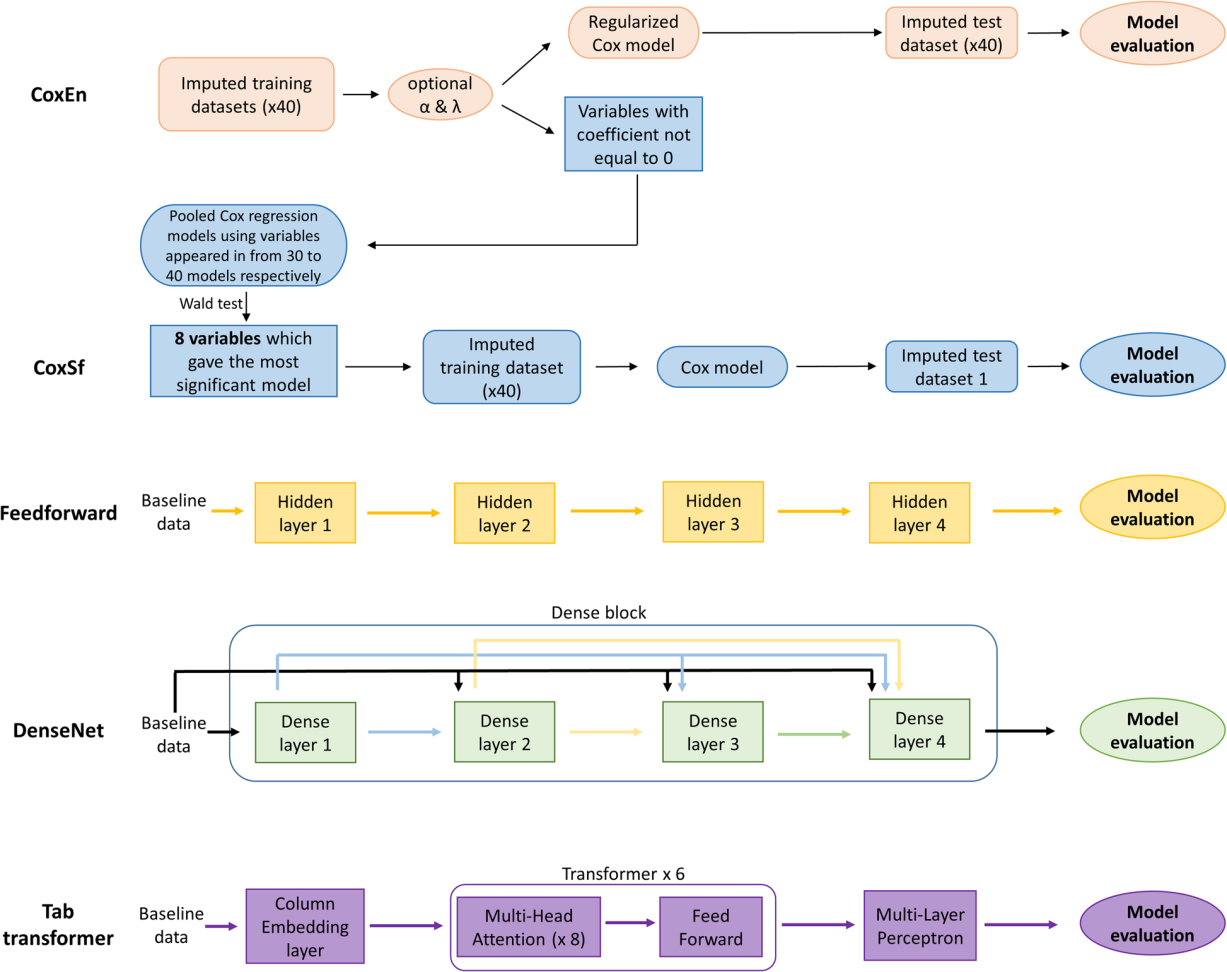
Model architecture developed for prediction of new events of neurodegenerative diseases. Time of follow-up: from 2004 to 2005 to 2016–2017. Population: The English Longitudinal Study of Ageing. Cox models with Elastic Net regularisation are in salmon, and Cox models with selected variables are in blue. The FeedForward model is in yellow, the Densenet model is in green and the TabTransformer model is in blue-violet. In the deep neural network models (Feedforward, Densenet and TabTransformer), the input was the baseline data (91 features) and the log-risk function is the output of the network

TensorFlow API 2.3.0 [30] allowed the development and training of the three DNN models (Feedforward neural network, Densely Connected Convolutional Network and TabTransformer neural network). We used a loss based on the negative log of Breslow approximation partial likelihood that allows accounting for censored data. The data architecture of the five models (Cox models with Elastic Net regularization, Cox models with selected features, Feedforward neural network, Densely Connected Convolutional Network and TabTransformer neural network) is shown in Fig. 1.

#### Feedforward neural network (FeedForward)

Feedforward is a DNN where the information moves in only one direction, from the input layer, through the hidden layers and to the output layer [31]. We included one input layer (the pre-selected variables), four fully connected hidden layers and one output layer. Each hidden layer had 32 neurons, followed by a dropout layer with a dropout rate = 0.2 and a Gaussian Noise layer which is used to mitigate overfitting. The output was a single node with a linear activation that estimates the log-risk function in the Cox model. We used Scaled Exponential Linear Units (SELU) as the activation function and Adaptive Moment Estimation (Adam) with a learning rate = 0.0001 for the gradient descent algorithm.

#### Densely connected convolutional network (DenseNet)

DenseNet is a DNN and consists of a series of pre-connected layers (dense layers) connected to the previous or next layer. Information from all previous layers is used as input for each layer, and therefore all the information is propagated through the whole model to limit gradient vanishing [32]. We used a 4-layer dense block. The input and the hyper-parameters of the dense layer were the same as those for FeedForward. Each dense layer outputs 8 features (growth rate), which were used as the input of the next layers.

#### TabTransformer neural network (TabTransformer)

The TabTransformer is a deep tabular data modelling neural network. It uses contextual embedding, and it is based on the self-attention mechanism [33]. We chose an embedding size of 64 neurons followed by a stack of six Transformer Layers with eight heads each. The inputs of TabTransformer were the same that for all models. We modified TabTransformer to use a Cox layer as the output layer and a censoring unbiased loss function based on the negative log of Breslow approximation partial likelihood that allows accounting for censored data [16, 34]. Gradient descent optimization with adaptive moment estimation was performed with a learning rate of 0.0001.

#### Model evaluation

The output of the three DNNs is the predicted values of new events of NDs for each participant. Then we used these values to calculate the assessment measures.

C-statistics (or C-index) measures a model’s goodness of fit, giving the probability that an individual that experienced the event has a higher score than an individual that did not experience the event. Harrell’s C- statistics is a type of c-statistics with a rank correlation method for censored data. Uno’s C-statistics has an advantage over Harrell’s C- statistics as it does not depend on the study-specific censoring distribution [35]. We evaluated the performance of the models in the test datasets by measuring Uno’s C-statistics with 95% confidence intervals calculated with 100 replications of bootstrapping. We assessed the following time-dependent measures: AUC, balanced accuracy, sensitivity and specificity.

We assessed the overfitting of DNNs by plotting the loss function over epochs in each of the imputed training and test datasets. The stability of the models was assessed by calculating confidence intervals with bootstrapping with the Uno’s C-statistic in each of the 40 imputed datasets. Finally, we calculated the power of our sample size for the categorical CoxSf model final predictors.

We assessed the feature importance of the three DNNs using Shapley additive explanations (SHAP) analysis, showing the top ten most important features for each model [36]. We analysed the possible shared features among the three DNNs and the Cox model using visual methods.

Data analyses were performed in R version 4.0.0 using R packages “mice”, “survival”, “glmnet”, “c060” and, “survivalROC”. Sample split and Uno C-statistics calculations were performed with Python.

This study is reported as per the Transparent reporting of a multivariable prediction model for individual prognosis or diagnosis (TRIPOD).

## Results

From 9432 participants at baseline, we excluded 3999 younger than 60 years or had a NDs diagnosis. We included 5433 participants (Supplementary Fig. 1) who experienced 691 (13%) NDs events during the 12-year follow-up. The median follow-up was 10 (interquartile range (IQR) 1) years. Participants with NDs at baseline were older, less frequently married, less qualified, more frequently sedentary, with slower walking speed, with a higher frequency of falls, more regularly affected by cardiovascular disease and had lower cognitive scores. (Table 1).

**Table 1:** Description of 5433 participants in the English Longitudinal Study of Ageing at wave 2 (baseline 2004–2005) stratified by the apparition of events during the follow-up

Characteristic	All sample (***n*** = 5433)	No NDs events (***n*** = 4742)	NDs events (***n*** = 691)
Age, median (IQR), years	70 (12)	69 (10)	75 (13)
Sex, No. (%)			
Male No. (%)	2408 (44)	2123 (45)	285 (41)
Married or cohabiting (%)^a^	3339 (61)	2948 (62)	391 (57)
Education non-qualified ^a, b^	2928 (54)	2511 (53)	417 (60)
Former smoker (%)^a^	2773 (51)	2422 (51)	351 (51)
Current smoker (%)^a^	691 (13)	606 (13)	85 (12)
Sleep disturbance, No. (%)^a^	2274 (42)	1971 (42)	303 (44)
BMI, mean (SD), kg/m^2 a^	28 (5)	28 (5)	28 (5)
Obesity, No. (%) ^a, c^	1546 (28)	1361 (29)	185 (27)
Weight loss (%)	956 (18%)	809 (17)	147 (21)
Physical activity, No. (%)^a^			
Low level or inactive	1941 (36)	1626 (34)	315 (46)
Moderate or vigorous	3492 (64)	3116 (66)	376 (54)
Gait speed, mean (SD), m/s ^a^	0.84 (0.30)	0.85 (0.30)	0.74 (0.30)
Falls, No. (%)^a^	1789 (33)	1519 (32)	270 (39)
Systolic blood pressure, mean (SD), mmHg^a^	138 (31)	138 (33)	139 (32)
Diastolic blood pressure, mean (SD), mmHg^a^	74 (15)	74 (15)	73 (16)
Cardiovascular disease, No. (%)^d^	750 (14)	635 (13)	115 (17)
Stroke, No. (%)	344 (6)	289 (6)	55 (8)
Diabetes, No. (%) ^a, e^	512 (9)	442 (9)	70 (10)
Hypertension, No. (%)^e^	2646 (49)	2294 (48)	352 (51)
Depression, No. (%)^a, f^	1765 (32)	1489 (31)	276 (40)
Cognition (executive), mean (SD), pp^a^	12 (3)	12 (3)	11 (3)
Cognition (memory), mean (SD), pp^a^	15 (4)	15 (4)	12 (4)
Psychiatric diagnosis, No. (%)^e^	425 (8)	369 (8)	56 (8)

^a^Imputed data and if SD, it was calculated according to Rubin’s rules

^b^No graduation certificate (primary or secondary studies)

^c^Defined as BMI > = 30 kg/m2

^d^Defined as self-reported infarction, stroke or heart failure

^e^Self-reported medical diagnosis

^f^Defined as a score > =4 of the 8-item Center for Epidemiological Studies-Depression (CES-D). *Abbreviations*: *NDs* Neurodegenerative diseases, *IQR* Interquartile range, *SD* standard deviation, *BMI* Body mass index, *pp*. Per point

Missing data were observed in 1 (< 1.0%) to 1818 (33.5%) participants (Supplementary Table 1). In the imputation model, we included age, sex, time-to-event and outcomes. We created 40 imputed datasets with 20 iterations using chained equations [24]. The imputations were values considered as plausible and with a low percentage of bias (Supplementary Table 2). We also observed a distribution of imputed data similar to the original non-imputed values (Supplementary Figs. 2 to 4).

All models had as initial input the 91 pre-selected variables described in the methods section. After having verified the proportional hazard assumption was not violated, we generated the CoxEn models with Elastic Net regularization. We used alpha = 0.84 and lambda = 0.0093, which gave the lowest partial likelihood deviance.

**Fig. 2:**
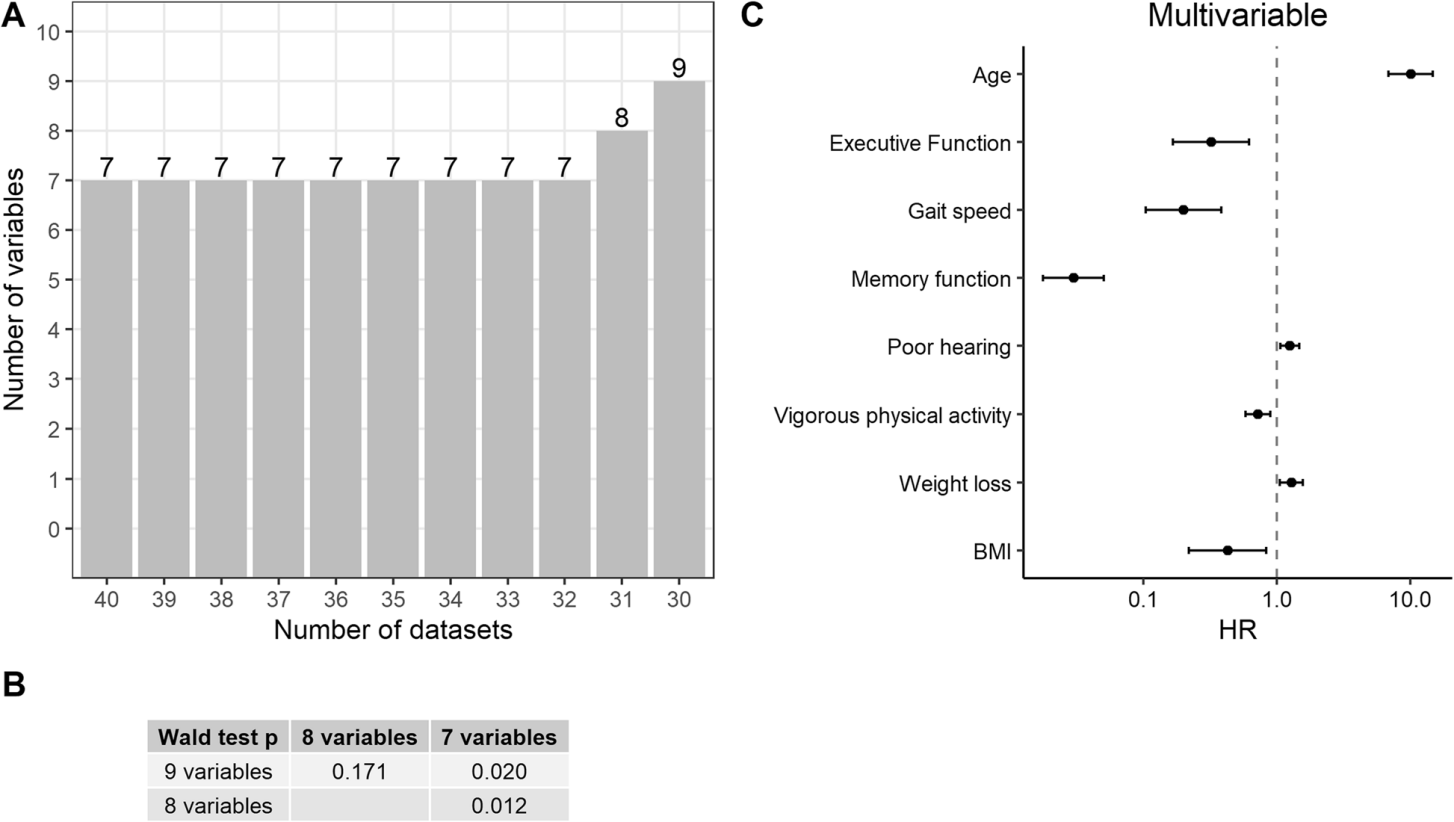
Cox model with selected features and pooled Cox regression model of new events of neurodegenerative diseases. Panel **A**: The number above the columns shows the number of features appearing in different numbers of imputed datasets (from 30 to 40). Seven variables appeared in 32 to 40 imputed datasets, 8 variables in 31 datasets and 9 variables in 30 datasets. Panel **B**: *P* values from the Wald test on the pooled Cox regression models with different numbers of variables. *P* values < 0.05 are shown in bold. The model with eight variables showed the most significant difference (smallest *p* value) compared to the other models. Panel **C**: Hazard ratios and 95% confidence intervals in Cox regression model with eight selected variables (CoxSf) pooled according to the Rubin’s rules. All the selected variables were significantly associated with neurocognitive disorders

To find the best list of features for the final CoxSf, we generated models on 40 imputed datasets using 7, 8 and 9 selected variables, respectively and pooled them by following Rubin’s rules. Nine variables appeared in at least 30 imputed datasets, eight in at least 31 imputed datasets and seven in all 40 imputed datasets (Fig. 2 Panel A). Using the Wald test, the model with eight variables had the highest significant difference compared to the other models (Fig. 2 Panel B). The final model CoxSf included variables associated with higher risk: older age (hazard ratio (95% confidence intervals)) (10.0 (6.9, 14.7)), poor hearing (1.3 (1.1, 1.5)) and weight loss (1.3 (1.1, 1.6)). Executive function (0.3 (0.2, 0.6)), memory function (0.03, (0.02, 0.05)), increased gait speed (0.2, (0.1, 0.4)), vigorous physical activity (0.7, (0.6, 0.9)) and higher BMI (0.4 (0.2, 0.8)) were associated with a lower ND risk (Fig. 2 Panel C). The DNNs models were generated with the described methodology.

**Fig. 3:**
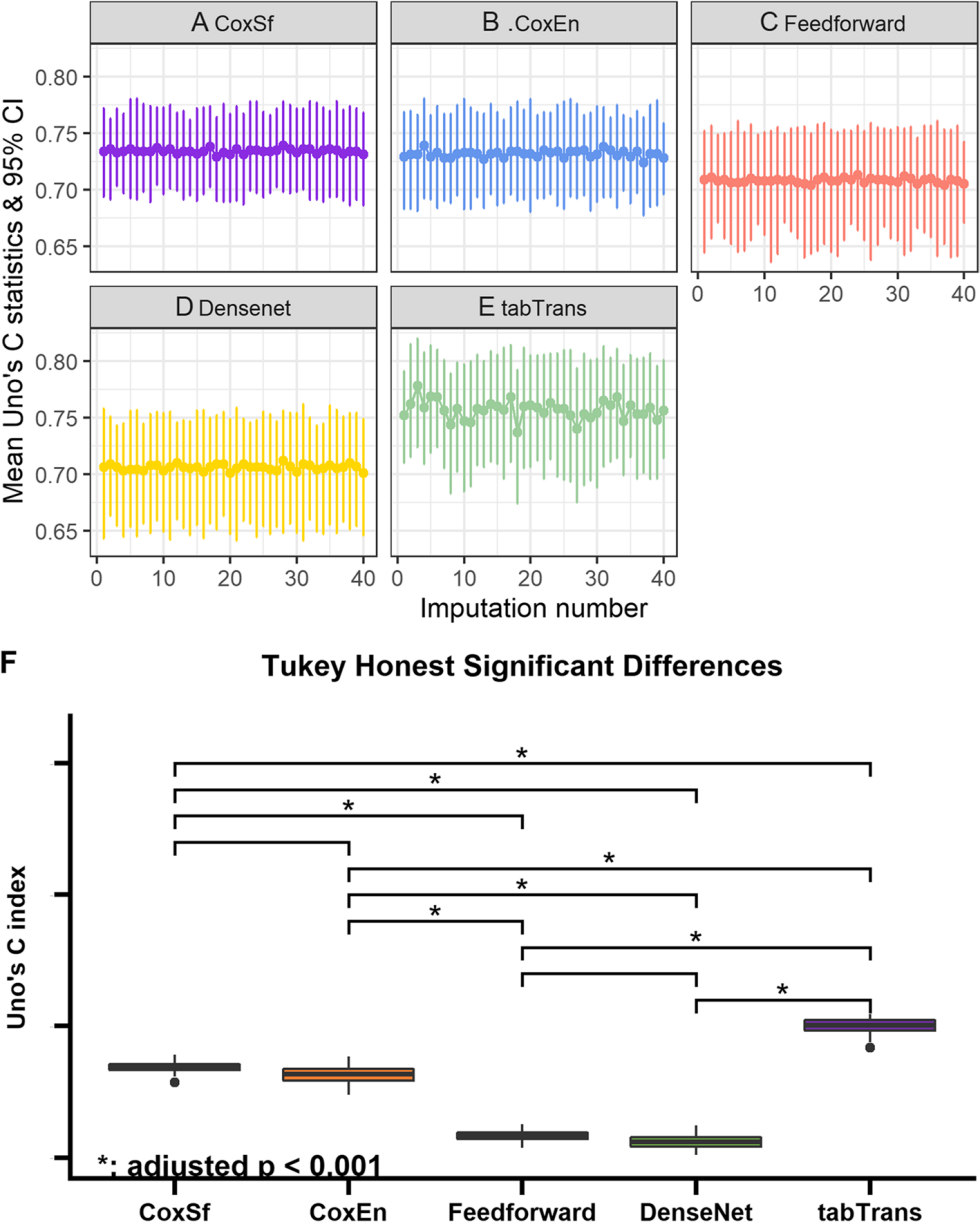
Assessing the models for predicting new events of neurodegenerative diseases from 2004 to 2005 to 2016–2017. The English Longitudinal Study of Ageing. Bootstrapping results of the mean (and 95% confidence intervals) of Uno’s C-statistics on the 40 imputed test datasets. Panel **A** shows Cox regression model with eight selected variables (CoxSf). Panel **B** shows Elastic Net regularised Cox regression model (CoxEn). Panel **C** shows FeedForward neural network model (Feedforward). Panel **D** shows DenseNet neural network model (Densenet). Panel **E** shows TabTransformer neural network (tabTrans). Panel **F** The difference of Uno’s C-statistic among the five models was significant (Tukey’s test adjusted *p* < 0.001)

The performance of the models from the highest to the lowest Uno’s C-statistic was (mean (95% confidence intervals) 0.757 (0.702, 0.805), 0.734 (0.694, 0.772), 0.732 (0.689, 0.771), 0.706 (0.651, 0.752), 0.708 (0.653, 0.754) for TabTransformer, CoxSf, CoxEn, Densenet and FeedForward respectively (Fig. 3 and Supplementary Table 3). Uno’s C-index from the TabTransformer model was significantly higher than the other models (Fig. 3, Tukey’s test adjusted *p* < 0.001). Uno’s C-index was not significantly different between CoxEn and CoxSf models (*p* = 0.07) and between Densenet and Feedforward (*p* = 0.13).

**Fig. 4:**
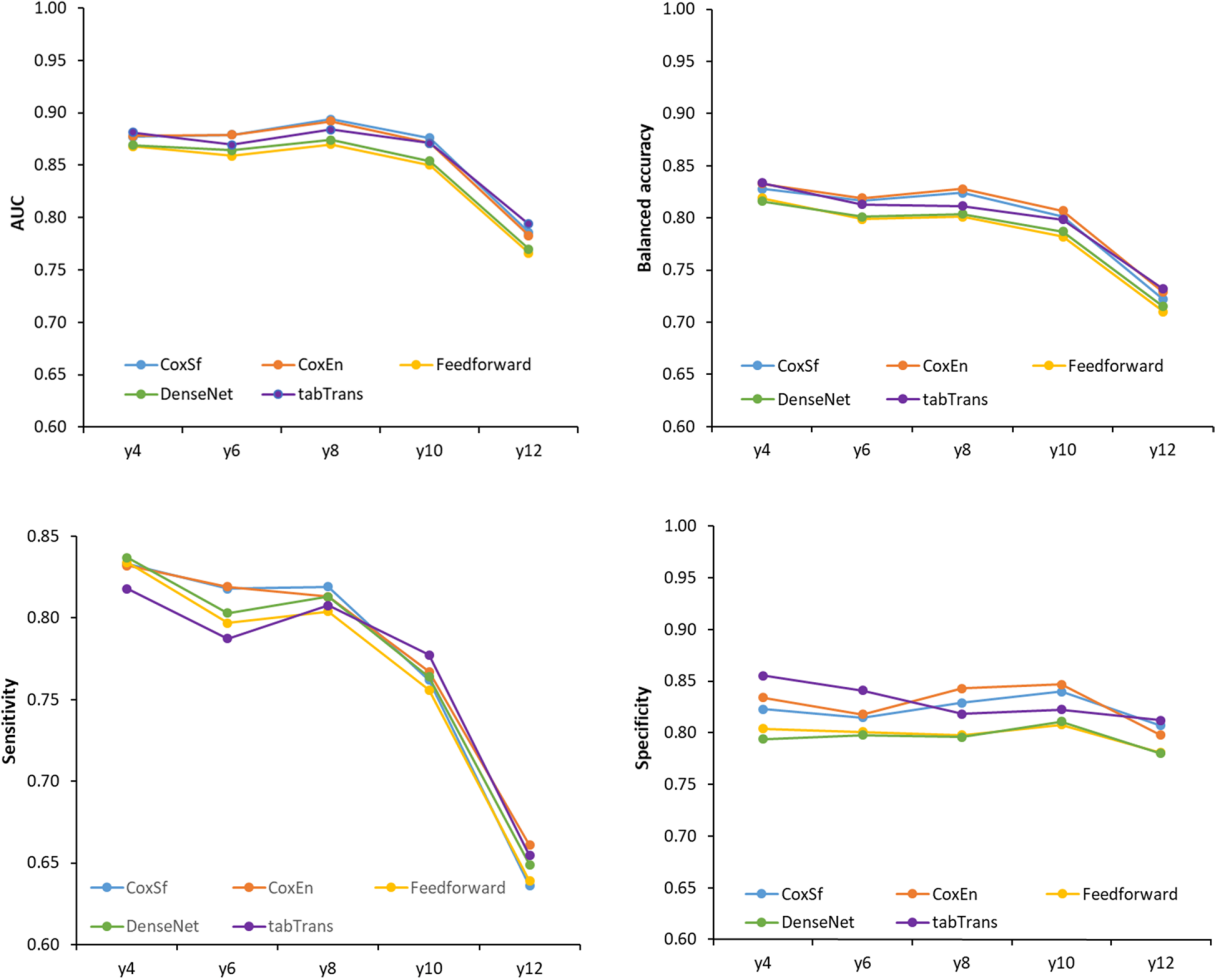
Time-dependent assessment of models predicting new events of neurodegenerative diseases. The curves represent the evolution of the performance assessed with time-dependent AUC, balanced accuracy, sensitivity and specificity for each of the five models. Panel **A**: The average of AUC from 40 imputed test datasets in 4, 6, 8, 10 and 12 years after the enrolment. Panel **B**: The average of balanced accuracy from 40 imputed test datasets in 4, 6, 8, 10 and 12 years after the enrolment. Panel **C**: The average of sensitivity from 40 imputed test datasets in 4, 6, 8, 10 and 12 years after the enrolment. Panel **D**: The average of specificity from 40 imputed test datasets in 4, 6, 8, 10 and 12 years after the enrolment

Figure 4 and Supplementary Tables 4 to 7 (in each imputed dataset) show the evolution of the time-dependent measures over time (time-dependent AUC, balanced-accuracy, sensitivity and specificity at 4, 8, 10 and 12 years of follow-up). TabTransformer shows better balance accuracy, specificity and much better sensitivity after 8 years than the other models.

Using time-dependent AUC the best model was the CoxSf in the 8th year of follow-up, All models showed the highest AUC in the 8th year of follow-up and were (mean (95% confidence intervals)) 0.894 (0.874, 0.913), 0.892 (0.872, 0.912), 0.870 (0.845, 0.894), 0.874 (0.849, 0.898), and 0.884 (0.863, 0.905) for CoxSf, CoxEn, Densenet, FeedForward and TabTransformer respectively (Supplementary Table 4 and Fig. 4). In the 12th year, all models showed a decrease in AUC values (mean decrease between 10 and 12th year: 10.1%).

The best-balanced accuracy values were observed in the 4th year. They were (mean (95% confidence intervals)) 0.833 (0.780, 0.888), 0.828 (0.775, 0.884), 0.819 (0.761, 0.877), 0.816 (0.762, 0.873) and 0.834 (0.779, 0.889) for CoxEn, CoxSf, FeedForward, Densenet and Tabtransformer respectively (Supplementary Table 5 and Fig. 4). In the 12th year, all models showed a decrease in balanced accuracy values (mean decrease between 10 and 12th year: 9.5%).

The highest sensitivity values were in the 4th year of follow-up. They were (mean (95% confidence intervals)) 0.832 (0.716 0.949), 0.833 (0.704, 0.957), 0.834 (0.688, 0.966), 0.837 (0.686, 0.965) and 0.818 (0.688, 0.936) for CoxSf, CoxEn, FeedForward, Densenet, and TabTransformer respectively (Supplementary Table 6 and Fig. 4). In the 12th year, all models decreased balanced accuracy values (mean decrease between 10 and 12th year: 16.1%).

The best specificity value was obtained in the 4th year by TabTransformer (0.855 (0.0.773, 0.909)). The highest specificity values were observed in the 10th year in the other models. They were (mean (95% confidence intervals)) 0.847 (0.754, 0.909), 0.840 (0.755, 0.903), 0.808 (0.716, 0.882), 0.811 (0.723, 0.883), 0.823 (0.754, 0.887), for CoxEn, CoxSf, FeedForward, Densenet and TabTransformer respectively (Supplementary Table 7 and Fig. 4).

We found that the TabTransformer showed a slightly wider separation between the validation and test curves compared with the Densenet and FeedForward models, which suggests that TabTransformer could experience more overfitting than the other models (Supplementary Fig. 5).

All models showed a stable variation of confidence intervals in the bootstrapping. However, TabTransformer tends to have slightly more irregular sizes of confidence intervals than the other models (Fig. 3).

The most critical features represented in the three DNNs were: age, memory function index, and vigorous physical activity (First row, Supplementary Fig. 6). Older age, lower values of memory function and a low reported vigorous physical activity were associated with a higher risk of NDs. Inversely, younger age, higher values of memory function and highly reported vigorous physical activity were associated with a lower risk of NDs. The highest impact for the models was older age and lower values of memory function (See second row, Supplementary Fig. 6). In addition, in Supplementary Fig. 7, we show which variables are present in more than one of all models (DNN and CoxSf). Age, memory function index and vigorous physical activity were present in all models. Poor hearing, gait speed and weight loss were present in three of four models. Chair rise outcome, executive function index, literacy score, measured hypertension and sleep quality were present in two of four models.

## Discussion

This study found that the TabTransformer compared to other DNN (Densenet and FeedForward) and regularized Cox models showed a superior discriminative ability to predict NDs events in an older general population. Due to the attention-based layers, TabTransformer performs well with heterogeneous data, particularly in managing categorical input, which is not the case with other neural networks [18]. In time-dependent assessment, TabTransformer, compared to the other models, performed similarly in AUC and balanced accuracy, slightly worse in sensitivity and better in specificity at the 4th and 6th years of follow-up. The prediction of NDs in the mid and long term is relevant because these conditions have long prodromal periods.

To our knowledge, this is the first time that Tabtransformer was used with censored data and multiple imputations for dealing with missing data for predicting an event in the general population.

We found that regularized CoxSf and CoxEn models performed better than FeedForward and Densenet. We used Elastic Net, an ML technique, for variable selection in our Cox models. Elastic Net improves the performance by choosing the most predictive variables, avoiding the issue of limiting the number of variables by the number of events. These findings agree with Spooner et al., which showed that variable selection with gradient boosting or Elastic Net improved the performance of Cox models [13].

A previous study has investigated prediction models of NDs. This study compared Cox models with a recurrent DNN to predict AD and found that the models with predictors as repeated measures performed better (C statistics =0.910) [37]. We observed a lower performance than that obtained by Kim et al., which may be due to different characteristics of the sample and because our outcome was a composite of PD, AD and dementia.

Another study proposed a wide-deep neural network to predict progression from mild cognitive impairment to Alzheimer’s disease and had a C index = 0.78 [38]. This analysis combined a deep component (image as input) with complex latent analysis and a linear component (categorical data). They used a loss function to consider that data were censored and had a loss to follow-up. Although our objective was to predict neurodegenerative disease and not the transition, we used the same methodology, the use of a loss function, which is an extension of Cox proportional hazard models [39], for dealing with right censored events.

Cremers et al. validated a disease state index in a general population cohort to predict cognitive decline. They found that the best predictor was chronological age [40]. The model’s performance was an AUC = 0.78 for all included variables (images, epidemiologic and genetic data). We also found that chronological age was the best predictor in the CoxSf model and we had a similar discriminative ability to predict NDs.

We found that self-reported poor hearing was one of the final predictors for NDs in the CoxSf model. A case-control study in Taiwan showed a 39% higher risk of AD in those participants with hearing loss [41]. Some possible mechanisms that could explain this association are decreased cognitive stimulation due to an acoustically impoverished environment and a critical interaction of hearing loss with cognitive function in the medial temporal lobe [42]. In PD, hearing loss is recently considered as another non-motor symptom [43]. A study showed that people with hearing loss have a 77% higher risk of developing PD [44].

We found that a higher BMI was associated with a lower NDs risk. While some studies show an association between obesity in middle age and a higher risk of dementia, other studies show that being overweight is protective of cognition in older people [45]. A recent meta-analysis found that the pooled hazard ratio (95% confidence intervals) for PD in underweight participants was 1.20 (1.10, 1.30), for dementia in underweight and overweight participants was 1.23 (1.05, 1.45) and 0.88 (0.83, 0.94), respectively [46].

We observed that weight loss was associated with a higher NDs risk. A study including 2,815,135 participants from the general population and free of PD at the baseline, showed a prospective association of variations of weight loss and incidence of PD [47]. Weight loss is also associated with a higher risk of AD (45)32. The probable reason for this association is that weight loss may indicate illness.

Our results confirm the association of lower gait speed with NDs [48]. A study with 8699 participants over 60 years showed an increased risk of developing dementia when simultaneously decreased gait-speed and cognition (pooled hazard ratio, 6.28 [95% CI, 4.56–8.64]) [49]. A possible explanation may be the shared brain areas of cognition and mobility [50].

Memory function was the second most important feature analysed with SHAP after age for all DNN models and showed, in addition, the strongest protective association for NDs in the CoxSf model. We found that memory function was the best predictor of NDD after age, and its association with NDDs events was more robust than that observed with executive function. The possible explanation could be differences in whether memory or executive function precede each other in the onset of NDs [51].

We also found that self-reported vigorous physical activity was associated with a lower incidence of NDs. Our findings are in line with previous studies. The possible mechanisms related to these potential neuroprotective effects of physical activity for preventing NDs are reducing neuro-inflammation, insulin resistance, stress and anxiety [52].

Goerdten et al. performed a systematic review of statistical methods for dementia prediction. They described the most common weakness of studies on dementia prediction [53]. One of the weaknesses in prediction with ML studies was the use of data from populations with a more significant proportion of cases. Our study’s data source is a representative sample of older people in England. Therefore, it was not oversampled with cases. Another issue of many studies was the poor assumption of the Cox models. Again, our analysis verified these assumptions. Finally, they described the lack of external validation. In this latter case, this study was not validated in a different dataset.

The feature importance results showed features in common with the three DNNs and Cox models. In all models (DNNs and CoxSf), memory function and vigorous physical activity were the most crucial variables to predict NDs after age. In the case of memory function index, the best association with the outcome was with a lower memory function index associated with a higher risk of NDs rather than a high value associated with a lower risk, which suggests the importance of reporting lower memory index values in the population at risk of NDs. Notably, vigorous physical activity was one of the ten features in the DNNs models. These results agree with studies on preventing Parkinson’s [54] and Alzheimer’s disease [55]. The mechanisms are likely due to a lower decline in microstructural brain temporal areas [56]. Moreover, other features assessing physical functioning were represented in the models, such as gait speed and chair rise outcome, which supports the role of physical functioning in evaluating the risk in the general older population.

Our study has several strengths, as (i) we analysed the ELSA study, which is high quality and well-suited for our objectives, (ii) we analysed three DNNs models using a reproducible methodology and (iii) the model assessment was comprehensive, including time-independent and time-dependent measures, overfitting, stability and robustness.

This study has some limitations. We could not analyse the outcomes separately to achieve acceptable robustness due to the available sample size and the number of ND events in the ELSA study. Therefore, the selected features are a proportion of the selected diseases. The consequence is that our model is only applicable in the general older population with an equal balance of the analysed NDs. In addition, there was a loss to follow-up, and we had no information about its causes. However, we used methods to deal with this issue (Cox models and loss function in NL models). Another limitation was that the predictors and the outcome were self-reported, and therefore, recall bias may be an issue. Another issue was that independent evaluations using other data sets or populations were not performed and are needed. Further limitations are that we analysed only baseline information and no time-varying predictors and did not add a calibration measure for the prediction models.

Future research should focus on the external validation of these algorithms in larger datasets and combining different features from genetic data, surveys, images and sound.

## Conclusions

We demonstrated that it is possible to predict NDs in the older general population and that performance of Tabtransfomer seems better than other NDD for tabular data. TabTransformer, a type of DNN, can be an alternative to Cox models for predicting ND in population cohort studies and is more suited for numerous features. In contrast, Cox models are easier to interpret but challenging to implement with many candidate predictors. TabTransformer combines the advantages of other structures such as convolutional and recurrent networks and improves modelling by considering the surrounding context. Moreover, it can integrate categorical input in addition to numerical features and handle a loss to follow-up and participants’ dropout because it is modelled. These characteristics make this structure promising for complex, heterogeneous data survival analyses where there are numerous features than can be considered potential predictors. Tabtransformer could be applicable and the preferred choice over Cox models for combining tabular and not tabular data (for example, images).

## Supplementary Information

**Additional file 1: Supplementary Table 1.** Features candidates to predictors to be included in the models. **Supplementary Table 2.** Comparison between imputed and non-imputed original values. **Supplementary Table 3.** Uno’s C Statistics mean and 95% confidence intervals. **Supplementary Table 4.** Time-dependent AUC mean and 95% confidence intervals. **Supplementary Table 5.** Time-dependent balanced accuracy mean and 95% confidence intervals. **Supplementary Table 6.** Time-dependent sensitivity mean and 95% confidence intervals. **Supplementary Table 7.** Time-dependent specificity mean and 95% confidence intervals. **Supplementary Fig. 1.** Flowchart of participant’s selection at baseline (2004–2005) and attrition from 2004 to 2005 to 2016–2017. The English Longitudinal Study of Ageing. **Supplementary Fig. 2.** Observed and imputed data: Memory and Executive scores. **Supplementary Fig. 3.** Observed and imputed data: Gait speed and BMI and Executive scores. **Supplementary Fig. 4.** Observed and imputed data: Chair rise time and pulse rate. **Supplementary Fig. 5.** Evaluation of overfitting of machine learning models predicting new events of neurodegenerative diseases. **Supplementary Fig. 6.** SHAP feature importance and summary plots in deep neural models. **Supplementary Fig. 7.** Intercept of variables among deep neural models and Cox models.

## Data Availability

The datasets generated and/or analysed during the current study and used to train and validate the models are available in UK Data service website. https://beta.ukdataservice.ac.uk/datacatalogue/series/series?id=200011. The code used in this analysis is available on GitHub. https://github.com/lzhangLIH/Neuropred.
