# Correction: Machine learning for predicting neurodegenerative diseases in the general older population: a cohort study

**DOI:** 10.1186/s12874-023-01854-3

**Published:** 2023-01-31

**Authors:** Gloria A. Aguayo, Lu Zhang, Michel Vaillant, Moses Ngari, Magali Perquin, Valerie Moran, Laetitia Huiart, Rejko Krüger, Francisco Azuaje, Cyril Ferdynus, Guy Fagherazzi

**Affiliations:** 1 Deep Digital Phenotyping Research Unit, Department of Precision Health, Luxembourg Institute of Health, Strassen, Luxembourg; 2 Bioinformatics Platform, Luxembourg Institute of Health, Strassen, Luxembourg; 3 CompetenceCenter for Methodology and Statistics, Translational Medicine Operations Hub, Luxembourg Institute of Health, Strassen, Luxembourg; 4 KEMRI/ Wellcome Trust Research Programme, Kilifi, Kenya; 5 Department of Precision Health, Luxembourg Institute of Health, Strassen, Luxembourg; 6 Living Conditions Department, Luxembourg Institute of Socio-Economic Research, Esch-Sur-Alzette, Luxembourg; 7 LCSB, Luxembourg Centre for System Biomedicine, University of Luxembourg, Esch-Sur-Alzette, Luxembourg; 8 Parkinson Research Clinic, Centre Hospitalier de Luxembourg, Luxembourg, Luxembourg; 9 Transversal Translational Medicine, Luxembourg Institute of Health, Strassen, Luxembourg; 10 Genomics England, London, UK; 11 Methodological Support Unit, Félix Guyon University Hospital Center, Saint-Denis, La Réunion France

**Correction: BMC Medical Research Methodology 23, 8 (2023)**

**https://doi.org/10.1186/s12874-023-01837-4**

Following publication of the original article [1], the author requested to change the funding source section in the article.

The original article has been updated.

## References

[1] Aguayo GA, Zhang L, Vaillant M (2023). Machine learning for predicting neurodegenerative diseases in the general older population: a cohort study. BMC Medical Research Methodology.

