# Supplementary material for: Machine learning for predicting neurodegenerative diseases in the general older population: a cohort study
Source: BMC Med Res Methodol. 2023 Jan 11;23:8. doi: 10.1186/s12874-023-01837-4 (PMC9832793; doi:10.1186/s12874-023-01837-4)

Supplementary material

Contents

[Supplementary Table 1. Features candidates to predictors to be included in the models 2](#_Toc123190835)

[Supplementary Table 2. Comparison between imputed and non-imputed original values 8](#_Toc123190836)

[Supplementary Table 3. Uno's C Statistics mean and 95% confidence intervals 9](#_Toc123190837)

[Supplementary Table 4. Time-dependent AUC mean and 95% confidence intervals 11](#_Toc123190838)

[Supplementary Table 5. Time-dependent balanced accuracy mean and 95% confidence intervals 20](#_Toc123190839)

[Supplementary Table 6. Time-dependent sensitivity mean and 95% confidence intervals 29](#_Toc123190840)

[Supplementary Table 7. Time-dependent specificity mean and 95% confidence intervals 37](#_Toc123190841)

[Supplementary Figure 1. Flowchart of participant’s selection at baseline (2004-2005) and attrition from 2004-2005 to 2016-2017. The English Longitudinal Study of Ageing 46](#_Toc123190842)

[Supplementary Figure 2. Observed and imputed data: Memory and Executive scores 47](#_Toc123190843)

[Supplementary Figure 3. Observed and imputed data: Gait speed and BMI and Executive scores 48](#_Toc123190844)

[Supplementary Figure 4. Observed and imputed data: Chair rise time and pulse rate 49](#_Toc123190845)

[Supplementary Figure 5. Evaluation of overfitting of machine learning models predicting new events of neurodegenerative diseases 50](#_Toc123190846)

[Supplementary Figure 6. SHAP feature importance and summary plots in deep neural models 51](#_Toc123190847)

[Supplementary Figure 7. Intercept of variables among deep neural models and Cox models 52](#_Toc123190848)

## Supplementary Table 1. Features candidates to predictors to be included in the models

| **Number** | **Feature** | **Definition** | **Type of predictor** | **Binary or continuous** | **Missing data (%)** | **Chosen** |
| --- | --- | --- | --- | --- | --- | --- |
| 1 | Apo E | Blood APOE level (moll/l) | Biomarker | Continuous | 32.1 | Yes |
| 2 | Best lung function | Highest technically satisfactory FEV reading (litters) | Biomarker | Continuous | 19.4 | Yes |
| 3 | Ferritin | Blood ferritin level (ng/ml) | Biomarker | Continuous | 32.0 | Yes |
| 4 | Fibrinogen | Blood fibrinogen level (g/l) | Biomarker | Continuous | 32.0 | Yes |
| 5 | HbA1c | Blood glycated haemoglobin level (%) | Biomarker | Continuous | 33.0 | Yes |
| 6 | HDL cholesterol | Blood HDL level (moll/l) | Biomarker | Continuous | 32.0 | Yes |
| 7 | LDL cholesterol | Blood LDL level (moll/l) | Biomarker | Continuous | 33.5 | Yes |
| 8 | Mean lung function | Mean 1st and 2nd PF reading (litters per minute) | Biomarker | Continuous | 20.3 | Yes |
| 9 | Microalbuminuria treatment | Whether taking ACE inhibitor or A2 receptor blockers | Biomarker | Binary | 0.2 | Yes |
| 10 | Pulse Pressure | Valid Pulse Pressure | Biomarker | Continuous | 22.0 | Yes |
| 11 | Total cholesterol | Total cholesterol moll/l | Biomarker | Continuous | 32.0 | No |
| 12 | Cognitive function (accuracy) | % Accuracy of letter search (0=worst; 100=best) | Cognition | Continuous | 3.3 | Yes |
| 13 | Executive Function Index (cognitive) | 0=worst; 23=best | Cognition | Continuous | 3.3 | Yes |
| 14 | Literacy score | Good or poor literacy score | Cognition | Binary | 3.7 | Yes |
| 15 | Memory Function Index | 0=worst; 27=best | Cognition | Continuous | 0.2 | Yes |
| 16 | Admitted to hospital | Whether admitted to hospital for heart complaint in last 6 weeks or Whether respondent had abdominal or chest surgery in last 3 weeks | Comorbidity | Binary | 11.6 | Yes |
| 17 | Arthritis | Ever reported arthritis (diagnosed) | Comorbidity | Binary | 0.0 | Yes |
| 18 | Asthma | Ever reported asthma (diagnosed) | Comorbidity | Binary | 0.0 | Yes |
| 19 | Beta-blockers | Whether taking beta-blocker | Comorbidity | Binary | 0.0 | Yes |
| 20 | Breathlessness or phlegm | Breathlessness or | Comorbidity | Binary | 8.0 | Yes |
| 21 | Cancer | Ever reported cancer (diagnosed) | Comorbidity | Binary | 0.0 | Yes |
| 22 | Cardiovascular | Self-reported diagnosis of Infarction, stroke or heart failure | Comorbidity | Binary | 0.0 | No |
| 23 | Cataract | Ever reported cataract (diagnosed) | Comorbidity | Binary | 0.0 | Yes |
| 24 | Change in health | Change in self-reported health | Comorbidity | Binary | 1.2 | Yes |
| 25 | Diabetes | Ever reported diabetes (diagnosed) | Comorbidity | Binary | 0.0 | Yes |
| 26 | Diastolic blood pressure | Measured diastolic blood pressure (mm Hg) | Comorbidity | Continuous | 22.0 | Yes |
| 27 | Heart attack | Ever reported myocardial infarction (diagnosed) | Comorbidity | Binary | 0.0 | Yes |
| 28 | High blood pressure | Measured systolic blood pressure >=140 or diastolic blood pressures>=90 or taking blood pressure medications | Comorbidity | Binary | 18.2 | Yes |
| 29 | Hip fracture | Whether fractured hip or whether had joint replacement | Comorbidity | Binary | 0.1 | Yes |
| 30 | Incontinence | Whether lost urine beyond control in last 12 months | Comorbidity | Binary | 0.1 | Yes |
| 31 | Longstanding illness | Whether has self-reported long-standing illness | Comorbidity | Binary | 0.1 | Yes |
| 32 | Lung disease | Lung disease diagnosis newly reported | Comorbidity | Binary | 0.0 | Yes |
| 33 | Macular degeneration | Eye: macular degeneration diagnosis newly reported | Comorbidity | Binary | 0.0 | Yes |
| 34 | Medication for high blood pressure | High blood pressure: whether taking medication | Comorbidity | Binary | 0.0 | Yes |
| 35 | Medications for asthma | Whether taking medication for asthma | Comorbidity | Binary | 0.0 | Yes |
| 36 | Poor hearing | Self-reported hearing (while using hearing aid if appropriate) | Comorbidity | Binary | 0.0 | Yes |
| 37 | Poor vision | Whether has difficulty with vision, or ever reported (diagnosed): cataracts, glaucoma, diabetic eye or macular degeneration | Comorbidity | Binary | 0.0 | Yes |
| 38 | Retinopathy | Ever reported diabetic eye disease (diagnosed) | Comorbidity | Binary | 0.0 | Yes |
| 39 | Self-reported hypertension | Ever reported high blood pressure (diagnosed) | Comorbidity | Binary | 0.0 | Yes |
| 40 | Self-reported poor health | Fair or poor self-reported health | Comorbidity | Binary | 0.0 | Yes |
| 41 | Stroke | Ever reported stroke (diagnosed) | Comorbidity | Binary | 0.0 | Yes |
| 42 | Systolic blood pressure | Systolic pressure (mm Hg) | Comorbidity | Continuous | 22.0 | No |
| 43 | Age | Age (years) | Demographic | Continuous | 0.0 | Yes |
| 44 | Marital status | Married or cohabiting | Demographic | Binary | 0.0 | Yes |
| 45 | Sex | Sex | Demographic | Binary | 0.0 | Yes |
| 46 | Difficulty cooking | Difficulty preparing a hot meal | Disability | Binary | 0.0 | Yes |
| 47 | Difficulty taking medications | Difficulty taking medications | Disability | Binary | 0.0 | Yes |
| 48 | Difficulty using map | Difficulty using map to figure out how to get around strange place | Disability | Binary | 0.0 | Yes |
| 49 | Difficulty with money | Difficulty managing money, e.g. paying bills, keeping track expenses | Disability | Binary | 0.0 | Yes |
| 50 | Limiting illness | Limiting long-standing illness | Disability | Binary | 0.1 | Yes |
| 51 | Mobility difficulties | Difficulty getting up from chair after sitting long periods | Disability | Binary | 0.0 | Yes |
| 52 | Coal use | Whether uses coal | Environmental | Binary | 0.0 | Yes |
| 53 | Electricity use | Whether uses electricity | Environmental | Binary | 0.0 | Yes |
| 54 | Gas use | Whether uses gas | Environmental | Binary | 0.0 | Yes |
| 55 | Oil use | Whether uses oil | Environmental | Binary | 0.0 | Yes |
| 56 | Other use | Whether uses other fuel | Environmental | Binary | 0.0 | Yes |
| 57 | Paraffin use | Whether uses paraffin | Environmental | Binary | 0.0 | Yes |
| 58 | Wood use | Whether uses wood | Environmental | Binary | 0.0 | Yes |
| 59 | Body Mass Index (BMI) | Weight/Height*Height | Lifestyle | Continuous | 14.7 | Yes |
| 60 | Excessive alcohol | Alcohol consumption >= 2 units/day | Lifestyle | Binary | 29.9 | Yes |
| 61 | Sedentary | Low or sedentary physical activity level | Lifestyle | Binary | 0.1 | Yes |
| 62 | Sleep | Whether respondent felt their sleep was restless during the past week | Lifestyle | Binary | 0.4 | Yes |
| 63 | Smoker (current) | Current smoker | Lifestyle | Binary | 0.1 | Yes |
| 64 | Vigorous physical activity | Reported vigorous physical activity once a week | Lifestyle | Binary | 0.0 | Yes |
| 65 | Waist | Waist circumference (cm) | Lifestyle | Continuous | 13.8 | Yes |
| 66 | Chair rise outcome | Single chair rise outcome | Physical functioning | Binary | 22.4 | Yes |
| 67 | Chair rise score | Combined chair rise outcome & time | Physical functioning | Binary | 22.4 | Yes |
| 68 | Chair rise time | Time to complete 5 rises (seconds) | Physical functioning | Continuous | 28.0 | Yes |
| 69 | Gait speed | Mean gait speed (m/s) | Physical functioning | Continuous | 9.1 | Yes |
| 70 | Grip strength | Maximum grip strength (kg) | Physical functioning | Continuous | 12.9 | Yes |
| 71 | Poor balance | Results in balance test | Physical functioning | Binary | 11.7 | Yes |
| 72 | Anxiety | Self-reported anxiety as psychiatric problem | Psychosocial | Binary | 0.0 | Yes |
| 73 | Depression | Depression symptoms with 8-item of CES-D (cut-off >=4) | Psychosocial | Binary | 1.0 | Yes |
| 74 | Emotional distress | Self-reported emotional problem | Psychosocial | Binary | 0.0 | Yes |
| 75 | Happy mood | Whether respondent was happy much of the time during the past week | Psychosocial | Binary | 0.6 | Yes |
| 76 | Lack of social network | Whether the respondent does not have any friends | Psychosocial | Binary | 10.7 | Yes |
| 77 | Living alone | Whether does not have a husband, wife or partner with whom they live | Psychosocial | Binary | 12.0 | No |
| 78 | Loss partner | Respondent had a spouse who died since last interview | Psychosocial | Binary | 0.0 | Yes |
| 79 | Low income | Whether participant is in the lowest income tertile | Psychosocial | Binary | 1.0 | Yes |
| 80 | Mood Swings | Reported mood swings | Psychosocial | Binary | 0.0 | Yes |
| 81 | Non educational qualification | No educational qualification or foreigner | Psychosocial | Binary | 0.1 | Yes |
| 82 | Rely on friends | Not relay or no friends | Psychosocial | Binary | 11.7 | Yes |
| 83 | Self-reported psychiatric disorders | Ever reported psychiatric disorder (diagnosed) | Psychosocial | Binary | 0.0 | Yes |
| 84 | Social class | Manual or non-manual | Psychosocial | Binary | 11.7 | Yes |
| 85 | Societal integration | Organizational membership: not a member of any organization, club or society | Psychosocial | Binary | 16.8 | Yes |
| 86 | Subjective social status | Self-perceived social status of respondent on ladder | Psychosocial | Continuous | 14.2 | Yes |
| 87 | Breathlessness | Breathlessness, MRC respiratory questionnaire | Symptoms | Binary | 10.2 | Yes |
| 88 | Dizziness | Frequency of dizziness when walking on level surface | Symptoms | Categorical | 1.9 | Yes |
| 89 | Exhaustion | Whether respondent felt everything they did during the past week was an effort | Symptoms | Binary | 0.3 | Yes |
| 90 | Falls | Whether fallen down | Symptoms | Binary | 0.1 | Yes |
| 91 | Fine motor difficulties | Difficulty picking up coin from table | Symptoms | Binary | 0.0 | Yes |
| 92 | Glaucoma | Ever reported glaucoma (diagnosed) | Symptoms | Binary | 0.0 | Yes |
| 93 | Number of falls | Number times have fallen down | Symptoms | Continuous | 0.3 | Yes |
| 94 | Pain | Whether often troubled with pain | Symptoms | Binary | 0.0 | Yes |
| 95 | Weight loss | Weight loss (more than 5 kg) | Symptoms | Binary | 14.5 | Yes |

## Supplementary Table 2. Comparison between imputed and non-imputed original values

| Continuous variables | | | | |
| --- | --- | --- | --- | --- |
| Variable | Mean incomplete data | Mean imputed data | Difference* | % bias** |
| LDL cholesterol | 3.59 | 3.58 | 0.01 | -0.1 |
| HbA1c | 5.60 | 5.62 | -0.02 | 0.4 |
| ApoE | 0.04 | 0.04 | 0.00 | 0.4 |
| Ferritin | 120.44 | 120.96 | -0.52 | 0.4 |
| Fibrinogen | 3.22 | 3.24 | -0.02 | 0.6 |
| HDL cholesterol | 1.53 | 1.52 | 0.00 | -0.3 |
| Total cholesterol | 5.91 | 5.90 | 0.01 | -0.2 |
| Chair rise time | 11.66 | 12.55 | -0.89 | 7.1 |
| Diastolic blood pressure | 75.03 | 75.57 | -0.55 | 0.7 |
| Pulse | 60.10 | 61.13 | -1.04 | 1.7 |
| Systolic blood pressure | 135.12 | 136.59 | -1.47 | 1.1 |
| Peak flow | 328.80 | 322.50 | 6.30 | -2.0 |
| htfev | 2.31 | 2.28 | 0.03 | -1.5 |
| BMI | 27.88 | 27.95 | -0.07 | 0.2 |
| Social class score | 58.97 | 58.42 | 0.55 | -0.9 |
| Waist circumference | 95.65 | 95.67 | -0.02 | 0.0 |
| Grip strength | 30.29 | 30.06 | 0.23 | -0.8 |
| Gait speed | 0.86 | 0.91 | -0.05 | 5.6 |
| Executive score | 13.10 | 13.02 | 0.08 | -0.6 |
| Falls | 0.65 | 0.65 | 0.00 | 0.6 |
| Memory score | 15.72 | 15.71 | 0.01 | -0.1 |

*Difference between expected and value in the incomplete dataset

**% bias: 100*(mean imputed-mean incomplete)/mean imputed

## Supplementary Table 3. Uno's C Statistics mean and 95% confidence intervals

|  | CoxSf | | | CoxEn | | | Feedforward | | | Densenet | | | Tab transformer | | |
| --- | --- | --- | --- | --- | --- | --- | --- | --- | --- | --- | --- | --- | --- | --- | --- |
| imp | mean | 95% lci | 95% uci | mean | 95% lci | 95% uci | mean | 95% lci | 95% uci | mean | 95% lci | 95% uci | mean | 95% lci | 95% uci |
| 1 | 0.734 | 0.694 | 0.772 | 0.729 | 0.683 | 0.769 | 0.709 | 0.644 | 0.752 | 0.706 | 0.643 | 0.758 | 0.752 | 0.710 | 0.838 |
| 2 | 0.736 | 0.691 | 0.763 | 0.731 | 0.683 | 0.770 | 0.711 | 0.657 | 0.757 | 0.709 | 0.663 | 0.751 | 0.762 | 0.715 | 0.839 |
| 3 | 0.733 | 0.703 | 0.772 | 0.731 | 0.681 | 0.766 | 0.708 | 0.671 | 0.749 | 0.706 | 0.654 | 0.743 | 0.778 | 0.724 | 0.822 |
| 4 | 0.734 | 0.697 | 0.767 | 0.739 | 0.692 | 0.781 | 0.709 | 0.657 | 0.750 | 0.703 | 0.645 | 0.745 | 0.759 | 0.709 | 0.837 |
| 5 | 0.736 | 0.692 | 0.780 | 0.729 | 0.691 | 0.766 | 0.706 | 0.652 | 0.751 | 0.704 | 0.653 | 0.756 | 0.769 | 0.719 | 0.828 |
| 6 | 0.734 | 0.691 | 0.781 | 0.733 | 0.684 | 0.774 | 0.706 | 0.645 | 0.761 | 0.704 | 0.645 | 0.757 | 0.768 | 0.724 | 0.828 |
| 7 | 0.734 | 0.698 | 0.776 | 0.728 | 0.690 | 0.767 | 0.707 | 0.646 | 0.751 | 0.703 | 0.656 | 0.745 | 0.756 | 0.705 | 0.839 |
| 8 | 0.734 | 0.689 | 0.774 | 0.728 | 0.688 | 0.767 | 0.710 | 0.655 | 0.758 | 0.708 | 0.648 | 0.753 | 0.744 | 0.683 | 0.829 |
| 9 | 0.737 | 0.704 | 0.773 | 0.732 | 0.695 | 0.775 | 0.708 | 0.669 | 0.749 | 0.708 | 0.659 | 0.755 | 0.758 | 0.702 | 0.817 |
| 10 | 0.734 | 0.696 | 0.773 | 0.733 | 0.683 | 0.780 | 0.708 | 0.660 | 0.751 | 0.703 | 0.649 | 0.754 | 0.747 | 0.685 | 0.843 |
| 11 | 0.736 | 0.701 | 0.775 | 0.733 | 0.683 | 0.773 | 0.708 | 0.636 | 0.752 | 0.706 | 0.642 | 0.755 | 0.746 | 0.689 | 0.830 |
| 12 | 0.732 | 0.699 | 0.768 | 0.732 | 0.693 | 0.769 | 0.709 | 0.643 | 0.755 | 0.710 | 0.660 | 0.746 | 0.758 | 0.708 | 0.830 |
| 13 | 0.734 | 0.691 | 0.770 | 0.727 | 0.684 | 0.767 | 0.708 | 0.671 | 0.750 | 0.706 | 0.652 | 0.748 | 0.756 | 0.706 | 0.835 |
| 14 | 0.734 | 0.695 | 0.762 | 0.732 | 0.696 | 0.776 | 0.709 | 0.663 | 0.754 | 0.705 | 0.646 | 0.747 | 0.762 | 0.711 | 0.842 |
| 15 | 0.732 | 0.690 | 0.772 | 0.733 | 0.686 | 0.769 | 0.706 | 0.657 | 0.755 | 0.706 | 0.655 | 0.756 | 0.760 | 0.709 | 0.840 |
| 16 | 0.734 | 0.696 | 0.773 | 0.728 | 0.688 | 0.762 | 0.705 | 0.640 | 0.756 | 0.702 | 0.643 | 0.757 | 0.757 | 0.696 | 0.833 |
| 17 | 0.738 | 0.702 | 0.774 | 0.734 | 0.696 | 0.773 | 0.704 | 0.643 | 0.750 | 0.706 | 0.656 | 0.750 | 0.768 | 0.711 | 0.840 |
| 18 | 0.729 | 0.690 | 0.764 | 0.734 | 0.690 | 0.778 | 0.709 | 0.646 | 0.758 | 0.709 | 0.651 | 0.752 | 0.737 | 0.674 | 0.838 |
| 19 | 0.733 | 0.689 | 0.776 | 0.732 | 0.691 | 0.771 | 0.711 | 0.652 | 0.759 | 0.709 | 0.663 | 0.755 | 0.760 | 0.705 | 0.827 |
| 20 | 0.731 | 0.689 | 0.767 | 0.732 | 0.680 | 0.780 | 0.708 | 0.645 | 0.753 | 0.701 | 0.647 | 0.749 | 0.761 | 0.706 | 0.833 |
| 21 | 0.736 | 0.690 | 0.774 | 0.729 | 0.692 | 0.766 | 0.708 | 0.641 | 0.751 | 0.705 | 0.641 | 0.759 | 0.759 | 0.712 | 0.825 |
| 22 | 0.731 | 0.687 | 0.767 | 0.735 | 0.692 | 0.769 | 0.711 | 0.655 | 0.753 | 0.709 | 0.659 | 0.749 | 0.754 | 0.696 | 0.833 |
| 23 | 0.735 | 0.699 | 0.778 | 0.733 | 0.696 | 0.768 | 0.709 | 0.670 | 0.752 | 0.706 | 0.656 | 0.746 | 0.763 | 0.713 | 0.843 |
| 24 | 0.735 | 0.697 | 0.778 | 0.734 | 0.686 | 0.775 | 0.713 | 0.664 | 0.757 | 0.706 | 0.644 | 0.748 | 0.758 | 0.698 | 0.839 |
| 25 | 0.734 | 0.693 | 0.769 | 0.728 | 0.683 | 0.778 | 0.706 | 0.655 | 0.752 | 0.706 | 0.654 | 0.755 | 0.758 | 0.695 | 0.841 |
| 26 | 0.734 | 0.697 | 0.766 | 0.734 | 0.694 | 0.780 | 0.710 | 0.638 | 0.759 | 0.704 | 0.647 | 0.753 | 0.752 | 0.693 | 0.838 |
| 27 | 0.735 | 0.702 | 0.772 | 0.734 | 0.692 | 0.776 | 0.709 | 0.651 | 0.755 | 0.703 | 0.653 | 0.747 | 0.740 | 0.675 | 0.838 |
| 28 | 0.739 | 0.698 | 0.778 | 0.735 | 0.695 | 0.766 | 0.709 | 0.667 | 0.754 | 0.712 | 0.648 | 0.756 | 0.753 | 0.690 | 0.838 |
| 29 | 0.736 | 0.697 | 0.775 | 0.729 | 0.681 | 0.768 | 0.708 | 0.657 | 0.753 | 0.707 | 0.656 | 0.754 | 0.750 | 0.694 | 0.830 |
| 30 | 0.733 | 0.698 | 0.772 | 0.731 | 0.689 | 0.774 | 0.707 | 0.655 | 0.755 | 0.702 | 0.648 | 0.754 | 0.754 | 0.688 | 0.835 |
| 31 | 0.736 | 0.700 | 0.772 | 0.738 | 0.693 | 0.773 | 0.712 | 0.642 | 0.754 | 0.709 | 0.641 | 0.762 | 0.765 | 0.707 | 0.825 |
| 32 | 0.736 | 0.691 | 0.778 | 0.735 | 0.693 | 0.764 | 0.710 | 0.646 | 0.756 | 0.708 | 0.660 | 0.750 | 0.761 | 0.711 | 0.840 |
| 33 | 0.732 | 0.691 | 0.777 | 0.730 | 0.684 | 0.775 | 0.705 | 0.666 | 0.746 | 0.704 | 0.649 | 0.743 | 0.768 | 0.714 | 0.828 |
| 34 | 0.735 | 0.689 | 0.773 | 0.734 | 0.699 | 0.766 | 0.709 | 0.662 | 0.757 | 0.705 | 0.647 | 0.745 | 0.747 | 0.699 | 0.823 |
| 35 | 0.736 | 0.693 | 0.770 | 0.729 | 0.688 | 0.763 | 0.710 | 0.658 | 0.757 | 0.708 | 0.655 | 0.761 | 0.761 | 0.715 | 0.821 |
| 36 | 0.735 | 0.694 | 0.778 | 0.734 | 0.697 | 0.770 | 0.706 | 0.641 | 0.761 | 0.705 | 0.648 | 0.757 | 0.753 | 0.696 | 0.836 |
| 37 | 0.732 | 0.690 | 0.771 | 0.724 | 0.677 | 0.764 | 0.704 | 0.652 | 0.753 | 0.706 | 0.658 | 0.751 | 0.753 | 0.701 | 0.843 |
| 38 | 0.734 | 0.686 | 0.774 | 0.732 | 0.685 | 0.775 | 0.709 | 0.640 | 0.753 | 0.710 | 0.651 | 0.754 | 0.759 | 0.696 | 0.836 |
| 39 | 0.734 | 0.691 | 0.772 | 0.732 | 0.686 | 0.779 | 0.708 | 0.641 | 0.757 | 0.707 | 0.654 | 0.754 | 0.748 | 0.696 | 0.831 |
| 40 | 0.731 | 0.686 | 0.768 | 0.728 | 0.696 | 0.759 | 0.705 | 0.671 | 0.742 | 0.701 | 0.646 | 0.749 | 0.756 | 0.714 | 0.831 |
| **Total mean** and 95% CI | **0.734** | 0.694 | 0.772 | **0.732** | 0.689 | 0.771 | **0.708** | 0.653 | 0.754 | **0.706** | 0.651 | 0.752 | **0.757** | 0.702 | 0.834 |

Abbreviations: CoxSf: Cox models with selected features; CoxEn: Cox models with Elastic Net regularization; FeedForward: Feedforward neural network; Densenet: Densely Connected Convolutional Network; TabTransformer: TabTransformer neural network; imp: imputation number; LCI: 95% lower confidence interval; UCI: 95% upper confidence interval; 95% CI: 95% confidence intervals

## Supplementary Table 4. Time-dependent AUC mean and 95% confidence intervals

| 4-year AUC | | | | | | | | | | | | | | | |  |
| --- | --- | --- | --- | --- | --- | --- | --- | --- | --- | --- | --- | --- | --- | --- | --- | --- |
|  | CoxSf | | | CoxEn | | | Feedforward | | | Densenet | | | Tab transformer | | | |
| imp | mean | 95% lci | 95% uci | mean | 95% lci | 95% uci | mean | 95% lci | 95% uci | mean | 95% lci | 95% uci | mean | 95% lci | 95% uci | |
| 1 | 0.876 | 0.832 | 0.921 | 0.877 | 0.823 | 0.924 | 0.870 | 0.823 | 0.921 | 0.867 | 0.819 | 0.919 | 0.880 | 0.839 | 0.935 | |
| 2 | 0.875 | 0.826 | 0.929 | 0.879 | 0.834 | 0.927 | 0.865 | 0.812 | 0.922 | 0.866 | 0.816 | 0.920 | 0.885 | 0.821 | 0.928 | |
| 3 | 0.875 | 0.832 | 0.917 | 0.877 | 0.828 | 0.929 | 0.864 | 0.810 | 0.912 | 0.867 | 0.821 | 0.912 | 0.874 | 0.821 | 0.926 | |
| 4 | 0.878 | 0.837 | 0.920 | 0.878 | 0.835 | 0.924 | 0.870 | 0.828 | 0.914 | 0.868 | 0.828 | 0.911 | 0.890 | 0.841 | 0.932 | |
| 5 | 0.879 | 0.827 | 0.924 | 0.879 | 0.832 | 0.924 | 0.870 | 0.816 | 0.912 | 0.868 | 0.817 | 0.912 | 0.879 | 0.816 | 0.931 | |
| 6 | 0.878 | 0.834 | 0.925 | 0.876 | 0.830 | 0.914 | 0.867 | 0.812 | 0.914 | 0.872 | 0.824 | 0.919 | 0.877 | 0.825 | 0.924 | |
| 7 | 0.882 | 0.835 | 0.925 | 0.877 | 0.828 | 0.921 | 0.870 | 0.817 | 0.923 | 0.875 | 0.829 | 0.922 | 0.879 | 0.835 | 0.931 | |
| 8 | 0.880 | 0.839 | 0.927 | 0.873 | 0.826 | 0.920 | 0.864 | 0.808 | 0.915 | 0.871 | 0.827 | 0.915 | 0.884 | 0.844 | 0.932 | |
| 9 | 0.878 | 0.830 | 0.917 | 0.878 | 0.828 | 0.918 | 0.863 | 0.805 | 0.911 | 0.866 | 0.807 | 0.918 | 0.880 | 0.820 | 0.930 | |
| 10 | 0.877 | 0.829 | 0.928 | 0.871 | 0.830 | 0.916 | 0.869 | 0.831 | 0.910 | 0.867 | 0.806 | 0.937 | 0.887 | 0.842 | 0.936 | |
| 11 | 0.881 | 0.830 | 0.932 | 0.876 | 0.816 | 0.918 | 0.868 | 0.811 | 0.918 | 0.867 | 0.813 | 0.921 | 0.885 | 0.827 | 0.936 | |
| 12 | 0.875 | 0.834 | 0.920 | 0.881 | 0.831 | 0.922 | 0.861 | 0.806 | 0.916 | 0.859 | 0.802 | 0.912 | 0.876 | 0.820 | 0.919 | |
| 13 | 0.877 | 0.834 | 0.925 | 0.879 | 0.835 | 0.921 | 0.868 | 0.811 | 0.915 | 0.870 | 0.818 | 0.919 | 0.880 | 0.833 | 0.925 | |
| 14 | 0.874 | 0.827 | 0.912 | 0.881 | 0.825 | 0.929 | 0.870 | 0.833 | 0.915 | 0.867 | 0.823 | 0.907 | 0.881 | 0.832 | 0.918 | |
| 15 | 0.873 | 0.824 | 0.921 | 0.880 | 0.835 | 0.921 | 0.872 | 0.814 | 0.920 | 0.871 | 0.821 | 0.912 | 0.884 | 0.822 | 0.932 | |
| 16 | 0.876 | 0.825 | 0.922 | 0.876 | 0.825 | 0.922 | 0.865 | 0.807 | 0.911 | 0.873 | 0.828 | 0.921 | 0.886 | 0.824 | 0.935 | |
| 17 | 0.873 | 0.819 | 0.924 | 0.870 | 0.826 | 0.916 | 0.866 | 0.813 | 0.919 | 0.870 | 0.823 | 0.918 | 0.883 | 0.825 | 0.939 | |
| 18 | 0.879 | 0.840 | 0.921 | 0.876 | 0.833 | 0.922 | 0.870 | 0.827 | 0.913 | 0.869 | 0.822 | 0.920 | 0.875 | 0.832 | 0.935 | |
| 19 | 0.879 | 0.831 | 0.929 | 0.884 | 0.835 | 0.929 | 0.871 | 0.815 | 0.915 | 0.871 | 0.814 | 0.919 | 0.892 | 0.832 | 0.936 | |
| 20 | 0.882 | 0.847 | 0.926 | 0.882 | 0.832 | 0.920 | 0.867 | 0.812 | 0.918 | 0.870 | 0.815 | 0.932 | 0.884 | 0.830 | 0.932 | |
| 21 | 0.878 | 0.833 | 0.918 | 0.877 | 0.820 | 0.926 | 0.870 | 0.826 | 0.918 | 0.868 | 0.823 | 0.920 | 0.884 | 0.842 | 0.922 | |
| 22 | 0.873 | 0.821 | 0.923 | 0.883 | 0.839 | 0.925 | 0.869 | 0.816 | 0.925 | 0.869 | 0.814 | 0.927 | 0.876 | 0.824 | 0.942 | |
| 23 | 0.882 | 0.824 | 0.937 | 0.884 | 0.829 | 0.922 | 0.869 | 0.816 | 0.912 | 0.872 | 0.820 | 0.915 | 0.879 | 0.825 | 0.926 | |
| 24 | 0.881 | 0.831 | 0.925 | 0.878 | 0.831 | 0.928 | 0.870 | 0.833 | 0.915 | 0.867 | 0.822 | 0.908 | 0.870 | 0.810 | 0.934 | |
| 25 | 0.878 | 0.830 | 0.921 | 0.880 | 0.834 | 0.934 | 0.873 | 0.816 | 0.921 | 0.872 | 0.817 | 0.915 | 0.892 | 0.828 | 0.935 | |
| 26 | 0.878 | 0.836 | 0.925 | 0.879 | 0.824 | 0.921 | 0.866 | 0.815 | 0.912 | 0.872 | 0.825 | 0.918 | 0.880 | 0.828 | 0.933 | |
| 27 | 0.874 | 0.818 | 0.915 | 0.872 | 0.822 | 0.919 | 0.863 | 0.807 | 0.916 | 0.871 | 0.827 | 0.923 | 0.872 | 0.822 | 0.921 | |
| 28 | 0.877 | 0.835 | 0.927 | 0.878 | 0.829 | 0.922 | 0.872 | 0.818 | 0.921 | 0.878 | 0.835 | 0.925 | 0.886 | 0.844 | 0.930 | |
| 29 | 0.878 | 0.836 | 0.927 | 0.878 | 0.831 | 0.924 | 0.868 | 0.829 | 0.912 | 0.866 | 0.808 | 0.917 | 0.878 | 0.820 | 0.935 | |
| 30 | 0.876 | 0.822 | 0.919 | 0.876 | 0.829 | 0.921 | 0.866 | 0.813 | 0.913 | 0.867 | 0.806 | 0.932 | 0.880 | 0.837 | 0.931 | |
| 31 | 0.876 | 0.825 | 0.928 | 0.882 | 0.840 | 0.923 | 0.874 | 0.827 | 0.923 | 0.873 | 0.824 | 0.924 | 0.884 | 0.842 | 0.934 | |
| 32 | 0.878 | 0.831 | 0.922 | 0.884 | 0.840 | 0.926 | 0.869 | 0.808 | 0.924 | 0.865 | 0.814 | 0.921 | 0.883 | 0.840 | 0.932 | |
| 33 | 0.874 | 0.835 | 0.924 | 0.878 | 0.830 | 0.925 | 0.871 | 0.815 | 0.916 | 0.870 | 0.822 | 0.916 | 0.886 | 0.846 | 0.935 | |
| 34 | 0.876 | 0.839 | 0.929 | 0.878 | 0.830 | 0.917 | 0.870 | 0.836 | 0.911 | 0.865 | 0.819 | 0.907 | 0.879 | 0.837 | 0.928 | |
| 35 | 0.877 | 0.831 | 0.924 | 0.877 | 0.841 | 0.927 | 0.870 | 0.815 | 0.914 | 0.868 | 0.825 | 0.910 | 0.880 | 0.830 | 0.925 | |
| 36 | 0.879 | 0.841 | 0.927 | 0.880 | 0.832 | 0.928 | 0.868 | 0.821 | 0.918 | 0.871 | 0.826 | 0.921 | 0.890 | 0.829 | 0.938 | |
| 37 | 0.874 | 0.828 | 0.911 | 0.875 | 0.825 | 0.923 | 0.869 | 0.819 | 0.917 | 0.871 | 0.821 | 0.919 | 0.877 | 0.830 | 0.924 | |
| 38 | 0.875 | 0.828 | 0.917 | 0.876 | 0.820 | 0.922 | 0.863 | 0.817 | 0.910 | 0.863 | 0.820 | 0.913 | 0.875 | 0.825 | 0.927 | |
| 39 | 0.876 | 0.828 | 0.930 | 0.878 | 0.829 | 0.921 | 0.862 | 0.808 | 0.917 | 0.863 | 0.803 | 0.913 | 0.877 | 0.828 | 0.927 | |
| 40 | 0.881 | 0.834 | 0.924 | 0.877 | 0.833 | 0.919 | 0.867 | 0.808 | 0.914 | 0.869 | 0.812 | 0.938 | 0.883 | 0.824 | 0.947 | |
| **Total mean** and 95% CI | **0.877** | 0.831 | 0.923 | **0.878** | 0.830 | 0.923 | **0.868** | 0.817 | 0.916 | **0.869** | 0.819 | 0.919 | **0.881** | 0.830 | 0.931 | |
| 6-year AUC | | | | | | | | | | | | | | | |  |
|  | CoxSf | | | CoxEn | | | Feedforward | | | Densenet | | | Tab transformer | | | |
| imp | mean | 95% lci | 95% uci | mean | 95% lci | 95% uci | mean | 95% lci | 95% uci | mean | 95% lci | 95% uci | mean | 95% lci | 95% uci | |
| 1 | 0.880 | 0.847 | 0.905 | 0.879 | 0.845 | 0.910 | 0.864 | 0.821 | 0.897 | 0.865 | 0.837 | 0.896 | 0.870 | 0.841 | 0.898 | |
| 2 | 0.879 | 0.844 | 0.914 | 0.879 | 0.853 | 0.913 | 0.859 | 0.821 | 0.902 | 0.864 | 0.824 | 0.897 | 0.869 | 0.835 | 0.896 | |
| 3 | 0.876 | 0.847 | 0.901 | 0.878 | 0.850 | 0.907 | 0.856 | 0.822 | 0.889 | 0.863 | 0.832 | 0.895 | 0.865 | 0.831 | 0.904 | |
| 4 | 0.879 | 0.848 | 0.907 | 0.878 | 0.846 | 0.907 | 0.859 | 0.824 | 0.887 | 0.865 | 0.837 | 0.896 | 0.871 | 0.836 | 0.905 | |
| 5 | 0.878 | 0.842 | 0.903 | 0.876 | 0.846 | 0.911 | 0.858 | 0.820 | 0.894 | 0.861 | 0.829 | 0.893 | 0.867 | 0.832 | 0.902 | |
| 6 | 0.882 | 0.851 | 0.912 | 0.879 | 0.851 | 0.906 | 0.862 | 0.826 | 0.890 | 0.868 | 0.838 | 0.904 | 0.866 | 0.826 | 0.897 | |
| 7 | 0.878 | 0.849 | 0.909 | 0.877 | 0.841 | 0.913 | 0.861 | 0.818 | 0.894 | 0.866 | 0.837 | 0.900 | 0.870 | 0.840 | 0.905 | |
| 8 | 0.879 | 0.851 | 0.904 | 0.878 | 0.843 | 0.908 | 0.860 | 0.818 | 0.899 | 0.865 | 0.835 | 0.891 | 0.871 | 0.842 | 0.895 | |
| 9 | 0.879 | 0.850 | 0.910 | 0.879 | 0.851 | 0.910 | 0.854 | 0.818 | 0.891 | 0.859 | 0.826 | 0.894 | 0.873 | 0.837 | 0.906 | |
| 10 | 0.880 | 0.846 | 0.917 | 0.876 | 0.850 | 0.911 | 0.861 | 0.831 | 0.885 | 0.864 | 0.836 | 0.907 | 0.874 | 0.843 | 0.903 | |
| 11 | 0.879 | 0.845 | 0.912 | 0.878 | 0.837 | 0.906 | 0.860 | 0.817 | 0.894 | 0.864 | 0.838 | 0.896 | 0.872 | 0.834 | 0.901 | |
| 12 | 0.877 | 0.850 | 0.906 | 0.879 | 0.852 | 0.909 | 0.856 | 0.810 | 0.891 | 0.860 | 0.820 | 0.895 | 0.868 | 0.833 | 0.894 | |
| 13 | 0.880 | 0.847 | 0.909 | 0.879 | 0.856 | 0.905 | 0.855 | 0.818 | 0.891 | 0.862 | 0.829 | 0.895 | 0.873 | 0.845 | 0.907 | |
| 14 | 0.880 | 0.852 | 0.910 | 0.880 | 0.844 | 0.907 | 0.861 | 0.831 | 0.887 | 0.866 | 0.841 | 0.896 | 0.864 | 0.832 | 0.897 | |
| 15 | 0.877 | 0.850 | 0.909 | 0.880 | 0.849 | 0.915 | 0.863 | 0.823 | 0.890 | 0.866 | 0.830 | 0.896 | 0.869 | 0.834 | 0.900 | |
| 16 | 0.877 | 0.840 | 0.912 | 0.878 | 0.849 | 0.901 | 0.859 | 0.829 | 0.887 | 0.868 | 0.834 | 0.902 | 0.871 | 0.843 | 0.910 | |
| 17 | 0.880 | 0.846 | 0.915 | 0.877 | 0.846 | 0.904 | 0.857 | 0.825 | 0.889 | 0.864 | 0.833 | 0.899 | 0.868 | 0.844 | 0.903 | |
| 18 | 0.882 | 0.851 | 0.912 | 0.879 | 0.850 | 0.903 | 0.859 | 0.824 | 0.893 | 0.863 | 0.834 | 0.889 | 0.872 | 0.843 | 0.902 | |
| 19 | 0.883 | 0.854 | 0.917 | 0.883 | 0.849 | 0.910 | 0.861 | 0.821 | 0.893 | 0.862 | 0.828 | 0.895 | 0.874 | 0.847 | 0.900 | |
| 20 | 0.880 | 0.846 | 0.912 | 0.881 | 0.850 | 0.910 | 0.860 | 0.822 | 0.902 | 0.862 | 0.829 | 0.904 | 0.870 | 0.833 | 0.905 | |
| 21 | 0.878 | 0.848 | 0.907 | 0.880 | 0.847 | 0.909 | 0.864 | 0.819 | 0.894 | 0.866 | 0.839 | 0.894 | 0.871 | 0.837 | 0.902 | |
| 22 | 0.876 | 0.843 | 0.906 | 0.877 | 0.845 | 0.906 | 0.858 | 0.818 | 0.898 | 0.863 | 0.820 | 0.899 | 0.865 | 0.836 | 0.900 | |
| 23 | 0.881 | 0.845 | 0.906 | 0.880 | 0.842 | 0.915 | 0.857 | 0.822 | 0.893 | 0.865 | 0.832 | 0.893 | 0.869 | 0.834 | 0.900 | |
| 24 | 0.885 | 0.857 | 0.922 | 0.885 | 0.859 | 0.911 | 0.863 | 0.832 | 0.887 | 0.867 | 0.838 | 0.896 | 0.871 | 0.839 | 0.908 | |
| 25 | 0.877 | 0.847 | 0.909 | 0.881 | 0.849 | 0.911 | 0.858 | 0.817 | 0.887 | 0.864 | 0.831 | 0.891 | 0.867 | 0.835 | 0.901 | |
| 26 | 0.883 | 0.851 | 0.909 | 0.879 | 0.852 | 0.904 | 0.863 | 0.822 | 0.892 | 0.870 | 0.838 | 0.903 | 0.872 | 0.828 | 0.905 | |
| 27 | 0.876 | 0.845 | 0.910 | 0.874 | 0.840 | 0.904 | 0.859 | 0.817 | 0.902 | 0.864 | 0.831 | 0.897 | 0.869 | 0.835 | 0.900 | |
| 28 | 0.879 | 0.848 | 0.904 | 0.881 | 0.853 | 0.910 | 0.863 | 0.830 | 0.897 | 0.869 | 0.839 | 0.894 | 0.871 | 0.841 | 0.906 | |
| 29 | 0.878 | 0.845 | 0.906 | 0.880 | 0.847 | 0.908 | 0.858 | 0.828 | 0.884 | 0.859 | 0.828 | 0.895 | 0.865 | 0.836 | 0.896 | |
| 30 | 0.878 | 0.844 | 0.901 | 0.876 | 0.846 | 0.899 | 0.857 | 0.816 | 0.886 | 0.860 | 0.828 | 0.903 | 0.863 | 0.837 | 0.898 | |
| 31 | 0.876 | 0.841 | 0.908 | 0.878 | 0.851 | 0.908 | 0.861 | 0.818 | 0.896 | 0.865 | 0.840 | 0.898 | 0.866 | 0.835 | 0.912 | |
| 32 | 0.881 | 0.849 | 0.909 | 0.881 | 0.855 | 0.909 | 0.860 | 0.820 | 0.904 | 0.863 | 0.824 | 0.897 | 0.870 | 0.841 | 0.908 | |
| 33 | 0.877 | 0.848 | 0.905 | 0.878 | 0.849 | 0.909 | 0.859 | 0.826 | 0.893 | 0.866 | 0.835 | 0.897 | 0.875 | 0.841 | 0.907 | |
| 34 | 0.878 | 0.851 | 0.914 | 0.876 | 0.848 | 0.907 | 0.862 | 0.830 | 0.887 | 0.867 | 0.841 | 0.897 | 0.868 | 0.833 | 0.902 | |
| 35 | 0.879 | 0.852 | 0.908 | 0.877 | 0.854 | 0.902 | 0.862 | 0.823 | 0.891 | 0.866 | 0.833 | 0.896 | 0.870 | 0.842 | 0.898 | |
| 36 | 0.880 | 0.851 | 0.906 | 0.880 | 0.853 | 0.909 | 0.861 | 0.828 | 0.892 | 0.870 | 0.837 | 0.904 | 0.875 | 0.843 | 0.907 | |
| 37 | 0.874 | 0.838 | 0.901 | 0.876 | 0.846 | 0.910 | 0.857 | 0.824 | 0.893 | 0.863 | 0.832 | 0.896 | 0.870 | 0.843 | 0.899 | |
| 38 | 0.879 | 0.846 | 0.911 | 0.877 | 0.842 | 0.910 | 0.859 | 0.822 | 0.891 | 0.861 | 0.834 | 0.886 | 0.869 | 0.845 | 0.902 | |
| 39 | 0.879 | 0.853 | 0.908 | 0.881 | 0.852 | 0.908 | 0.857 | 0.816 | 0.901 | 0.858 | 0.825 | 0.890 | 0.865 | 0.830 | 0.901 | |
| 40 | 0.876 | 0.845 | 0.907 | 0.878 | 0.851 | 0.907 | 0.854 | 0.819 | 0.892 | 0.859 | 0.823 | 0.903 | 0.867 | 0.827 | 0.896 | |
| **Total mean** and 95% CI | **0.879** | 0.848 | 0.909 | **0.879** | 0.848 | 0.908 | **0.859** | 0.822 | 0.893 | **0.864** | 0.832 | 0.897 | **0.869** | 0.837 | 0.902 | |
| 8-year AUC | | | | | | | | | | | | | | | |  |
|  | CoxSf | | | CoxEn | | | Feedforward | | | Densenet | | | Tab transformer | | | |
| imp | mean | 95% lci | 95% uci | mean | 95% lci | 95% uci | mean | 95% lci | 95% uci | mean | 95% lci | 95% uci | mean | 95% lci | 95% uci | |
| 1 | 0.893 | 0.873 | 0.911 | 0.891 | 0.872 | 0.909 | 0.871 | 0.843 | 0.898 | 0.874 | 0.854 | 0.895 | 0.882 | 0.861 | 0.901 | |
| 2 | 0.893 | 0.870 | 0.913 | 0.890 | 0.869 | 0.908 | 0.870 | 0.842 | 0.897 | 0.873 | 0.844 | 0.898 | 0.884 | 0.867 | 0.905 | |
| 3 | 0.893 | 0.872 | 0.909 | 0.893 | 0.876 | 0.913 | 0.870 | 0.843 | 0.894 | 0.875 | 0.851 | 0.897 | 0.880 | 0.858 | 0.902 | |
| 4 | 0.893 | 0.874 | 0.913 | 0.891 | 0.869 | 0.908 | 0.869 | 0.840 | 0.891 | 0.874 | 0.845 | 0.901 | 0.885 | 0.861 | 0.905 | |
| 5 | 0.893 | 0.873 | 0.913 | 0.891 | 0.870 | 0.911 | 0.866 | 0.838 | 0.892 | 0.871 | 0.847 | 0.899 | 0.876 | 0.860 | 0.897 | |
| 6 | 0.896 | 0.877 | 0.916 | 0.892 | 0.870 | 0.910 | 0.871 | 0.848 | 0.897 | 0.876 | 0.855 | 0.895 | 0.882 | 0.860 | 0.905 | |
| 7 | 0.892 | 0.871 | 0.914 | 0.889 | 0.866 | 0.909 | 0.870 | 0.846 | 0.896 | 0.874 | 0.848 | 0.903 | 0.884 | 0.863 | 0.903 | |
| 8 | 0.894 | 0.876 | 0.912 | 0.892 | 0.871 | 0.916 | 0.870 | 0.847 | 0.895 | 0.875 | 0.850 | 0.896 | 0.886 | 0.866 | 0.905 | |
| 9 | 0.892 | 0.874 | 0.911 | 0.890 | 0.865 | 0.907 | 0.867 | 0.843 | 0.892 | 0.871 | 0.845 | 0.895 | 0.885 | 0.865 | 0.908 | |
| 10 | 0.893 | 0.873 | 0.914 | 0.892 | 0.873 | 0.909 | 0.871 | 0.847 | 0.892 | 0.873 | 0.849 | 0.900 | 0.887 | 0.868 | 0.911 | |
| 11 | 0.893 | 0.874 | 0.912 | 0.891 | 0.865 | 0.913 | 0.870 | 0.845 | 0.896 | 0.874 | 0.851 | 0.896 | 0.887 | 0.863 | 0.902 | |
| 12 | 0.894 | 0.875 | 0.914 | 0.891 | 0.869 | 0.913 | 0.869 | 0.846 | 0.893 | 0.873 | 0.845 | 0.896 | 0.882 | 0.860 | 0.902 | |
| 13 | 0.894 | 0.873 | 0.913 | 0.892 | 0.870 | 0.913 | 0.870 | 0.845 | 0.893 | 0.875 | 0.852 | 0.897 | 0.888 | 0.869 | 0.905 | |
| 14 | 0.895 | 0.874 | 0.912 | 0.891 | 0.872 | 0.913 | 0.871 | 0.845 | 0.891 | 0.877 | 0.847 | 0.901 | 0.883 | 0.860 | 0.900 | |
| 15 | 0.894 | 0.873 | 0.915 | 0.893 | 0.871 | 0.908 | 0.870 | 0.844 | 0.893 | 0.874 | 0.852 | 0.898 | 0.883 | 0.864 | 0.903 | |
| 16 | 0.895 | 0.874 | 0.920 | 0.891 | 0.873 | 0.908 | 0.873 | 0.851 | 0.897 | 0.878 | 0.857 | 0.898 | 0.885 | 0.866 | 0.904 | |
| 17 | 0.896 | 0.878 | 0.916 | 0.893 | 0.872 | 0.915 | 0.871 | 0.847 | 0.893 | 0.875 | 0.848 | 0.904 | 0.885 | 0.864 | 0.903 | |
| 18 | 0.896 | 0.875 | 0.915 | 0.897 | 0.881 | 0.916 | 0.871 | 0.845 | 0.890 | 0.876 | 0.854 | 0.896 | 0.885 | 0.866 | 0.905 | |
| 19 | 0.896 | 0.875 | 0.918 | 0.894 | 0.873 | 0.916 | 0.870 | 0.844 | 0.894 | 0.874 | 0.846 | 0.895 | 0.886 | 0.861 | 0.908 | |
| 20 | 0.896 | 0.878 | 0.913 | 0.892 | 0.870 | 0.914 | 0.871 | 0.846 | 0.896 | 0.872 | 0.844 | 0.898 | 0.885 | 0.862 | 0.906 | |
| 21 | 0.894 | 0.877 | 0.911 | 0.894 | 0.873 | 0.914 | 0.873 | 0.849 | 0.898 | 0.876 | 0.854 | 0.896 | 0.885 | 0.863 | 0.906 | |
| 22 | 0.894 | 0.872 | 0.912 | 0.891 | 0.870 | 0.911 | 0.871 | 0.847 | 0.898 | 0.874 | 0.845 | 0.898 | 0.883 | 0.860 | 0.906 | |
| 23 | 0.894 | 0.875 | 0.915 | 0.893 | 0.877 | 0.910 | 0.871 | 0.846 | 0.892 | 0.877 | 0.850 | 0.899 | 0.885 | 0.861 | 0.901 | |
| 24 | 0.894 | 0.878 | 0.912 | 0.895 | 0.877 | 0.913 | 0.869 | 0.843 | 0.892 | 0.875 | 0.846 | 0.899 | 0.882 | 0.859 | 0.906 | |
| 25 | 0.894 | 0.867 | 0.915 | 0.895 | 0.879 | 0.914 | 0.869 | 0.843 | 0.893 | 0.876 | 0.850 | 0.904 | 0.887 | 0.859 | 0.905 | |
| 26 | 0.896 | 0.877 | 0.914 | 0.893 | 0.870 | 0.915 | 0.871 | 0.847 | 0.895 | 0.877 | 0.854 | 0.899 | 0.886 | 0.859 | 0.908 | |
| 27 | 0.894 | 0.875 | 0.913 | 0.891 | 0.873 | 0.912 | 0.871 | 0.846 | 0.899 | 0.875 | 0.847 | 0.903 | 0.886 | 0.856 | 0.906 | |
| 28 | 0.894 | 0.874 | 0.912 | 0.892 | 0.867 | 0.917 | 0.874 | 0.848 | 0.896 | 0.877 | 0.855 | 0.898 | 0.886 | 0.868 | 0.903 | |
| 29 | 0.894 | 0.871 | 0.910 | 0.894 | 0.870 | 0.917 | 0.869 | 0.841 | 0.892 | 0.872 | 0.848 | 0.894 | 0.883 | 0.863 | 0.904 | |
| 30 | 0.892 | 0.870 | 0.912 | 0.891 | 0.870 | 0.910 | 0.868 | 0.844 | 0.888 | 0.871 | 0.844 | 0.896 | 0.882 | 0.860 | 0.905 | |
| 31 | 0.892 | 0.871 | 0.917 | 0.890 | 0.875 | 0.908 | 0.870 | 0.844 | 0.899 | 0.874 | 0.852 | 0.898 | 0.880 | 0.860 | 0.906 | |
| 32 | 0.897 | 0.878 | 0.914 | 0.893 | 0.873 | 0.911 | 0.871 | 0.847 | 0.895 | 0.873 | 0.844 | 0.896 | 0.884 | 0.864 | 0.904 | |
| 33 | 0.893 | 0.871 | 0.912 | 0.892 | 0.874 | 0.909 | 0.871 | 0.847 | 0.892 | 0.878 | 0.854 | 0.899 | 0.887 | 0.864 | 0.906 | |
| 34 | 0.893 | 0.875 | 0.912 | 0.892 | 0.870 | 0.910 | 0.871 | 0.846 | 0.894 | 0.877 | 0.849 | 0.901 | 0.884 | 0.867 | 0.910 | |
| 35 | 0.893 | 0.870 | 0.911 | 0.892 | 0.871 | 0.912 | 0.870 | 0.847 | 0.892 | 0.874 | 0.849 | 0.900 | 0.887 | 0.867 | 0.910 | |
| 36 | 0.894 | 0.871 | 0.914 | 0.892 | 0.875 | 0.909 | 0.871 | 0.850 | 0.898 | 0.877 | 0.856 | 0.897 | 0.889 | 0.868 | 0.906 | |
| 37 | 0.893 | 0.873 | 0.912 | 0.889 | 0.868 | 0.906 | 0.867 | 0.843 | 0.893 | 0.873 | 0.848 | 0.902 | 0.882 | 0.859 | 0.904 | |
| 38 | 0.893 | 0.870 | 0.914 | 0.891 | 0.871 | 0.913 | 0.869 | 0.839 | 0.895 | 0.873 | 0.847 | 0.896 | 0.881 | 0.858 | 0.904 | |
| 39 | 0.897 | 0.877 | 0.911 | 0.893 | 0.869 | 0.913 | 0.870 | 0.846 | 0.894 | 0.874 | 0.848 | 0.898 | 0.883 | 0.862 | 0.904 | |
| 40 | 0.892 | 0.872 | 0.912 | 0.891 | 0.871 | 0.908 | 0.868 | 0.844 | 0.891 | 0.869 | 0.846 | 0.893 | 0.882 | 0.861 | 0.907 | |
| **Total mean** and 95% CI | **0.894** | 0.874 | 0.913 | **0.892** | 0.872 | 0.912 | **0.870** | 0.845 | 0.894 | **0.874** | 0.849 | 0.898 | **0.884** | 0.863 | 0.905 | |
| 10-year AUC | | | | | | | | | | | | | | | |  |
|  | CoxSf | | | CoxEn | | | Feedforward | | | Densenet | | | Tab transformer | | | |
| imp | mean | 95% lci | 95% uci | mean | 95% lci | 95% uci | mean | 95% lci | 95% uci | mean | 95% lci | 95% uci | mean | 95% lci | 95% uci | |
| 1 | 0.876 | 0.855 | 0.895 | 0.871 | 0.845 | 0.890 | 0.851 | 0.824 | 0.877 | 0.853 | 0.830 | 0.875 | 0.874 | 0.851 | 0.890 | |
| 2 | 0.880 | 0.864 | 0.897 | 0.872 | 0.853 | 0.890 | 0.852 | 0.829 | 0.875 | 0.855 | 0.828 | 0.874 | 0.872 | 0.853 | 0.892 | |
| 3 | 0.875 | 0.847 | 0.897 | 0.871 | 0.849 | 0.891 | 0.846 | 0.813 | 0.869 | 0.851 | 0.827 | 0.876 | 0.869 | 0.842 | 0.890 | |
| 4 | 0.877 | 0.856 | 0.899 | 0.872 | 0.856 | 0.892 | 0.849 | 0.822 | 0.873 | 0.854 | 0.828 | 0.880 | 0.871 | 0.851 | 0.893 | |
| 5 | 0.877 | 0.860 | 0.897 | 0.870 | 0.849 | 0.889 | 0.847 | 0.820 | 0.875 | 0.853 | 0.824 | 0.876 | 0.861 | 0.839 | 0.886 | |
| 6 | 0.877 | 0.853 | 0.897 | 0.871 | 0.850 | 0.893 | 0.850 | 0.827 | 0.873 | 0.855 | 0.829 | 0.874 | 0.869 | 0.846 | 0.887 | |
| 7 | 0.874 | 0.851 | 0.896 | 0.868 | 0.845 | 0.887 | 0.850 | 0.822 | 0.879 | 0.853 | 0.827 | 0.881 | 0.871 | 0.855 | 0.895 | |
| 8 | 0.876 | 0.856 | 0.897 | 0.870 | 0.853 | 0.894 | 0.851 | 0.827 | 0.876 | 0.855 | 0.828 | 0.877 | 0.870 | 0.850 | 0.889 | |
| 9 | 0.876 | 0.853 | 0.900 | 0.873 | 0.850 | 0.897 | 0.848 | 0.817 | 0.872 | 0.854 | 0.831 | 0.883 | 0.874 | 0.853 | 0.894 | |
| 10 | 0.874 | 0.856 | 0.895 | 0.871 | 0.847 | 0.891 | 0.851 | 0.826 | 0.874 | 0.853 | 0.830 | 0.874 | 0.870 | 0.849 | 0.895 | |
| 11 | 0.874 | 0.852 | 0.897 | 0.871 | 0.849 | 0.896 | 0.848 | 0.820 | 0.876 | 0.851 | 0.827 | 0.877 | 0.870 | 0.849 | 0.887 | |
| 12 | 0.874 | 0.851 | 0.894 | 0.870 | 0.843 | 0.895 | 0.852 | 0.826 | 0.875 | 0.854 | 0.828 | 0.877 | 0.871 | 0.849 | 0.892 | |
| 13 | 0.876 | 0.854 | 0.898 | 0.873 | 0.855 | 0.891 | 0.848 | 0.818 | 0.873 | 0.852 | 0.828 | 0.876 | 0.872 | 0.850 | 0.891 | |
| 14 | 0.877 | 0.858 | 0.898 | 0.871 | 0.851 | 0.887 | 0.851 | 0.829 | 0.875 | 0.857 | 0.830 | 0.881 | 0.870 | 0.854 | 0.889 | |
| 15 | 0.874 | 0.856 | 0.894 | 0.872 | 0.850 | 0.893 | 0.850 | 0.824 | 0.874 | 0.854 | 0.828 | 0.876 | 0.868 | 0.845 | 0.891 | |
| 16 | 0.877 | 0.852 | 0.898 | 0.872 | 0.850 | 0.889 | 0.851 | 0.825 | 0.876 | 0.856 | 0.831 | 0.877 | 0.872 | 0.850 | 0.892 | |
| 17 | 0.876 | 0.855 | 0.898 | 0.872 | 0.852 | 0.893 | 0.850 | 0.829 | 0.873 | 0.855 | 0.827 | 0.882 | 0.867 | 0.845 | 0.888 | |
| 18 | 0.878 | 0.855 | 0.898 | 0.876 | 0.857 | 0.894 | 0.852 | 0.826 | 0.879 | 0.856 | 0.829 | 0.877 | 0.870 | 0.852 | 0.889 | |
| 19 | 0.876 | 0.854 | 0.895 | 0.873 | 0.854 | 0.889 | 0.850 | 0.822 | 0.876 | 0.854 | 0.830 | 0.885 | 0.874 | 0.855 | 0.895 | |
| 20 | 0.877 | 0.853 | 0.898 | 0.872 | 0.854 | 0.889 | 0.853 | 0.831 | 0.875 | 0.852 | 0.825 | 0.876 | 0.869 | 0.847 | 0.891 | |
| 21 | 0.876 | 0.849 | 0.897 | 0.872 | 0.851 | 0.896 | 0.850 | 0.823 | 0.876 | 0.852 | 0.827 | 0.878 | 0.868 | 0.850 | 0.893 | |
| 22 | 0.876 | 0.854 | 0.893 | 0.871 | 0.853 | 0.893 | 0.855 | 0.833 | 0.880 | 0.857 | 0.828 | 0.878 | 0.872 | 0.854 | 0.897 | |
| 23 | 0.875 | 0.856 | 0.900 | 0.871 | 0.852 | 0.893 | 0.847 | 0.816 | 0.875 | 0.852 | 0.828 | 0.876 | 0.869 | 0.852 | 0.889 | |
| 24 | 0.877 | 0.860 | 0.896 | 0.873 | 0.854 | 0.895 | 0.851 | 0.827 | 0.875 | 0.857 | 0.832 | 0.881 | 0.873 | 0.850 | 0.895 | |
| 25 | 0.875 | 0.854 | 0.891 | 0.871 | 0.849 | 0.893 | 0.850 | 0.824 | 0.877 | 0.855 | 0.828 | 0.879 | 0.872 | 0.848 | 0.890 | |
| 26 | 0.876 | 0.854 | 0.897 | 0.873 | 0.847 | 0.896 | 0.853 | 0.826 | 0.878 | 0.857 | 0.832 | 0.877 | 0.874 | 0.854 | 0.896 | |
| 27 | 0.876 | 0.852 | 0.895 | 0.871 | 0.849 | 0.897 | 0.852 | 0.829 | 0.876 | 0.853 | 0.825 | 0.881 | 0.872 | 0.843 | 0.892 | |
| 28 | 0.878 | 0.858 | 0.897 | 0.871 | 0.851 | 0.891 | 0.849 | 0.817 | 0.874 | 0.856 | 0.830 | 0.877 | 0.876 | 0.856 | 0.894 | |
| 29 | 0.875 | 0.856 | 0.900 | 0.871 | 0.849 | 0.894 | 0.848 | 0.820 | 0.873 | 0.854 | 0.829 | 0.881 | 0.872 | 0.850 | 0.891 | |
| 30 | 0.875 | 0.854 | 0.897 | 0.871 | 0.849 | 0.892 | 0.850 | 0.824 | 0.873 | 0.853 | 0.830 | 0.876 | 0.870 | 0.849 | 0.896 | |
| 31 | 0.875 | 0.856 | 0.897 | 0.869 | 0.842 | 0.890 | 0.851 | 0.825 | 0.874 | 0.855 | 0.831 | 0.878 | 0.872 | 0.851 | 0.892 | |
| 32 | 0.877 | 0.856 | 0.901 | 0.872 | 0.852 | 0.896 | 0.853 | 0.830 | 0.876 | 0.854 | 0.826 | 0.875 | 0.874 | 0.853 | 0.894 | |
| 33 | 0.875 | 0.853 | 0.895 | 0.871 | 0.846 | 0.894 | 0.847 | 0.817 | 0.873 | 0.854 | 0.829 | 0.877 | 0.873 | 0.851 | 0.897 | |
| 34 | 0.876 | 0.862 | 0.895 | 0.871 | 0.845 | 0.892 | 0.849 | 0.821 | 0.873 | 0.855 | 0.829 | 0.881 | 0.871 | 0.850 | 0.893 | |
| 35 | 0.874 | 0.850 | 0.895 | 0.871 | 0.847 | 0.892 | 0.850 | 0.824 | 0.875 | 0.855 | 0.828 | 0.881 | 0.872 | 0.851 | 0.890 | |
| 36 | 0.875 | 0.851 | 0.897 | 0.874 | 0.849 | 0.896 | 0.851 | 0.829 | 0.876 | 0.856 | 0.835 | 0.874 | 0.873 | 0.851 | 0.896 | |
| 37 | 0.874 | 0.852 | 0.892 | 0.870 | 0.849 | 0.889 | 0.847 | 0.826 | 0.871 | 0.854 | 0.828 | 0.880 | 0.871 | 0.852 | 0.891 | |
| 38 | 0.876 | 0.848 | 0.898 | 0.870 | 0.851 | 0.891 | 0.850 | 0.819 | 0.874 | 0.854 | 0.827 | 0.876 | 0.870 | 0.849 | 0.892 | |
| 39 | 0.876 | 0.854 | 0.896 | 0.871 | 0.851 | 0.889 | 0.852 | 0.829 | 0.877 | 0.854 | 0.832 | 0.883 | 0.872 | 0.852 | 0.891 | |
| 40 | 0.872 | 0.852 | 0.891 | 0.868 | 0.846 | 0.889 | 0.846 | 0.816 | 0.875 | 0.850 | 0.824 | 0.872 | 0.869 | 0.851 | 0.886 | |
| **Total mean** and 95% CI | **0.876** | 0.854 | 0.896 | **0.871** | 0.850 | 0.892 | **0.850** | 0.824 | 0.875 | **0.854** | 0.829 | 0.878 | **0.871** | 0.850 | 0.892 | |
| 12-year AUC | | | | | | | | | | | | | | | |  |
|  | CoxSf | | | CoxEn | | | Feedforward | | | Densenet | | | Tab transformer | | | |
| imp | mean | 95% lci | 95% uci | mean | 95% lci | 95% uci | mean | 95% lci | 95% uci | mean | 95% lci | 95% uci | mean | 95% lci | 95% uci | |
| 1 | 0.786 | 0.763 | 0.812 | 0.779 | 0.750 | 0.809 | 0.768 | 0.737 | 0.795 | 0.769 | 0.737 | 0.797 | 0.794 | 0.765 | 0.825 | |
| 2 | 0.789 | 0.752 | 0.817 | 0.781 | 0.757 | 0.805 | 0.768 | 0.732 | 0.802 | 0.772 | 0.742 | 0.796 | 0.792 | 0.768 | 0.815 | |
| 3 | 0.784 | 0.760 | 0.809 | 0.783 | 0.758 | 0.810 | 0.762 | 0.734 | 0.790 | 0.769 | 0.737 | 0.801 | 0.794 | 0.764 | 0.818 | |
| 4 | 0.785 | 0.759 | 0.810 | 0.785 | 0.756 | 0.811 | 0.763 | 0.731 | 0.798 | 0.767 | 0.738 | 0.798 | 0.794 | 0.767 | 0.820 | |
| 5 | 0.789 | 0.761 | 0.815 | 0.783 | 0.760 | 0.813 | 0.766 | 0.738 | 0.796 | 0.770 | 0.739 | 0.796 | 0.789 | 0.761 | 0.813 | |
| 6 | 0.789 | 0.765 | 0.814 | 0.786 | 0.756 | 0.811 | 0.767 | 0.738 | 0.797 | 0.773 | 0.752 | 0.806 | 0.792 | 0.759 | 0.821 | |
| 7 | 0.787 | 0.757 | 0.814 | 0.782 | 0.756 | 0.812 | 0.766 | 0.735 | 0.793 | 0.768 | 0.734 | 0.797 | 0.792 | 0.764 | 0.823 | |
| 8 | 0.785 | 0.749 | 0.814 | 0.781 | 0.754 | 0.813 | 0.769 | 0.732 | 0.803 | 0.771 | 0.736 | 0.798 | 0.797 | 0.770 | 0.823 | |
| 9 | 0.786 | 0.755 | 0.809 | 0.783 | 0.756 | 0.812 | 0.761 | 0.734 | 0.788 | 0.772 | 0.737 | 0.801 | 0.796 | 0.774 | 0.818 | |
| 10 | 0.786 | 0.759 | 0.810 | 0.782 | 0.756 | 0.808 | 0.764 | 0.733 | 0.797 | 0.768 | 0.742 | 0.797 | 0.797 | 0.769 | 0.822 | |
| 11 | 0.786 | 0.759 | 0.812 | 0.785 | 0.758 | 0.814 | 0.765 | 0.733 | 0.791 | 0.767 | 0.733 | 0.796 | 0.795 | 0.770 | 0.824 | |
| 12 | 0.783 | 0.759 | 0.806 | 0.781 | 0.754 | 0.810 | 0.768 | 0.735 | 0.802 | 0.772 | 0.739 | 0.799 | 0.797 | 0.774 | 0.827 | |
| 13 | 0.788 | 0.757 | 0.814 | 0.784 | 0.753 | 0.811 | 0.762 | 0.735 | 0.792 | 0.768 | 0.738 | 0.799 | 0.794 | 0.766 | 0.821 | |
| 14 | 0.788 | 0.759 | 0.813 | 0.782 | 0.758 | 0.804 | 0.767 | 0.735 | 0.802 | 0.770 | 0.741 | 0.799 | 0.796 | 0.763 | 0.819 | |
| 15 | 0.784 | 0.761 | 0.809 | 0.783 | 0.752 | 0.812 | 0.768 | 0.738 | 0.799 | 0.769 | 0.738 | 0.794 | 0.790 | 0.767 | 0.817 | |
| 16 | 0.788 | 0.762 | 0.817 | 0.783 | 0.757 | 0.810 | 0.767 | 0.738 | 0.794 | 0.771 | 0.750 | 0.802 | 0.795 | 0.770 | 0.824 | |
| 17 | 0.787 | 0.758 | 0.811 | 0.783 | 0.749 | 0.812 | 0.765 | 0.736 | 0.796 | 0.770 | 0.736 | 0.801 | 0.791 | 0.766 | 0.822 | |
| 18 | 0.783 | 0.754 | 0.811 | 0.784 | 0.754 | 0.815 | 0.767 | 0.730 | 0.797 | 0.771 | 0.739 | 0.799 | 0.792 | 0.768 | 0.831 | |
| 19 | 0.789 | 0.762 | 0.816 | 0.785 | 0.760 | 0.812 | 0.768 | 0.737 | 0.794 | 0.774 | 0.734 | 0.805 | 0.796 | 0.771 | 0.827 | |
| 20 | 0.787 | 0.758 | 0.816 | 0.782 | 0.751 | 0.810 | 0.768 | 0.734 | 0.804 | 0.768 | 0.746 | 0.796 | 0.793 | 0.763 | 0.821 | |
| 21 | 0.787 | 0.756 | 0.812 | 0.783 | 0.755 | 0.812 | 0.768 | 0.736 | 0.794 | 0.770 | 0.736 | 0.797 | 0.793 | 0.765 | 0.819 | |
| 22 | 0.788 | 0.755 | 0.813 | 0.785 | 0.761 | 0.811 | 0.772 | 0.736 | 0.807 | 0.775 | 0.747 | 0.801 | 0.797 | 0.772 | 0.831 | |
| 23 | 0.785 | 0.761 | 0.810 | 0.783 | 0.757 | 0.811 | 0.760 | 0.733 | 0.784 | 0.767 | 0.734 | 0.797 | 0.789 | 0.761 | 0.818 | |
| 24 | 0.787 | 0.762 | 0.813 | 0.784 | 0.764 | 0.810 | 0.767 | 0.736 | 0.799 | 0.771 | 0.744 | 0.800 | 0.796 | 0.770 | 0.826 | |
| 25 | 0.786 | 0.752 | 0.811 | 0.780 | 0.750 | 0.804 | 0.765 | 0.739 | 0.795 | 0.770 | 0.739 | 0.795 | 0.790 | 0.762 | 0.808 | |
| 26 | 0.785 | 0.752 | 0.812 | 0.782 | 0.749 | 0.809 | 0.768 | 0.737 | 0.793 | 0.773 | 0.751 | 0.804 | 0.802 | 0.771 | 0.825 | |
| 27 | 0.787 | 0.759 | 0.814 | 0.785 | 0.757 | 0.812 | 0.768 | 0.730 | 0.803 | 0.768 | 0.730 | 0.799 | 0.794 | 0.762 | 0.816 | |
| 28 | 0.788 | 0.762 | 0.816 | 0.786 | 0.762 | 0.809 | 0.764 | 0.736 | 0.791 | 0.774 | 0.740 | 0.802 | 0.795 | 0.763 | 0.823 | |
| 29 | 0.785 | 0.759 | 0.815 | 0.781 | 0.752 | 0.806 | 0.762 | 0.729 | 0.794 | 0.772 | 0.735 | 0.805 | 0.797 | 0.774 | 0.825 | |
| 30 | 0.785 | 0.760 | 0.806 | 0.783 | 0.752 | 0.811 | 0.768 | 0.737 | 0.799 | 0.768 | 0.744 | 0.798 | 0.794 | 0.764 | 0.818 | |
| 31 | 0.785 | 0.756 | 0.812 | 0.782 | 0.752 | 0.812 | 0.770 | 0.739 | 0.793 | 0.772 | 0.741 | 0.800 | 0.794 | 0.774 | 0.823 | |
| 32 | 0.786 | 0.762 | 0.809 | 0.782 | 0.750 | 0.807 | 0.770 | 0.737 | 0.803 | 0.773 | 0.741 | 0.797 | 0.793 | 0.768 | 0.820 | |
| 33 | 0.785 | 0.757 | 0.812 | 0.783 | 0.758 | 0.810 | 0.761 | 0.734 | 0.786 | 0.768 | 0.739 | 0.799 | 0.794 | 0.767 | 0.824 | |
| 34 | 0.786 | 0.759 | 0.815 | 0.784 | 0.761 | 0.805 | 0.763 | 0.730 | 0.799 | 0.767 | 0.738 | 0.799 | 0.793 | 0.767 | 0.814 | |
| 35 | 0.784 | 0.758 | 0.809 | 0.781 | 0.756 | 0.804 | 0.768 | 0.738 | 0.800 | 0.770 | 0.737 | 0.796 | 0.790 | 0.765 | 0.814 | |
| 36 | 0.787 | 0.761 | 0.812 | 0.784 | 0.755 | 0.811 | 0.769 | 0.740 | 0.798 | 0.773 | 0.750 | 0.804 | 0.797 | 0.771 | 0.822 | |
| 37 | 0.784 | 0.759 | 0.812 | 0.780 | 0.754 | 0.807 | 0.763 | 0.733 | 0.794 | 0.770 | 0.736 | 0.801 | 0.794 | 0.768 | 0.822 | |
| 38 | 0.787 | 0.761 | 0.811 | 0.785 | 0.760 | 0.820 | 0.766 | 0.736 | 0.792 | 0.771 | 0.738 | 0.800 | 0.789 | 0.766 | 0.819 | |
| 39 | 0.786 | 0.758 | 0.810 | 0.782 | 0.756 | 0.804 | 0.770 | 0.738 | 0.801 | 0.774 | 0.735 | 0.804 | 0.794 | 0.769 | 0.825 | |
| 40 | 0.785 | 0.757 | 0.814 | 0.782 | 0.757 | 0.810 | 0.760 | 0.733 | 0.786 | 0.766 | 0.741 | 0.795 | 0.791 | 0.768 | 0.815 | |
| **Total mean** and 95% CI | **0.786** | 0.758 | 0.812 | **0.783** | 0.756 | 0.810 | **0.766** | 0.735 | 0.796 | **0.770** | 0.740 | 0.799 | **0.794** | 0.767 | 0.821 | |

Abbreviations: CoxSf: Cox models with selected features; CoxEn: Cox models with Elastic Net regularization; FeedForward: Feedforward neural network; Densenet: Densely Connected Convolutional Network; TabTransformer: TabTransformer neural network; imp: imputation number; LCI: 95% lower confidence interval; UCI: 95% upper confidence interval; 95% CI: 95% confidence intervals

## Supplementary Table 5. Time-dependent balanced accuracy mean and 95% confidence intervals

| 4-year balanced accuracy | | | | | | | | | | | | | | | |
| --- | --- | --- | --- | --- | --- | --- | --- | --- | --- | --- | --- | --- | --- | --- | --- |
|  | CoxSf | | | CoxEn | | | Feedforward | | | Densenet | | | Tab transformer | | |
| imp | mean | 95% lci | 95% uci | mean | 95% lci | 95% uci | mean | 95% lci | 95% uci | mean | 95% lci | 95% uci | mean | 95% lci | 95% uci |
| 1 | 0.834 | 0.777 | 0.892 | 0.829 | 0.772 | 0.888 | 0.823 | 0.769 | 0.876 | 0.816 | 0.772 | 0.869 | 0.829 | 0.778 | 0.875 |
| 2 | 0.826 | 0.770 | 0.889 | 0.826 | 0.768 | 0.885 | 0.817 | 0.755 | 0.891 | 0.810 | 0.762 | 0.869 | 0.831 | 0.768 | 0.881 |
| 3 | 0.832 | 0.776 | 0.884 | 0.829 | 0.769 | 0.896 | 0.813 | 0.752 | 0.871 | 0.811 | 0.753 | 0.861 | 0.829 | 0.780 | 0.878 |
| 4 | 0.841 | 0.800 | 0.885 | 0.831 | 0.782 | 0.893 | 0.821 | 0.774 | 0.875 | 0.820 | 0.772 | 0.877 | 0.844 | 0.790 | 0.893 |
| 5 | 0.834 | 0.779 | 0.883 | 0.827 | 0.783 | 0.888 | 0.822 | 0.765 | 0.886 | 0.814 | 0.764 | 0.858 | 0.834 | 0.776 | 0.893 |
| 6 | 0.832 | 0.775 | 0.894 | 0.824 | 0.769 | 0.870 | 0.817 | 0.761 | 0.868 | 0.819 | 0.767 | 0.874 | 0.840 | 0.786 | 0.887 |
| 7 | 0.838 | 0.786 | 0.890 | 0.830 | 0.778 | 0.891 | 0.823 | 0.768 | 0.888 | 0.826 | 0.782 | 0.888 | 0.834 | 0.784 | 0.892 |
| 8 | 0.841 | 0.793 | 0.897 | 0.823 | 0.763 | 0.890 | 0.815 | 0.750 | 0.879 | 0.820 | 0.773 | 0.868 | 0.838 | 0.790 | 0.881 |
| 9 | 0.832 | 0.775 | 0.886 | 0.827 | 0.776 | 0.876 | 0.816 | 0.750 | 0.872 | 0.809 | 0.752 | 0.863 | 0.835 | 0.778 | 0.885 |
| 10 | 0.832 | 0.778 | 0.890 | 0.820 | 0.762 | 0.868 | 0.819 | 0.764 | 0.872 | 0.816 | 0.749 | 0.891 | 0.843 | 0.791 | 0.897 |
| 11 | 0.838 | 0.785 | 0.892 | 0.829 | 0.766 | 0.882 | 0.820 | 0.767 | 0.873 | 0.816 | 0.755 | 0.872 | 0.835 | 0.786 | 0.895 |
| 12 | 0.827 | 0.764 | 0.888 | 0.830 | 0.777 | 0.875 | 0.813 | 0.753 | 0.871 | 0.807 | 0.754 | 0.863 | 0.827 | 0.769 | 0.876 |
| 13 | 0.833 | 0.780 | 0.890 | 0.828 | 0.767 | 0.878 | 0.819 | 0.753 | 0.877 | 0.817 | 0.754 | 0.878 | 0.835 | 0.783 | 0.886 |
| 14 | 0.832 | 0.783 | 0.893 | 0.833 | 0.779 | 0.881 | 0.820 | 0.772 | 0.882 | 0.816 | 0.768 | 0.865 | 0.830 | 0.767 | 0.878 |
| 15 | 0.833 | 0.780 | 0.889 | 0.832 | 0.783 | 0.883 | 0.824 | 0.762 | 0.883 | 0.819 | 0.771 | 0.865 | 0.834 | 0.770 | 0.889 |
| 16 | 0.833 | 0.783 | 0.891 | 0.827 | 0.771 | 0.877 | 0.816 | 0.757 | 0.873 | 0.819 | 0.772 | 0.885 | 0.834 | 0.776 | 0.891 |
| 17 | 0.829 | 0.764 | 0.881 | 0.817 | 0.766 | 0.865 | 0.815 | 0.757 | 0.871 | 0.813 | 0.757 | 0.870 | 0.832 | 0.770 | 0.894 |
| 18 | 0.832 | 0.783 | 0.877 | 0.824 | 0.773 | 0.885 | 0.821 | 0.769 | 0.870 | 0.818 | 0.767 | 0.867 | 0.821 | 0.766 | 0.898 |
| 19 | 0.833 | 0.772 | 0.894 | 0.835 | 0.781 | 0.893 | 0.821 | 0.758 | 0.877 | 0.813 | 0.752 | 0.867 | 0.838 | 0.776 | 0.895 |
| 20 | 0.838 | 0.790 | 0.894 | 0.831 | 0.770 | 0.893 | 0.818 | 0.750 | 0.891 | 0.816 | 0.764 | 0.899 | 0.837 | 0.779 | 0.887 |
| 21 | 0.829 | 0.775 | 0.876 | 0.825 | 0.779 | 0.893 | 0.819 | 0.768 | 0.876 | 0.813 | 0.760 | 0.871 | 0.842 | 0.786 | 0.877 |
| 22 | 0.833 | 0.767 | 0.894 | 0.833 | 0.776 | 0.888 | 0.819 | 0.761 | 0.883 | 0.820 | 0.769 | 0.883 | 0.836 | 0.783 | 0.905 |
| 23 | 0.840 | 0.778 | 0.901 | 0.833 | 0.785 | 0.876 | 0.820 | 0.757 | 0.874 | 0.819 | 0.763 | 0.876 | 0.838 | 0.786 | 0.898 |
| 24 | 0.837 | 0.777 | 0.892 | 0.831 | 0.784 | 0.900 | 0.822 | 0.764 | 0.879 | 0.817 | 0.770 | 0.868 | 0.820 | 0.767 | 0.885 |
| 25 | 0.842 | 0.797 | 0.886 | 0.834 | 0.787 | 0.903 | 0.828 | 0.760 | 0.891 | 0.825 | 0.766 | 0.869 | 0.845 | 0.786 | 0.901 |
| 26 | 0.830 | 0.783 | 0.884 | 0.828 | 0.762 | 0.886 | 0.817 | 0.760 | 0.873 | 0.819 | 0.771 | 0.879 | 0.833 | 0.774 | 0.891 |
| 27 | 0.829 | 0.773 | 0.883 | 0.823 | 0.766 | 0.877 | 0.814 | 0.753 | 0.884 | 0.815 | 0.771 | 0.874 | 0.822 | 0.767 | 0.875 |
| 28 | 0.833 | 0.780 | 0.893 | 0.826 | 0.771 | 0.886 | 0.825 | 0.763 | 0.883 | 0.830 | 0.777 | 0.883 | 0.836 | 0.798 | 0.892 |
| 29 | 0.831 | 0.781 | 0.888 | 0.827 | 0.776 | 0.883 | 0.820 | 0.773 | 0.869 | 0.809 | 0.753 | 0.870 | 0.827 | 0.773 | 0.890 |
| 30 | 0.832 | 0.760 | 0.889 | 0.825 | 0.779 | 0.871 | 0.819 | 0.760 | 0.872 | 0.814 | 0.752 | 0.891 | 0.839 | 0.789 | 0.883 |
| 31 | 0.827 | 0.783 | 0.887 | 0.829 | 0.776 | 0.878 | 0.823 | 0.773 | 0.879 | 0.821 | 0.767 | 0.880 | 0.838 | 0.775 | 0.901 |
| 32 | 0.833 | 0.784 | 0.877 | 0.833 | 0.774 | 0.897 | 0.818 | 0.755 | 0.885 | 0.811 | 0.760 | 0.873 | 0.843 | 0.790 | 0.895 |
| 33 | 0.832 | 0.777 | 0.883 | 0.829 | 0.774 | 0.890 | 0.819 | 0.759 | 0.877 | 0.813 | 0.751 | 0.864 | 0.836 | 0.775 | 0.895 |
| 34 | 0.833 | 0.786 | 0.889 | 0.829 | 0.779 | 0.875 | 0.819 | 0.770 | 0.874 | 0.811 | 0.761 | 0.859 | 0.826 | 0.775 | 0.890 |
| 35 | 0.834 | 0.784 | 0.883 | 0.827 | 0.783 | 0.883 | 0.825 | 0.763 | 0.883 | 0.815 | 0.766 | 0.857 | 0.821 | 0.777 | 0.875 |
| 36 | 0.843 | 0.800 | 0.901 | 0.832 | 0.786 | 0.883 | 0.821 | 0.764 | 0.872 | 0.817 | 0.759 | 0.874 | 0.842 | 0.786 | 0.902 |
| 37 | 0.828 | 0.781 | 0.882 | 0.824 | 0.766 | 0.886 | 0.819 | 0.767 | 0.876 | 0.813 | 0.755 | 0.869 | 0.830 | 0.778 | 0.874 |
| 38 | 0.831 | 0.784 | 0.885 | 0.824 | 0.766 | 0.883 | 0.813 | 0.748 | 0.870 | 0.810 | 0.760 | 0.860 | 0.825 | 0.776 | 0.877 |
| 39 | 0.830 | 0.778 | 0.893 | 0.828 | 0.776 | 0.887 | 0.813 | 0.753 | 0.872 | 0.807 | 0.744 | 0.865 | 0.832 | 0.788 | 0.889 |
| 40 | 0.834 | 0.786 | 0.878 | 0.828 | 0.785 | 0.878 | 0.817 | 0.753 | 0.872 | 0.815 | 0.756 | 0.894 | 0.836 | 0.767 | 0.905 |
| **Total mean** and 95% CI | **0.833** | 0.780 | 0.888 | **0.828** | 0.775 | 0.884 | **0.819** | 0.761 | 0.877 | **0.816** | 0.762 | 0.873 | **0.834** | 0.779 | 0.889 |
| 6-year balanced accuracy | | | | | | | | | | | | | | | |
|  | CoxSf | | | CoxEn | | | Feedforward | | | Densenet | | | Tab transformer | | |
| imp | mean | 95% lci | 95% uci | mean | 95% lci | 95% uci | mean | 95% lci | 95% uci | mean | 95% lci | 95% uci | mean | 95% lci | 95% uci |
| 1 | 0.819 | 0.774 | 0.853 | 0.819 | 0.779 | 0.857 | 0.804 | 0.762 | 0.840 | 0.802 | 0.770 | 0.836 | 0.814 | 0.780 | 0.842 |
| 2 | 0.819 | 0.785 | 0.857 | 0.816 | 0.788 | 0.848 | 0.798 | 0.752 | 0.841 | 0.799 | 0.762 | 0.840 | 0.806 | 0.771 | 0.842 |
| 3 | 0.819 | 0.785 | 0.847 | 0.819 | 0.782 | 0.854 | 0.795 | 0.759 | 0.835 | 0.799 | 0.765 | 0.836 | 0.807 | 0.771 | 0.848 |
| 4 | 0.824 | 0.793 | 0.855 | 0.821 | 0.781 | 0.861 | 0.800 | 0.764 | 0.835 | 0.805 | 0.773 | 0.845 | 0.818 | 0.782 | 0.853 |
| 5 | 0.819 | 0.785 | 0.854 | 0.812 | 0.772 | 0.850 | 0.799 | 0.757 | 0.839 | 0.798 | 0.764 | 0.837 | 0.811 | 0.778 | 0.861 |
| 6 | 0.817 | 0.789 | 0.850 | 0.814 | 0.780 | 0.851 | 0.799 | 0.767 | 0.836 | 0.803 | 0.771 | 0.840 | 0.808 | 0.764 | 0.849 |
| 7 | 0.818 | 0.788 | 0.858 | 0.815 | 0.776 | 0.854 | 0.800 | 0.759 | 0.832 | 0.802 | 0.769 | 0.844 | 0.817 | 0.788 | 0.857 |
| 8 | 0.819 | 0.790 | 0.849 | 0.815 | 0.779 | 0.853 | 0.799 | 0.755 | 0.840 | 0.802 | 0.769 | 0.834 | 0.810 | 0.778 | 0.840 |
| 9 | 0.817 | 0.791 | 0.852 | 0.816 | 0.781 | 0.853 | 0.797 | 0.756 | 0.837 | 0.798 | 0.764 | 0.837 | 0.815 | 0.773 | 0.850 |
| 10 | 0.819 | 0.783 | 0.862 | 0.814 | 0.778 | 0.860 | 0.801 | 0.766 | 0.834 | 0.801 | 0.761 | 0.853 | 0.819 | 0.790 | 0.856 |
| 11 | 0.819 | 0.783 | 0.853 | 0.815 | 0.772 | 0.851 | 0.799 | 0.758 | 0.838 | 0.799 | 0.762 | 0.834 | 0.817 | 0.775 | 0.851 |
| 12 | 0.814 | 0.788 | 0.843 | 0.817 | 0.779 | 0.852 | 0.797 | 0.750 | 0.838 | 0.798 | 0.760 | 0.840 | 0.811 | 0.776 | 0.841 |
| 13 | 0.819 | 0.789 | 0.853 | 0.817 | 0.785 | 0.854 | 0.797 | 0.760 | 0.837 | 0.799 | 0.765 | 0.835 | 0.820 | 0.784 | 0.853 |
| 14 | 0.820 | 0.785 | 0.849 | 0.818 | 0.779 | 0.851 | 0.799 | 0.771 | 0.839 | 0.803 | 0.769 | 0.848 | 0.807 | 0.772 | 0.845 |
| 15 | 0.819 | 0.783 | 0.859 | 0.819 | 0.787 | 0.859 | 0.804 | 0.766 | 0.842 | 0.805 | 0.767 | 0.839 | 0.808 | 0.770 | 0.851 |
| 16 | 0.817 | 0.777 | 0.855 | 0.813 | 0.781 | 0.847 | 0.797 | 0.764 | 0.835 | 0.802 | 0.761 | 0.842 | 0.818 | 0.783 | 0.858 |
| 17 | 0.818 | 0.778 | 0.854 | 0.812 | 0.777 | 0.843 | 0.796 | 0.764 | 0.842 | 0.796 | 0.764 | 0.846 | 0.815 | 0.778 | 0.851 |
| 18 | 0.823 | 0.784 | 0.861 | 0.816 | 0.776 | 0.847 | 0.799 | 0.758 | 0.839 | 0.802 | 0.767 | 0.839 | 0.810 | 0.775 | 0.848 |
| 19 | 0.822 | 0.789 | 0.856 | 0.821 | 0.782 | 0.853 | 0.800 | 0.764 | 0.839 | 0.797 | 0.761 | 0.840 | 0.816 | 0.781 | 0.856 |
| 20 | 0.820 | 0.781 | 0.858 | 0.817 | 0.783 | 0.856 | 0.799 | 0.762 | 0.839 | 0.796 | 0.758 | 0.846 | 0.813 | 0.778 | 0.848 |
| 21 | 0.813 | 0.781 | 0.846 | 0.815 | 0.779 | 0.858 | 0.801 | 0.762 | 0.840 | 0.799 | 0.759 | 0.833 | 0.813 | 0.778 | 0.849 |
| 22 | 0.816 | 0.783 | 0.847 | 0.816 | 0.781 | 0.850 | 0.797 | 0.755 | 0.841 | 0.801 | 0.761 | 0.848 | 0.808 | 0.775 | 0.845 |
| 23 | 0.822 | 0.784 | 0.858 | 0.818 | 0.783 | 0.858 | 0.800 | 0.762 | 0.844 | 0.801 | 0.768 | 0.839 | 0.815 | 0.774 | 0.849 |
| 24 | 0.825 | 0.797 | 0.858 | 0.824 | 0.795 | 0.863 | 0.803 | 0.762 | 0.832 | 0.806 | 0.774 | 0.843 | 0.806 | 0.771 | 0.848 |
| 25 | 0.818 | 0.786 | 0.854 | 0.819 | 0.780 | 0.858 | 0.801 | 0.755 | 0.840 | 0.801 | 0.761 | 0.835 | 0.817 | 0.787 | 0.856 |
| 26 | 0.821 | 0.790 | 0.848 | 0.816 | 0.787 | 0.847 | 0.800 | 0.754 | 0.841 | 0.807 | 0.769 | 0.849 | 0.817 | 0.761 | 0.853 |
| 27 | 0.816 | 0.785 | 0.848 | 0.813 | 0.779 | 0.850 | 0.798 | 0.754 | 0.837 | 0.799 | 0.768 | 0.845 | 0.812 | 0.771 | 0.848 |
| 28 | 0.816 | 0.785 | 0.848 | 0.817 | 0.783 | 0.851 | 0.801 | 0.765 | 0.840 | 0.805 | 0.770 | 0.839 | 0.815 | 0.780 | 0.853 |
| 29 | 0.818 | 0.787 | 0.851 | 0.818 | 0.782 | 0.852 | 0.797 | 0.763 | 0.831 | 0.795 | 0.758 | 0.834 | 0.803 | 0.770 | 0.840 |
| 30 | 0.819 | 0.786 | 0.851 | 0.812 | 0.775 | 0.844 | 0.799 | 0.754 | 0.838 | 0.795 | 0.752 | 0.843 | 0.812 | 0.785 | 0.852 |
| 31 | 0.816 | 0.778 | 0.849 | 0.816 | 0.781 | 0.853 | 0.800 | 0.764 | 0.833 | 0.804 | 0.771 | 0.838 | 0.811 | 0.777 | 0.860 |
| 32 | 0.818 | 0.786 | 0.855 | 0.818 | 0.781 | 0.860 | 0.799 | 0.751 | 0.843 | 0.799 | 0.759 | 0.839 | 0.816 | 0.780 | 0.852 |
| 33 | 0.817 | 0.789 | 0.852 | 0.817 | 0.783 | 0.856 | 0.800 | 0.763 | 0.838 | 0.802 | 0.768 | 0.837 | 0.814 | 0.772 | 0.851 |
| 34 | 0.820 | 0.785 | 0.855 | 0.815 | 0.780 | 0.849 | 0.802 | 0.764 | 0.839 | 0.804 | 0.770 | 0.844 | 0.813 | 0.777 | 0.846 |
| 35 | 0.819 | 0.789 | 0.850 | 0.816 | 0.785 | 0.855 | 0.803 | 0.763 | 0.841 | 0.803 | 0.770 | 0.842 | 0.804 | 0.778 | 0.846 |
| 36 | 0.818 | 0.787 | 0.862 | 0.820 | 0.783 | 0.855 | 0.800 | 0.760 | 0.837 | 0.806 | 0.766 | 0.846 | 0.826 | 0.790 | 0.860 |
| 37 | 0.813 | 0.779 | 0.843 | 0.815 | 0.779 | 0.854 | 0.798 | 0.760 | 0.837 | 0.798 | 0.765 | 0.844 | 0.819 | 0.781 | 0.854 |
| 38 | 0.820 | 0.783 | 0.859 | 0.815 | 0.781 | 0.850 | 0.797 | 0.765 | 0.834 | 0.798 | 0.761 | 0.835 | 0.820 | 0.781 | 0.845 |
| 39 | 0.820 | 0.786 | 0.852 | 0.818 | 0.786 | 0.849 | 0.798 | 0.751 | 0.846 | 0.798 | 0.759 | 0.838 | 0.810 | 0.773 | 0.846 |
| 40 | 0.816 | 0.780 | 0.859 | 0.815 | 0.787 | 0.852 | 0.795 | 0.758 | 0.837 | 0.795 | 0.756 | 0.838 | 0.811 | 0.772 | 0.849 |
| **Total mean** and 95% CI | **0.819** | 0.785 | 0.853 | **0.817** | 0.781 | 0.853 | **0.799** | 0.760 | 0.838 | **0.801** | 0.765 | 0.840 | **0.813** | 0.777 | 0.850 |
| 8-year balanced accuracy | | | | | | | | | | | | | | | |
|  | CoxSf | | | CoxEn | | | Feedforward | | | Densenet | | | Tab transformer | | |
| imp | mean | 95% lci | 95% uci | mean | 95% lci | 95% uci | mean | 95% lci | 95% uci | mean | 95% lci | 95% uci | mean | 95% lci | 95% uci |
| 1 | 0.826 | 0.801 | 0.852 | 0.823 | 0.797 | 0.850 | 0.801 | 0.768 | 0.834 | 0.804 | 0.777 | 0.834 | 0.811 | 0.784 | 0.831 |
| 2 | 0.828 | 0.804 | 0.856 | 0.822 | 0.797 | 0.851 | 0.799 | 0.771 | 0.828 | 0.803 | 0.774 | 0.834 | 0.813 | 0.791 | 0.834 |
| 3 | 0.827 | 0.800 | 0.852 | 0.824 | 0.800 | 0.849 | 0.801 | 0.772 | 0.827 | 0.805 | 0.780 | 0.830 | 0.810 | 0.785 | 0.837 |
| 4 | 0.829 | 0.806 | 0.859 | 0.825 | 0.795 | 0.852 | 0.801 | 0.777 | 0.825 | 0.805 | 0.769 | 0.835 | 0.813 | 0.791 | 0.837 |
| 5 | 0.828 | 0.808 | 0.856 | 0.823 | 0.802 | 0.849 | 0.795 | 0.766 | 0.827 | 0.800 | 0.771 | 0.830 | 0.806 | 0.786 | 0.828 |
| 6 | 0.828 | 0.801 | 0.855 | 0.822 | 0.795 | 0.846 | 0.801 | 0.775 | 0.834 | 0.806 | 0.782 | 0.830 | 0.809 | 0.783 | 0.835 |
| 7 | 0.825 | 0.798 | 0.857 | 0.821 | 0.791 | 0.847 | 0.801 | 0.775 | 0.830 | 0.802 | 0.773 | 0.837 | 0.809 | 0.786 | 0.838 |
| 8 | 0.827 | 0.797 | 0.847 | 0.823 | 0.799 | 0.848 | 0.798 | 0.772 | 0.832 | 0.805 | 0.779 | 0.834 | 0.814 | 0.792 | 0.839 |
| 9 | 0.829 | 0.801 | 0.857 | 0.821 | 0.797 | 0.843 | 0.799 | 0.773 | 0.822 | 0.801 | 0.770 | 0.828 | 0.816 | 0.789 | 0.844 |
| 10 | 0.826 | 0.806 | 0.854 | 0.823 | 0.797 | 0.848 | 0.802 | 0.777 | 0.827 | 0.802 | 0.780 | 0.829 | 0.814 | 0.795 | 0.842 |
| 11 | 0.827 | 0.799 | 0.856 | 0.823 | 0.796 | 0.853 | 0.801 | 0.776 | 0.832 | 0.805 | 0.780 | 0.830 | 0.813 | 0.792 | 0.835 |
| 12 | 0.828 | 0.800 | 0.852 | 0.823 | 0.797 | 0.849 | 0.799 | 0.772 | 0.830 | 0.804 | 0.777 | 0.833 | 0.809 | 0.783 | 0.835 |
| 13 | 0.826 | 0.799 | 0.855 | 0.823 | 0.792 | 0.848 | 0.803 | 0.771 | 0.834 | 0.804 | 0.780 | 0.827 | 0.816 | 0.792 | 0.840 |
| 14 | 0.828 | 0.798 | 0.852 | 0.824 | 0.801 | 0.850 | 0.802 | 0.776 | 0.833 | 0.807 | 0.772 | 0.831 | 0.808 | 0.779 | 0.831 |
| 15 | 0.830 | 0.800 | 0.856 | 0.824 | 0.798 | 0.845 | 0.799 | 0.774 | 0.824 | 0.804 | 0.777 | 0.827 | 0.805 | 0.781 | 0.826 |
| 16 | 0.831 | 0.804 | 0.858 | 0.822 | 0.799 | 0.842 | 0.804 | 0.778 | 0.832 | 0.809 | 0.783 | 0.833 | 0.813 | 0.793 | 0.839 |
| 17 | 0.830 | 0.810 | 0.858 | 0.825 | 0.801 | 0.850 | 0.801 | 0.777 | 0.828 | 0.804 | 0.776 | 0.832 | 0.812 | 0.785 | 0.834 |
| 18 | 0.829 | 0.802 | 0.854 | 0.829 | 0.808 | 0.851 | 0.803 | 0.778 | 0.828 | 0.807 | 0.779 | 0.834 | 0.813 | 0.788 | 0.837 |
| 19 | 0.832 | 0.806 | 0.855 | 0.827 | 0.802 | 0.852 | 0.801 | 0.773 | 0.829 | 0.805 | 0.775 | 0.833 | 0.815 | 0.790 | 0.841 |
| 20 | 0.828 | 0.806 | 0.854 | 0.824 | 0.795 | 0.852 | 0.801 | 0.771 | 0.833 | 0.801 | 0.780 | 0.837 | 0.810 | 0.786 | 0.837 |
| 21 | 0.829 | 0.804 | 0.849 | 0.824 | 0.802 | 0.852 | 0.805 | 0.775 | 0.831 | 0.806 | 0.782 | 0.830 | 0.810 | 0.787 | 0.830 |
| 22 | 0.826 | 0.801 | 0.852 | 0.821 | 0.796 | 0.842 | 0.802 | 0.774 | 0.834 | 0.805 | 0.774 | 0.832 | 0.812 | 0.789 | 0.840 |
| 23 | 0.829 | 0.804 | 0.854 | 0.826 | 0.801 | 0.852 | 0.803 | 0.773 | 0.829 | 0.806 | 0.782 | 0.832 | 0.810 | 0.786 | 0.831 |
| 24 | 0.830 | 0.805 | 0.856 | 0.827 | 0.800 | 0.854 | 0.801 | 0.775 | 0.830 | 0.806 | 0.770 | 0.835 | 0.812 | 0.785 | 0.839 |
| 25 | 0.828 | 0.801 | 0.853 | 0.827 | 0.804 | 0.851 | 0.800 | 0.772 | 0.823 | 0.806 | 0.777 | 0.829 | 0.813 | 0.786 | 0.836 |
| 26 | 0.829 | 0.805 | 0.850 | 0.826 | 0.802 | 0.849 | 0.801 | 0.774 | 0.828 | 0.806 | 0.779 | 0.832 | 0.812 | 0.781 | 0.838 |
| 27 | 0.828 | 0.807 | 0.848 | 0.825 | 0.803 | 0.852 | 0.801 | 0.769 | 0.832 | 0.805 | 0.776 | 0.835 | 0.810 | 0.774 | 0.833 |
| 28 | 0.828 | 0.804 | 0.854 | 0.825 | 0.796 | 0.854 | 0.805 | 0.776 | 0.833 | 0.807 | 0.783 | 0.832 | 0.812 | 0.792 | 0.831 |
| 29 | 0.828 | 0.799 | 0.851 | 0.827 | 0.800 | 0.851 | 0.801 | 0.776 | 0.832 | 0.803 | 0.772 | 0.835 | 0.811 | 0.788 | 0.836 |
| 30 | 0.826 | 0.801 | 0.848 | 0.824 | 0.794 | 0.846 | 0.801 | 0.774 | 0.826 | 0.802 | 0.778 | 0.835 | 0.806 | 0.784 | 0.828 |
| 31 | 0.825 | 0.800 | 0.857 | 0.823 | 0.800 | 0.844 | 0.801 | 0.771 | 0.830 | 0.805 | 0.779 | 0.833 | 0.808 | 0.786 | 0.836 |
| 32 | 0.831 | 0.807 | 0.856 | 0.822 | 0.799 | 0.846 | 0.800 | 0.773 | 0.832 | 0.803 | 0.775 | 0.838 | 0.807 | 0.785 | 0.836 |
| 33 | 0.826 | 0.797 | 0.850 | 0.824 | 0.796 | 0.847 | 0.803 | 0.777 | 0.826 | 0.806 | 0.779 | 0.830 | 0.816 | 0.789 | 0.836 |
| 34 | 0.831 | 0.809 | 0.855 | 0.825 | 0.802 | 0.851 | 0.803 | 0.780 | 0.831 | 0.806 | 0.773 | 0.832 | 0.811 | 0.789 | 0.841 |
| 35 | 0.828 | 0.802 | 0.850 | 0.826 | 0.797 | 0.850 | 0.801 | 0.776 | 0.827 | 0.806 | 0.779 | 0.830 | 0.816 | 0.790 | 0.845 |
| 36 | 0.825 | 0.794 | 0.847 | 0.823 | 0.798 | 0.845 | 0.801 | 0.774 | 0.838 | 0.806 | 0.781 | 0.835 | 0.818 | 0.795 | 0.839 |
| 37 | 0.827 | 0.798 | 0.852 | 0.821 | 0.793 | 0.843 | 0.798 | 0.774 | 0.827 | 0.803 | 0.772 | 0.836 | 0.810 | 0.788 | 0.838 |
| 38 | 0.827 | 0.798 | 0.850 | 0.823 | 0.801 | 0.851 | 0.800 | 0.770 | 0.826 | 0.803 | 0.779 | 0.834 | 0.809 | 0.784 | 0.831 |
| 39 | 0.831 | 0.804 | 0.851 | 0.826 | 0.799 | 0.851 | 0.801 | 0.772 | 0.832 | 0.806 | 0.775 | 0.836 | 0.810 | 0.783 | 0.835 |
| 40 | 0.826 | 0.803 | 0.856 | 0.822 | 0.796 | 0.849 | 0.799 | 0.771 | 0.822 | 0.798 | 0.774 | 0.832 | 0.811 | 0.786 | 0.832 |
| **Total mean** and 95% CI | **0.828** | 0.802 | 0.853 | **0.824** | 0.798 | 0.849 | **0.801** | 0.774 | 0.829 | **0.804** | 0.777 | 0.833 | **0.811** | 0.787 | 0.836 |
| 10-year balanced accuracy | | | | | | | | | | | | | | | |
|  | CoxSf | | | CoxEn | | | Feedforward | | | Densenet | | | Tab transformer | | |
| imp | mean | 95% lci | 95% uci | mean | 95% lci | 95% uci | mean | 95% lci | 95% uci | mean | 95% lci | 95% uci | mean | 95% lci | 95% uci |
| 1 | 0.806 | 0.784 | 0.825 | 0.800 | 0.770 | 0.825 | 0.782 | 0.751 | 0.809 | 0.787 | 0.763 | 0.811 | 0.799 | 0.774 | 0.821 |
| 2 | 0.811 | 0.787 | 0.833 | 0.800 | 0.780 | 0.820 | 0.783 | 0.756 | 0.810 | 0.785 | 0.761 | 0.806 | 0.797 | 0.775 | 0.821 |
| 3 | 0.808 | 0.781 | 0.831 | 0.801 | 0.782 | 0.826 | 0.780 | 0.750 | 0.807 | 0.786 | 0.760 | 0.809 | 0.795 | 0.772 | 0.820 |
| 4 | 0.807 | 0.785 | 0.831 | 0.801 | 0.780 | 0.822 | 0.780 | 0.755 | 0.808 | 0.787 | 0.759 | 0.811 | 0.798 | 0.781 | 0.822 |
| 5 | 0.806 | 0.785 | 0.830 | 0.802 | 0.776 | 0.824 | 0.777 | 0.754 | 0.804 | 0.785 | 0.761 | 0.812 | 0.791 | 0.769 | 0.810 |
| 6 | 0.807 | 0.784 | 0.831 | 0.798 | 0.775 | 0.822 | 0.781 | 0.754 | 0.806 | 0.788 | 0.762 | 0.809 | 0.798 | 0.775 | 0.821 |
| 7 | 0.806 | 0.782 | 0.830 | 0.798 | 0.769 | 0.818 | 0.781 | 0.751 | 0.804 | 0.786 | 0.761 | 0.818 | 0.799 | 0.775 | 0.821 |
| 8 | 0.807 | 0.785 | 0.831 | 0.799 | 0.779 | 0.824 | 0.781 | 0.756 | 0.812 | 0.788 | 0.761 | 0.812 | 0.798 | 0.776 | 0.821 |
| 9 | 0.808 | 0.784 | 0.829 | 0.801 | 0.778 | 0.823 | 0.780 | 0.750 | 0.806 | 0.786 | 0.759 | 0.812 | 0.803 | 0.776 | 0.827 |
| 10 | 0.807 | 0.787 | 0.829 | 0.801 | 0.776 | 0.820 | 0.782 | 0.758 | 0.808 | 0.786 | 0.758 | 0.809 | 0.798 | 0.776 | 0.826 |
| 11 | 0.807 | 0.782 | 0.831 | 0.801 | 0.775 | 0.831 | 0.781 | 0.748 | 0.803 | 0.787 | 0.762 | 0.813 | 0.800 | 0.777 | 0.820 |
| 12 | 0.806 | 0.784 | 0.829 | 0.800 | 0.775 | 0.823 | 0.783 | 0.757 | 0.810 | 0.788 | 0.764 | 0.810 | 0.796 | 0.773 | 0.819 |
| 13 | 0.807 | 0.787 | 0.829 | 0.804 | 0.783 | 0.824 | 0.781 | 0.753 | 0.811 | 0.785 | 0.759 | 0.811 | 0.801 | 0.776 | 0.818 |
| 14 | 0.806 | 0.785 | 0.832 | 0.801 | 0.778 | 0.819 | 0.781 | 0.756 | 0.809 | 0.789 | 0.761 | 0.812 | 0.793 | 0.775 | 0.816 |
| 15 | 0.809 | 0.788 | 0.832 | 0.803 | 0.780 | 0.825 | 0.781 | 0.756 | 0.808 | 0.787 | 0.763 | 0.814 | 0.795 | 0.768 | 0.819 |
| 16 | 0.806 | 0.781 | 0.834 | 0.800 | 0.779 | 0.821 | 0.783 | 0.755 | 0.808 | 0.789 | 0.763 | 0.811 | 0.799 | 0.774 | 0.819 |
| 17 | 0.808 | 0.788 | 0.832 | 0.802 | 0.783 | 0.826 | 0.780 | 0.760 | 0.804 | 0.788 | 0.760 | 0.817 | 0.795 | 0.770 | 0.820 |
| 18 | 0.808 | 0.785 | 0.830 | 0.807 | 0.785 | 0.829 | 0.784 | 0.762 | 0.812 | 0.791 | 0.762 | 0.814 | 0.799 | 0.774 | 0.820 |
| 19 | 0.809 | 0.789 | 0.830 | 0.802 | 0.780 | 0.820 | 0.781 | 0.752 | 0.802 | 0.786 | 0.758 | 0.814 | 0.803 | 0.782 | 0.825 |
| 20 | 0.809 | 0.785 | 0.831 | 0.802 | 0.781 | 0.824 | 0.784 | 0.761 | 0.813 | 0.786 | 0.758 | 0.811 | 0.797 | 0.776 | 0.819 |
| 21 | 0.806 | 0.781 | 0.828 | 0.800 | 0.776 | 0.827 | 0.782 | 0.753 | 0.803 | 0.787 | 0.763 | 0.810 | 0.794 | 0.771 | 0.817 |
| 22 | 0.808 | 0.787 | 0.827 | 0.800 | 0.780 | 0.822 | 0.786 | 0.758 | 0.819 | 0.789 | 0.767 | 0.809 | 0.802 | 0.778 | 0.824 |
| 23 | 0.807 | 0.781 | 0.830 | 0.802 | 0.781 | 0.827 | 0.780 | 0.750 | 0.804 | 0.786 | 0.761 | 0.811 | 0.796 | 0.774 | 0.818 |
| 24 | 0.807 | 0.787 | 0.824 | 0.803 | 0.780 | 0.831 | 0.782 | 0.760 | 0.804 | 0.790 | 0.762 | 0.813 | 0.803 | 0.776 | 0.827 |
| 25 | 0.806 | 0.784 | 0.827 | 0.800 | 0.773 | 0.825 | 0.780 | 0.754 | 0.805 | 0.788 | 0.761 | 0.810 | 0.798 | 0.769 | 0.820 |
| 26 | 0.808 | 0.784 | 0.832 | 0.803 | 0.782 | 0.829 | 0.783 | 0.754 | 0.803 | 0.790 | 0.766 | 0.814 | 0.797 | 0.776 | 0.820 |
| 27 | 0.809 | 0.786 | 0.831 | 0.803 | 0.782 | 0.828 | 0.785 | 0.760 | 0.814 | 0.789 | 0.764 | 0.816 | 0.802 | 0.774 | 0.822 |
| 28 | 0.808 | 0.789 | 0.829 | 0.800 | 0.780 | 0.829 | 0.781 | 0.755 | 0.810 | 0.790 | 0.766 | 0.811 | 0.797 | 0.774 | 0.820 |
| 29 | 0.807 | 0.784 | 0.832 | 0.802 | 0.777 | 0.821 | 0.781 | 0.755 | 0.806 | 0.787 | 0.761 | 0.814 | 0.795 | 0.774 | 0.821 |
| 30 | 0.805 | 0.782 | 0.827 | 0.801 | 0.777 | 0.823 | 0.781 | 0.755 | 0.808 | 0.785 | 0.760 | 0.806 | 0.796 | 0.777 | 0.826 |
| 31 | 0.806 | 0.786 | 0.830 | 0.799 | 0.774 | 0.824 | 0.783 | 0.751 | 0.806 | 0.789 | 0.766 | 0.813 | 0.800 | 0.780 | 0.824 |
| 32 | 0.808 | 0.785 | 0.833 | 0.801 | 0.779 | 0.826 | 0.784 | 0.759 | 0.808 | 0.786 | 0.763 | 0.805 | 0.799 | 0.775 | 0.825 |
| 33 | 0.807 | 0.782 | 0.827 | 0.802 | 0.777 | 0.826 | 0.780 | 0.750 | 0.809 | 0.787 | 0.759 | 0.811 | 0.802 | 0.779 | 0.824 |
| 34 | 0.807 | 0.789 | 0.825 | 0.802 | 0.780 | 0.826 | 0.781 | 0.757 | 0.810 | 0.788 | 0.759 | 0.813 | 0.798 | 0.777 | 0.820 |
| 35 | 0.805 | 0.781 | 0.833 | 0.801 | 0.779 | 0.827 | 0.781 | 0.758 | 0.805 | 0.787 | 0.765 | 0.812 | 0.801 | 0.779 | 0.822 |
| 36 | 0.806 | 0.784 | 0.827 | 0.802 | 0.778 | 0.821 | 0.782 | 0.756 | 0.807 | 0.788 | 0.763 | 0.809 | 0.804 | 0.779 | 0.829 |
| 37 | 0.803 | 0.784 | 0.823 | 0.799 | 0.777 | 0.821 | 0.779 | 0.756 | 0.804 | 0.787 | 0.760 | 0.813 | 0.802 | 0.778 | 0.825 |
| 38 | 0.807 | 0.779 | 0.830 | 0.800 | 0.782 | 0.821 | 0.781 | 0.751 | 0.801 | 0.789 | 0.762 | 0.814 | 0.795 | 0.774 | 0.820 |
| 39 | 0.808 | 0.781 | 0.832 | 0.803 | 0.779 | 0.827 | 0.784 | 0.760 | 0.811 | 0.789 | 0.762 | 0.819 | 0.801 | 0.780 | 0.821 |
| 40 | 0.803 | 0.778 | 0.825 | 0.798 | 0.773 | 0.819 | 0.780 | 0.754 | 0.810 | 0.783 | 0.759 | 0.806 | 0.796 | 0.774 | 0.814 |
| **Total mean** and 95% CI | **0.807** | 0.784 | 0.830 | **0.801** | 0.778 | 0.824 | **0.782** | 0.755 | 0.808 | **0.787** | 0.762 | 0.812 | **0.798** | 0.775 | 0.821 |
| 12-year balanced accuracy | | | | | | | | | | | | | | | |
|  | CoxSf | | | CoxEn | | | Feedforward | | | Densenet | | | Tab transformer | | |
| imp | mean | 95% lci | 95% uci | mean | 95% lci | 95% uci | mean | 95% lci | 95% uci | mean | 95% lci | 95% uci | mean | 95% lci | 95% uci |
| 1 | 0.729 | 0.708 | 0.751 | 0.717 | 0.694 | 0.744 | 0.711 | 0.014 | 0.684 | 0.715 | 0.691 | 0.743 | 0.734 | 0.708 | 0.762 |
| 2 | 0.733 | 0.707 | 0.759 | 0.721 | 0.701 | 0.748 | 0.710 | 0.014 | 0.677 | 0.714 | 0.686 | 0.735 | 0.728 | 0.705 | 0.747 |
| 3 | 0.728 | 0.707 | 0.749 | 0.723 | 0.704 | 0.749 | 0.708 | 0.014 | 0.685 | 0.713 | 0.682 | 0.744 | 0.731 | 0.708 | 0.758 |
| 4 | 0.727 | 0.704 | 0.751 | 0.722 | 0.696 | 0.746 | 0.706 | 0.016 | 0.681 | 0.712 | 0.689 | 0.736 | 0.731 | 0.706 | 0.751 |
| 5 | 0.732 | 0.703 | 0.756 | 0.722 | 0.695 | 0.749 | 0.709 | 0.015 | 0.684 | 0.714 | 0.688 | 0.745 | 0.727 | 0.704 | 0.752 |
| 6 | 0.732 | 0.712 | 0.756 | 0.724 | 0.702 | 0.742 | 0.710 | 0.014 | 0.683 | 0.718 | 0.697 | 0.747 | 0.734 | 0.704 | 0.760 |
| 7 | 0.729 | 0.699 | 0.759 | 0.720 | 0.699 | 0.743 | 0.710 | 0.015 | 0.684 | 0.714 | 0.682 | 0.742 | 0.732 | 0.710 | 0.760 |
| 8 | 0.729 | 0.704 | 0.758 | 0.719 | 0.696 | 0.747 | 0.713 | 0.014 | 0.678 | 0.714 | 0.685 | 0.744 | 0.733 | 0.703 | 0.755 |
| 9 | 0.728 | 0.704 | 0.751 | 0.723 | 0.698 | 0.747 | 0.704 | 0.013 | 0.680 | 0.714 | 0.688 | 0.741 | 0.733 | 0.708 | 0.756 |
| 10 | 0.730 | 0.702 | 0.755 | 0.721 | 0.698 | 0.743 | 0.708 | 0.015 | 0.681 | 0.714 | 0.692 | 0.737 | 0.734 | 0.709 | 0.756 |
| 11 | 0.729 | 0.707 | 0.753 | 0.722 | 0.696 | 0.752 | 0.709 | 0.013 | 0.683 | 0.713 | 0.685 | 0.741 | 0.735 | 0.709 | 0.759 |
| 12 | 0.727 | 0.708 | 0.752 | 0.720 | 0.697 | 0.744 | 0.710 | 0.015 | 0.677 | 0.716 | 0.689 | 0.741 | 0.734 | 0.706 | 0.759 |
| 13 | 0.730 | 0.708 | 0.750 | 0.723 | 0.696 | 0.750 | 0.706 | 0.014 | 0.681 | 0.712 | 0.681 | 0.737 | 0.733 | 0.704 | 0.760 |
| 14 | 0.730 | 0.707 | 0.754 | 0.720 | 0.700 | 0.747 | 0.709 | 0.015 | 0.681 | 0.714 | 0.693 | 0.743 | 0.729 | 0.697 | 0.753 |
| 15 | 0.728 | 0.706 | 0.751 | 0.722 | 0.693 | 0.748 | 0.710 | 0.015 | 0.679 | 0.715 | 0.690 | 0.744 | 0.729 | 0.708 | 0.752 |
| 16 | 0.731 | 0.709 | 0.755 | 0.722 | 0.700 | 0.751 | 0.710 | 0.014 | 0.683 | 0.717 | 0.693 | 0.742 | 0.733 | 0.709 | 0.756 |
| 17 | 0.728 | 0.707 | 0.752 | 0.721 | 0.689 | 0.742 | 0.708 | 0.015 | 0.683 | 0.714 | 0.686 | 0.744 | 0.731 | 0.703 | 0.756 |
| 18 | 0.725 | 0.700 | 0.748 | 0.722 | 0.694 | 0.747 | 0.711 | 0.014 | 0.684 | 0.714 | 0.688 | 0.741 | 0.731 | 0.710 | 0.761 |
| 19 | 0.731 | 0.708 | 0.755 | 0.722 | 0.698 | 0.747 | 0.710 | 0.015 | 0.683 | 0.717 | 0.687 | 0.745 | 0.731 | 0.711 | 0.760 |
| 20 | 0.731 | 0.706 | 0.760 | 0.721 | 0.697 | 0.747 | 0.712 | 0.014 | 0.680 | 0.714 | 0.694 | 0.738 | 0.727 | 0.708 | 0.752 |
| 21 | 0.732 | 0.703 | 0.757 | 0.722 | 0.702 | 0.746 | 0.711 | 0.013 | 0.685 | 0.716 | 0.688 | 0.741 | 0.729 | 0.704 | 0.753 |
| 22 | 0.732 | 0.705 | 0.752 | 0.722 | 0.699 | 0.744 | 0.714 | 0.014 | 0.687 | 0.716 | 0.692 | 0.736 | 0.736 | 0.713 | 0.768 |
| 23 | 0.730 | 0.705 | 0.754 | 0.724 | 0.702 | 0.750 | 0.706 | 0.013 | 0.683 | 0.712 | 0.679 | 0.737 | 0.725 | 0.702 | 0.750 |
| 24 | 0.730 | 0.707 | 0.756 | 0.724 | 0.706 | 0.750 | 0.711 | 0.015 | 0.683 | 0.716 | 0.692 | 0.742 | 0.735 | 0.711 | 0.760 |
| 25 | 0.730 | 0.701 | 0.756 | 0.719 | 0.696 | 0.743 | 0.707 | 0.015 | 0.682 | 0.714 | 0.688 | 0.742 | 0.726 | 0.699 | 0.747 |
| 26 | 0.727 | 0.699 | 0.750 | 0.721 | 0.698 | 0.750 | 0.711 | 0.015 | 0.682 | 0.717 | 0.694 | 0.743 | 0.733 | 0.709 | 0.759 |
| 27 | 0.729 | 0.706 | 0.752 | 0.724 | 0.701 | 0.746 | 0.712 | 0.015 | 0.678 | 0.713 | 0.686 | 0.741 | 0.733 | 0.703 | 0.755 |
| 28 | 0.730 | 0.706 | 0.759 | 0.725 | 0.702 | 0.747 | 0.708 | 0.014 | 0.682 | 0.717 | 0.690 | 0.744 | 0.733 | 0.707 | 0.758 |
| 29 | 0.728 | 0.704 | 0.757 | 0.718 | 0.697 | 0.744 | 0.707 | 0.016 | 0.679 | 0.716 | 0.684 | 0.743 | 0.733 | 0.711 | 0.761 |
| 30 | 0.728 | 0.703 | 0.748 | 0.721 | 0.698 | 0.747 | 0.711 | 0.015 | 0.686 | 0.714 | 0.691 | 0.741 | 0.733 | 0.706 | 0.761 |
| 31 | 0.726 | 0.700 | 0.750 | 0.720 | 0.689 | 0.748 | 0.713 | 0.014 | 0.681 | 0.716 | 0.691 | 0.741 | 0.735 | 0.715 | 0.760 |
| 32 | 0.728 | 0.705 | 0.753 | 0.719 | 0.695 | 0.743 | 0.712 | 0.015 | 0.682 | 0.717 | 0.689 | 0.745 | 0.731 | 0.709 | 0.757 |
| 33 | 0.729 | 0.703 | 0.752 | 0.722 | 0.695 | 0.751 | 0.706 | 0.012 | 0.685 | 0.713 | 0.682 | 0.741 | 0.732 | 0.711 | 0.765 |
| 34 | 0.730 | 0.705 | 0.754 | 0.722 | 0.701 | 0.747 | 0.709 | 0.016 | 0.680 | 0.713 | 0.689 | 0.737 | 0.734 | 0.704 | 0.758 |
| 35 | 0.726 | 0.701 | 0.750 | 0.721 | 0.698 | 0.743 | 0.711 | 0.014 | 0.686 | 0.716 | 0.691 | 0.743 | 0.730 | 0.705 | 0.750 |
| 36 | 0.730 | 0.705 | 0.753 | 0.723 | 0.698 | 0.751 | 0.712 | 0.014 | 0.685 | 0.718 | 0.695 | 0.751 | 0.733 | 0.706 | 0.754 |
| 37 | 0.728 | 0.705 | 0.754 | 0.720 | 0.694 | 0.743 | 0.707 | 0.014 | 0.682 | 0.715 | 0.690 | 0.740 | 0.734 | 0.709 | 0.758 |
| 38 | 0.732 | 0.706 | 0.754 | 0.724 | 0.700 | 0.751 | 0.710 | 0.014 | 0.683 | 0.714 | 0.683 | 0.743 | 0.732 | 0.708 | 0.759 |
| 39 | 0.730 | 0.706 | 0.750 | 0.724 | 0.696 | 0.745 | 0.714 | 0.014 | 0.685 | 0.718 | 0.689 | 0.745 | 0.733 | 0.705 | 0.763 |
| 40 | 0.730 | 0.701 | 0.758 | 0.721 | 0.697 | 0.747 | 0.705 | 0.013 | 0.683 | 0.712 | 0.692 | 0.740 | 0.730 | 0.711 | 0.753 |
| **Total mean** and 95% CI | **0.729** | 0.705 | 0.754 | **0.722** | 0.698 | 0.747 | **0.710** | 0.014 | 0.682 | **0.715** | 0.689 | 0.742 | **0.732** | 0.707 | 0.757 |

Abbreviations: CoxSf: Cox models with selected features; CoxEn: Cox models with Elastic Net regularization; FeedForward: Feedforward neural network; Densenet: Densely Connected Convolutional Network; TabTransformer: TabTransformer neural network; imp: imputation number; LCI: 95% lower confidence interval; UCI: 95% upper confidence interval; 95% CI: 95% confidence intervals

## Supplementary Table 6. Time-dependent sensitivity mean and 95% confidence intervals

| 4-year sensitivity | | | | | | | | | | | | | | | |
| --- | --- | --- | --- | --- | --- | --- | --- | --- | --- | --- | --- | --- | --- | --- | --- |
|  | CoxSf | | | CoxEn | | | Feedforward | | | Densenet | | | Tab transformer | | |
| imp | mean | 95% lci | 95% uci | mean | 95% lci | 95% uci | mean | 95% lci | 95% uci | mean | 95% lci | 95% uci | mean | 95% lci | 95% uci |
| 1 | 0.839 | 0.691 | 0.952 | 0.834 | 0.715 | 0.960 | 0.838 | 0.702 | 0.961 | 0.838 | 0.702 | 0.960 | 0.834 | 0.713 | 0.960 |
| 2 | 0.836 | 0.715 | 1.000 | 0.834 | 0.687 | 0.959 | 0.823 | 0.673 | 0.961 | 0.842 | 0.705 | 1.000 | 0.804 | 0.654 | 0.899 |
| 3 | 0.837 | 0.717 | 0.956 | 0.838 | 0.699 | 0.982 | 0.834 | 0.677 | 0.962 | 0.831 | 0.664 | 0.955 | 0.794 | 0.660 | 0.949 |
| 4 | 0.844 | 0.743 | 0.940 | 0.835 | 0.706 | 0.981 | 0.833 | 0.680 | 0.956 | 0.841 | 0.706 | 0.982 | 0.822 | 0.725 | 0.925 |
| 5 | 0.824 | 0.714 | 0.941 | 0.829 | 0.705 | 0.964 | 0.834 | 0.691 | 0.963 | 0.829 | 0.695 | 0.952 | 0.830 | 0.678 | 0.952 |
| 6 | 0.831 | 0.707 | 0.928 | 0.822 | 0.666 | 0.962 | 0.845 | 0.702 | 0.961 | 0.851 | 0.698 | 0.962 | 0.804 | 0.691 | 0.929 |
| 7 | 0.834 | 0.708 | 0.929 | 0.839 | 0.706 | 0.961 | 0.829 | 0.690 | 0.961 | 0.836 | 0.718 | 0.960 | 0.803 | 0.692 | 0.951 |
| 8 | 0.833 | 0.736 | 0.964 | 0.812 | 0.704 | 0.923 | 0.835 | 0.691 | 0.958 | 0.836 | 0.709 | 0.957 | 0.826 | 0.692 | 0.925 |
| 9 | 0.824 | 0.698 | 0.924 | 0.832 | 0.726 | 0.949 | 0.819 | 0.665 | 0.960 | 0.812 | 0.645 | 0.953 | 0.848 | 0.676 | 0.955 |
| 10 | 0.830 | 0.709 | 0.956 | 0.826 | 0.677 | 0.957 | 0.836 | 0.677 | 0.982 | 0.837 | 0.660 | 0.984 | 0.833 | 0.708 | 0.946 |
| 11 | 0.837 | 0.735 | 0.949 | 0.828 | 0.706 | 0.936 | 0.820 | 0.676 | 0.962 | 0.846 | 0.728 | 0.960 | 0.838 | 0.696 | 0.952 |
| 12 | 0.811 | 0.710 | 0.937 | 0.823 | 0.687 | 0.937 | 0.838 | 0.667 | 0.962 | 0.836 | 0.696 | 0.961 | 0.807 | 0.648 | 0.904 |
| 13 | 0.827 | 0.719 | 0.941 | 0.833 | 0.724 | 0.959 | 0.823 | 0.677 | 0.961 | 0.826 | 0.647 | 0.950 | 0.832 | 0.697 | 0.927 |
| 14 | 0.827 | 0.703 | 0.938 | 0.844 | 0.712 | 0.944 | 0.835 | 0.672 | 0.956 | 0.836 | 0.687 | 0.961 | 0.820 | 0.689 | 0.919 |
| 15 | 0.831 | 0.713 | 0.957 | 0.847 | 0.717 | 0.964 | 0.834 | 0.685 | 0.964 | 0.858 | 0.689 | 0.959 | 0.787 | 0.638 | 0.912 |
| 16 | 0.828 | 0.712 | 0.956 | 0.820 | 0.703 | 0.921 | 0.826 | 0.691 | 0.958 | 0.839 | 0.728 | 0.959 | 0.808 | 0.684 | 0.921 |
| 17 | 0.835 | 0.731 | 0.985 | 0.823 | 0.716 | 0.952 | 0.834 | 0.675 | 0.965 | 0.835 | 0.667 | 0.963 | 0.831 | 0.650 | 0.955 |
| 18 | 0.831 | 0.721 | 0.951 | 0.832 | 0.695 | 0.959 | 0.830 | 0.676 | 0.948 | 0.850 | 0.714 | 0.960 | 0.788 | 0.632 | 0.952 |
| 19 | 0.839 | 0.718 | 0.960 | 0.837 | 0.732 | 0.953 | 0.831 | 0.684 | 0.960 | 0.812 | 0.621 | 0.954 | 0.860 | 0.725 | 0.956 |
| 20 | 0.851 | 0.720 | 0.957 | 0.837 | 0.684 | 0.968 | 0.843 | 0.718 | 0.984 | 0.832 | 0.676 | 0.962 | 0.831 | 0.696 | 0.940 |
| 21 | 0.827 | 0.696 | 0.942 | 0.836 | 0.691 | 0.961 | 0.842 | 0.715 | 0.982 | 0.845 | 0.684 | 0.981 | 0.830 | 0.735 | 0.940 |
| 22 | 0.822 | 0.706 | 0.920 | 0.841 | 0.722 | 0.946 | 0.837 | 0.687 | 0.982 | 0.842 | 0.681 | 1.000 | 0.808 | 0.707 | 0.958 |
| 23 | 0.837 | 0.724 | 0.956 | 0.831 | 0.719 | 0.957 | 0.838 | 0.694 | 0.959 | 0.828 | 0.685 | 0.960 | 0.833 | 0.721 | 0.948 |
| 24 | 0.842 | 0.717 | 0.955 | 0.844 | 0.734 | 0.983 | 0.841 | 0.685 | 0.964 | 0.838 | 0.713 | 0.962 | 0.783 | 0.670 | 0.910 |
| 25 | 0.835 | 0.724 | 0.953 | 0.834 | 0.723 | 0.965 | 0.838 | 0.678 | 0.958 | 0.848 | 0.720 | 0.959 | 0.834 | 0.699 | 0.917 |
| 26 | 0.823 | 0.723 | 0.938 | 0.833 | 0.669 | 0.959 | 0.831 | 0.688 | 0.961 | 0.849 | 0.703 | 0.962 | 0.826 | 0.696 | 0.946 |
| 27 | 0.815 | 0.706 | 0.921 | 0.823 | 0.704 | 0.936 | 0.839 | 0.704 | 0.984 | 0.838 | 0.700 | 0.964 | 0.783 | 0.672 | 0.913 |
| 28 | 0.824 | 0.701 | 0.953 | 0.815 | 0.690 | 0.957 | 0.824 | 0.651 | 0.956 | 0.838 | 0.679 | 0.958 | 0.801 | 0.701 | 0.921 |
| 29 | 0.828 | 0.719 | 0.949 | 0.841 | 0.711 | 0.957 | 0.831 | 0.687 | 0.963 | 0.827 | 0.631 | 0.960 | 0.790 | 0.656 | 0.936 |
| 30 | 0.840 | 0.712 | 0.956 | 0.830 | 0.698 | 0.964 | 0.829 | 0.691 | 0.946 | 0.822 | 0.647 | 0.964 | 0.827 | 0.712 | 0.939 |
| 31 | 0.834 | 0.710 | 0.980 | 0.841 | 0.712 | 0.959 | 0.843 | 0.718 | 1.000 | 0.852 | 0.715 | 0.983 | 0.817 | 0.681 | 0.954 |
| 32 | 0.824 | 0.718 | 0.944 | 0.837 | 0.691 | 0.953 | 0.837 | 0.710 | 0.984 | 0.836 | 0.714 | 0.964 | 0.795 | 0.692 | 0.919 |
| 33 | 0.829 | 0.712 | 0.936 | 0.836 | 0.678 | 0.959 | 0.843 | 0.702 | 1.000 | 0.847 | 0.648 | 0.986 | 0.818 | 0.689 | 0.923 |
| 34 | 0.832 | 0.714 | 0.960 | 0.842 | 0.721 | 0.962 | 0.831 | 0.665 | 0.955 | 0.833 | 0.687 | 0.962 | 0.810 | 0.629 | 0.933 |
| 35 | 0.840 | 0.719 | 0.925 | 0.838 | 0.719 | 0.963 | 0.839 | 0.707 | 0.961 | 0.844 | 0.707 | 0.963 | 0.811 | 0.664 | 0.950 |
| 36 | 0.838 | 0.740 | 0.937 | 0.835 | 0.707 | 0.952 | 0.835 | 0.709 | 0.961 | 0.826 | 0.690 | 0.959 | 0.861 | 0.739 | 0.955 |
| 37 | 0.835 | 0.704 | 0.950 | 0.832 | 0.713 | 0.963 | 0.835 | 0.695 | 0.958 | 0.832 | 0.687 | 0.958 | 0.830 | 0.736 | 0.916 |
| 38 | 0.842 | 0.717 | 0.954 | 0.830 | 0.696 | 0.956 | 0.831 | 0.677 | 0.962 | 0.835 | 0.665 | 0.958 | 0.831 | 0.692 | 0.955 |
| 39 | 0.841 | 0.714 | 0.954 | 0.842 | 0.689 | 0.958 | 0.837 | 0.682 | 1.000 | 0.829 | 0.654 | 0.958 | 0.816 | 0.686 | 0.924 |
| 40 | 0.844 | 0.725 | 0.959 | 0.832 | 0.706 | 0.962 | 0.842 | 0.718 | 0.962 | 0.844 | 0.681 | 0.964 | 0.808 | 0.678 | 0.940 |
| **Total mean** and 95% CI | **0.832** | 0.716 | 0.949 | **0.833** | 0.704 | 0.957 | **0.834** | 0.688 | 0.966 | **0.837** | 0.686 | 0.965 | **0.818** | 0.688 | 0.936 |
| 6-year sensitivity | | | | | | | | | | | | | | | |
|  | CoxSf | | | CoxEn | | | Feedforward | | | Densenet | | | Tab transformer | | |
| imp | mean | 95% lci | 95% uci | mean | 95% lci | 95% uci | mean | 95% lci | 95% uci | mean | 95% lci | 95% uci | mean | 95% lci | 95% uci |
| 1 | 0.815 | 0.720 | 0.922 | 0.812 | 0.718 | 0.906 | 0.800 | 0.700 | 0.886 | 0.802 | 0.717 | 0.888 | 0.793 | 0.703 | 0.877 |
| 2 | 0.839 | 0.725 | 0.949 | 0.828 | 0.707 | 0.943 | 0.799 | 0.689 | 0.915 | 0.802 | 0.665 | 0.924 | 0.777 | 0.671 | 0.880 |
| 3 | 0.818 | 0.726 | 0.914 | 0.809 | 0.712 | 0.919 | 0.795 | 0.669 | 0.898 | 0.789 | 0.673 | 0.899 | 0.797 | 0.699 | 0.881 |
| 4 | 0.816 | 0.731 | 0.912 | 0.821 | 0.713 | 0.937 | 0.805 | 0.681 | 0.891 | 0.807 | 0.716 | 0.918 | 0.788 | 0.718 | 0.860 |
| 5 | 0.821 | 0.711 | 0.932 | 0.815 | 0.692 | 0.909 | 0.790 | 0.691 | 0.892 | 0.803 | 0.692 | 0.927 | 0.800 | 0.722 | 0.890 |
| 6 | 0.825 | 0.697 | 0.921 | 0.816 | 0.695 | 0.933 | 0.806 | 0.716 | 0.916 | 0.808 | 0.685 | 0.939 | 0.771 | 0.686 | 0.860 |
| 7 | 0.824 | 0.716 | 0.914 | 0.813 | 0.708 | 0.905 | 0.801 | 0.677 | 0.921 | 0.794 | 0.706 | 0.899 | 0.791 | 0.727 | 0.893 |
| 8 | 0.816 | 0.725 | 0.923 | 0.818 | 0.704 | 0.910 | 0.792 | 0.674 | 0.922 | 0.806 | 0.687 | 0.922 | 0.787 | 0.711 | 0.863 |
| 9 | 0.824 | 0.703 | 0.926 | 0.818 | 0.721 | 0.947 | 0.785 | 0.683 | 0.910 | 0.782 | 0.668 | 0.896 | 0.792 | 0.692 | 0.872 |
| 10 | 0.818 | 0.708 | 0.941 | 0.810 | 0.716 | 0.919 | 0.790 | 0.681 | 0.892 | 0.811 | 0.712 | 0.919 | 0.814 | 0.736 | 0.874 |
| 11 | 0.815 | 0.693 | 0.916 | 0.814 | 0.705 | 0.927 | 0.790 | 0.665 | 0.914 | 0.804 | 0.694 | 0.910 | 0.799 | 0.712 | 0.873 |
| 12 | 0.811 | 0.710 | 0.912 | 0.814 | 0.720 | 0.914 | 0.800 | 0.679 | 0.910 | 0.797 | 0.654 | 0.930 | 0.780 | 0.690 | 0.850 |
| 13 | 0.826 | 0.749 | 0.910 | 0.816 | 0.700 | 0.933 | 0.786 | 0.688 | 0.910 | 0.787 | 0.689 | 0.905 | 0.795 | 0.717 | 0.877 |
| 14 | 0.808 | 0.725 | 0.891 | 0.807 | 0.701 | 0.900 | 0.788 | 0.687 | 0.893 | 0.793 | 0.707 | 0.895 | 0.779 | 0.683 | 0.865 |
| 15 | 0.824 | 0.735 | 0.911 | 0.828 | 0.722 | 0.924 | 0.787 | 0.649 | 0.896 | 0.801 | 0.705 | 0.880 | 0.777 | 0.659 | 0.868 |
| 16 | 0.814 | 0.700 | 0.932 | 0.812 | 0.720 | 0.922 | 0.792 | 0.703 | 0.882 | 0.809 | 0.697 | 0.939 | 0.799 | 0.715 | 0.869 |
| 17 | 0.818 | 0.700 | 0.935 | 0.816 | 0.713 | 0.912 | 0.798 | 0.682 | 0.925 | 0.796 | 0.672 | 0.917 | 0.795 | 0.719 | 0.871 |
| 18 | 0.825 | 0.727 | 0.925 | 0.817 | 0.707 | 0.917 | 0.791 | 0.670 | 0.902 | 0.813 | 0.695 | 0.909 | 0.799 | 0.692 | 0.890 |
| 19 | 0.840 | 0.707 | 0.921 | 0.827 | 0.741 | 0.941 | 0.806 | 0.680 | 0.928 | 0.793 | 0.678 | 0.905 | 0.806 | 0.710 | 0.905 |
| 20 | 0.818 | 0.717 | 0.900 | 0.816 | 0.721 | 0.921 | 0.810 | 0.696 | 0.928 | 0.790 | 0.665 | 0.925 | 0.784 | 0.712 | 0.858 |
| 21 | 0.827 | 0.714 | 0.934 | 0.835 | 0.722 | 0.940 | 0.809 | 0.666 | 0.934 | 0.817 | 0.681 | 0.929 | 0.784 | 0.709 | 0.849 |
| 22 | 0.805 | 0.699 | 0.936 | 0.800 | 0.706 | 0.896 | 0.790 | 0.672 | 0.917 | 0.792 | 0.682 | 0.926 | 0.768 | 0.656 | 0.858 |
| 23 | 0.809 | 0.692 | 0.921 | 0.823 | 0.701 | 0.928 | 0.797 | 0.701 | 0.890 | 0.797 | 0.674 | 0.903 | 0.778 | 0.686 | 0.859 |
| 24 | 0.827 | 0.723 | 0.921 | 0.829 | 0.729 | 0.937 | 0.806 | 0.695 | 0.913 | 0.811 | 0.725 | 0.927 | 0.781 | 0.683 | 0.876 |
| 25 | 0.806 | 0.710 | 0.921 | 0.817 | 0.722 | 0.914 | 0.785 | 0.629 | 0.897 | 0.801 | 0.704 | 0.901 | 0.786 | 0.708 | 0.861 |
| 26 | 0.821 | 0.693 | 0.944 | 0.821 | 0.726 | 0.922 | 0.810 | 0.688 | 0.930 | 0.811 | 0.692 | 0.918 | 0.780 | 0.667 | 0.854 |
| 27 | 0.803 | 0.706 | 0.905 | 0.809 | 0.693 | 0.921 | 0.799 | 0.676 | 0.907 | 0.802 | 0.705 | 0.925 | 0.779 | 0.688 | 0.903 |
| 28 | 0.813 | 0.691 | 0.927 | 0.827 | 0.720 | 0.924 | 0.810 | 0.692 | 0.925 | 0.810 | 0.705 | 0.939 | 0.776 | 0.692 | 0.852 |
| 29 | 0.832 | 0.718 | 0.927 | 0.821 | 0.713 | 0.941 | 0.793 | 0.681 | 0.906 | 0.803 | 0.704 | 0.937 | 0.779 | 0.699 | 0.882 |
| 30 | 0.823 | 0.712 | 0.920 | 0.809 | 0.701 | 0.938 | 0.784 | 0.670 | 0.895 | 0.806 | 0.689 | 0.946 | 0.787 | 0.707 | 0.853 |
| 31 | 0.824 | 0.707 | 0.919 | 0.829 | 0.717 | 0.934 | 0.808 | 0.667 | 0.924 | 0.812 | 0.700 | 0.912 | 0.765 | 0.696 | 0.864 |
| 32 | 0.815 | 0.714 | 0.911 | 0.823 | 0.722 | 0.933 | 0.800 | 0.681 | 0.904 | 0.804 | 0.681 | 0.925 | 0.780 | 0.699 | 0.859 |
| 33 | 0.813 | 0.716 | 0.907 | 0.814 | 0.696 | 0.934 | 0.801 | 0.704 | 0.911 | 0.804 | 0.686 | 0.943 | 0.807 | 0.706 | 0.883 |
| 34 | 0.820 | 0.719 | 0.942 | 0.816 | 0.728 | 0.925 | 0.799 | 0.698 | 0.892 | 0.810 | 0.725 | 0.899 | 0.782 | 0.710 | 0.851 |
| 35 | 0.820 | 0.729 | 0.917 | 0.823 | 0.711 | 0.903 | 0.799 | 0.676 | 0.919 | 0.811 | 0.689 | 0.929 | 0.774 | 0.700 | 0.894 |
| 36 | 0.803 | 0.704 | 0.904 | 0.806 | 0.714 | 0.886 | 0.799 | 0.679 | 0.875 | 0.812 | 0.711 | 0.944 | 0.806 | 0.731 | 0.875 |
| 37 | 0.824 | 0.725 | 0.914 | 0.824 | 0.731 | 0.946 | 0.797 | 0.688 | 0.902 | 0.798 | 0.682 | 0.902 | 0.798 | 0.712 | 0.863 |
| 38 | 0.828 | 0.722 | 0.917 | 0.816 | 0.722 | 0.936 | 0.792 | 0.670 | 0.929 | 0.809 | 0.713 | 0.905 | 0.816 | 0.717 | 0.883 |
| 39 | 0.836 | 0.745 | 0.930 | 0.829 | 0.732 | 0.942 | 0.806 | 0.694 | 0.913 | 0.803 | 0.694 | 0.910 | 0.769 | 0.681 | 0.841 |
| 40 | 0.814 | 0.727 | 0.919 | 0.827 | 0.735 | 0.924 | 0.794 | 0.675 | 0.902 | 0.804 | 0.666 | 0.930 | 0.782 | 0.675 | 0.869 |
| **Total mean** and 95% CI | **0.819** | 0.715 | 0.921 | **0.818** | 0.714 | 0.924 | **0.797** | 0.682 | 0.908 | **0.803** | 0.692 | 0.917 | **0.787** | 0.700 | 0.870 |
| 8-year sensitivity | | | | | | | | | | | | | | | |
|  | CoxSf | | | CoxEn | | | Feedforward | | | Densenet | | | Tab transformer | | |
| imp | mean | 95% lci | 95% uci | mean | 95% lci | 95% uci | mean | 95% lci | 95% uci | mean | 95% lci | 95% uci | mean | 95% lci | 95% uci |
| 1 | 0.799 | 0.735 | 0.865 | 0.813 | 0.741 | 0.897 | 0.805 | 0.718 | 0.898 | 0.814 | 0.703 | 0.912 | 0.784 | 0.730 | 0.882 |
| 2 | 0.816 | 0.757 | 0.879 | 0.823 | 0.748 | 0.904 | 0.804 | 0.686 | 0.914 | 0.811 | 0.729 | 0.890 | 0.842 | 0.728 | 0.896 |
| 3 | 0.810 | 0.743 | 0.871 | 0.822 | 0.751 | 0.885 | 0.808 | 0.734 | 0.891 | 0.809 | 0.699 | 0.897 | 0.798 | 0.721 | 0.879 |
| 4 | 0.818 | 0.756 | 0.887 | 0.820 | 0.755 | 0.896 | 0.799 | 0.718 | 0.877 | 0.810 | 0.703 | 0.887 | 0.842 | 0.724 | 0.894 |
| 5 | 0.815 | 0.757 | 0.878 | 0.815 | 0.733 | 0.871 | 0.789 | 0.693 | 0.878 | 0.802 | 0.707 | 0.902 | 0.777 | 0.696 | 0.881 |
| 6 | 0.823 | 0.753 | 0.918 | 0.812 | 0.737 | 0.879 | 0.809 | 0.707 | 0.893 | 0.819 | 0.719 | 0.906 | 0.827 | 0.725 | 0.886 |
| 7 | 0.807 | 0.739 | 0.871 | 0.804 | 0.734 | 0.881 | 0.804 | 0.720 | 0.899 | 0.800 | 0.704 | 0.902 | 0.799 | 0.701 | 0.866 |
| 8 | 0.813 | 0.723 | 0.904 | 0.825 | 0.750 | 0.907 | 0.802 | 0.712 | 0.893 | 0.816 | 0.713 | 0.905 | 0.816 | 0.727 | 0.900 |
| 9 | 0.822 | 0.760 | 0.884 | 0.812 | 0.733 | 0.890 | 0.793 | 0.694 | 0.882 | 0.792 | 0.671 | 0.883 | 0.797 | 0.731 | 0.894 |
| 10 | 0.819 | 0.756 | 0.880 | 0.821 | 0.733 | 0.893 | 0.804 | 0.716 | 0.898 | 0.813 | 0.731 | 0.894 | 0.807 | 0.711 | 0.915 |
| 11 | 0.810 | 0.729 | 0.861 | 0.822 | 0.750 | 0.898 | 0.817 | 0.729 | 0.901 | 0.823 | 0.733 | 0.917 | 0.794 | 0.717 | 0.915 |
| 12 | 0.816 | 0.732 | 0.893 | 0.813 | 0.751 | 0.907 | 0.805 | 0.709 | 0.901 | 0.819 | 0.710 | 0.897 | 0.790 | 0.698 | 0.884 |
| 13 | 0.808 | 0.750 | 0.878 | 0.824 | 0.750 | 0.908 | 0.796 | 0.688 | 0.877 | 0.818 | 0.734 | 0.895 | 0.788 | 0.695 | 0.887 |
| 14 | 0.804 | 0.750 | 0.854 | 0.815 | 0.747 | 0.876 | 0.797 | 0.722 | 0.877 | 0.811 | 0.720 | 0.901 | 0.780 | 0.697 | 0.873 |
| 15 | 0.821 | 0.753 | 0.874 | 0.816 | 0.738 | 0.894 | 0.795 | 0.689 | 0.888 | 0.807 | 0.732 | 0.900 | 0.789 | 0.701 | 0.904 |
| 16 | 0.816 | 0.759 | 0.881 | 0.814 | 0.732 | 0.879 | 0.804 | 0.727 | 0.889 | 0.816 | 0.740 | 0.903 | 0.770 | 0.704 | 0.896 |
| 17 | 0.815 | 0.756 | 0.880 | 0.822 | 0.752 | 0.892 | 0.809 | 0.723 | 0.906 | 0.822 | 0.732 | 0.911 | 0.810 | 0.701 | 0.902 |
| 18 | 0.822 | 0.757 | 0.882 | 0.824 | 0.753 | 0.893 | 0.812 | 0.729 | 0.902 | 0.815 | 0.708 | 0.903 | 0.824 | 0.738 | 0.913 |
| 19 | 0.820 | 0.761 | 0.870 | 0.824 | 0.761 | 0.888 | 0.803 | 0.713 | 0.888 | 0.806 | 0.713 | 0.903 | 0.857 | 0.722 | 0.918 |
| 20 | 0.808 | 0.746 | 0.870 | 0.816 | 0.741 | 0.889 | 0.808 | 0.734 | 0.885 | 0.816 | 0.714 | 0.905 | 0.788 | 0.704 | 0.877 |
| 21 | 0.816 | 0.757 | 0.872 | 0.820 | 0.730 | 0.909 | 0.822 | 0.735 | 0.915 | 0.827 | 0.738 | 0.906 | 0.797 | 0.712 | 0.889 |
| 22 | 0.813 | 0.737 | 0.889 | 0.812 | 0.744 | 0.909 | 0.806 | 0.732 | 0.886 | 0.801 | 0.714 | 0.894 | 0.783 | 0.709 | 0.890 |
| 23 | 0.812 | 0.748 | 0.876 | 0.823 | 0.752 | 0.894 | 0.794 | 0.711 | 0.886 | 0.821 | 0.736 | 0.923 | 0.840 | 0.716 | 0.893 |
| 24 | 0.813 | 0.761 | 0.871 | 0.820 | 0.751 | 0.887 | 0.799 | 0.714 | 0.890 | 0.816 | 0.709 | 0.908 | 0.829 | 0.739 | 0.878 |
| 25 | 0.815 | 0.748 | 0.880 | 0.820 | 0.747 | 0.887 | 0.788 | 0.704 | 0.873 | 0.803 | 0.715 | 0.898 | 0.805 | 0.699 | 0.901 |
| 26 | 0.817 | 0.751 | 0.872 | 0.825 | 0.754 | 0.897 | 0.814 | 0.736 | 0.903 | 0.817 | 0.723 | 0.901 | 0.771 | 0.705 | 0.867 |
| 27 | 0.811 | 0.745 | 0.881 | 0.817 | 0.753 | 0.901 | 0.804 | 0.724 | 0.903 | 0.815 | 0.715 | 0.912 | 0.839 | 0.713 | 0.916 |
| 28 | 0.818 | 0.752 | 0.870 | 0.827 | 0.741 | 0.900 | 0.810 | 0.702 | 0.896 | 0.825 | 0.729 | 0.913 | 0.823 | 0.721 | 0.883 |
| 29 | 0.810 | 0.760 | 0.863 | 0.823 | 0.758 | 0.912 | 0.808 | 0.721 | 0.907 | 0.814 | 0.730 | 0.900 | 0.792 | 0.719 | 0.911 |
| 30 | 0.817 | 0.760 | 0.881 | 0.829 | 0.752 | 0.897 | 0.804 | 0.699 | 0.885 | 0.815 | 0.719 | 0.892 | 0.843 | 0.713 | 0.913 |
| 31 | 0.806 | 0.736 | 0.867 | 0.823 | 0.750 | 0.901 | 0.807 | 0.715 | 0.893 | 0.809 | 0.711 | 0.894 | 0.778 | 0.707 | 0.888 |
| 32 | 0.810 | 0.739 | 0.877 | 0.816 | 0.749 | 0.904 | 0.800 | 0.703 | 0.900 | 0.812 | 0.712 | 0.904 | 0.788 | 0.722 | 0.900 |
| 33 | 0.808 | 0.734 | 0.880 | 0.811 | 0.742 | 0.872 | 0.802 | 0.702 | 0.890 | 0.816 | 0.723 | 0.897 | 0.793 | 0.728 | 0.917 |
| 34 | 0.818 | 0.768 | 0.880 | 0.824 | 0.757 | 0.899 | 0.811 | 0.728 | 0.908 | 0.813 | 0.715 | 0.904 | 0.813 | 0.727 | 0.892 |
| 35 | 0.812 | 0.748 | 0.883 | 0.816 | 0.750 | 0.898 | 0.804 | 0.722 | 0.892 | 0.810 | 0.724 | 0.883 | 0.824 | 0.728 | 0.901 |
| 36 | 0.813 | 0.733 | 0.872 | 0.814 | 0.746 | 0.901 | 0.806 | 0.697 | 0.918 | 0.819 | 0.728 | 0.904 | 0.843 | 0.764 | 0.895 |
| 37 | 0.815 | 0.753 | 0.877 | 0.821 | 0.713 | 0.924 | 0.808 | 0.712 | 0.905 | 0.816 | 0.727 | 0.914 | 0.813 | 0.713 | 0.880 |
| 38 | 0.809 | 0.741 | 0.869 | 0.819 | 0.755 | 0.904 | 0.809 | 0.705 | 0.902 | 0.815 | 0.723 | 0.901 | 0.813 | 0.697 | 0.875 |
| 39 | 0.821 | 0.751 | 0.897 | 0.825 | 0.750 | 0.894 | 0.803 | 0.725 | 0.896 | 0.810 | 0.704 | 0.900 | 0.819 | 0.705 | 0.888 |
| 40 | 0.803 | 0.743 | 0.884 | 0.817 | 0.754 | 0.904 | 0.806 | 0.713 | 0.877 | 0.802 | 0.714 | 0.884 | 0.821 | 0.703 | 0.900 |
| **Total mean** and 95% CI | **0.813** | 0.748 | 0.878 | **0.819** | 0.746 | 0.895 | **0.804** | 0.714 | 0.894 | **0.813** | 0.718 | 0.901 | **0.808** | 0.715 | 0.893 |
| 10-year sensitivity | | | | | | | | | | | | | | | |
|  | CoxSf | | | CoxEn | | | Feedforward | | | Densenet | | | Tab transformer | | |
| imp | mean | 95% lci | 95% uci | mean | 95% lci | 95% uci | mean | 95% lci | 95% uci | mean | 95% lci | 95% uci | mean | 95% lci | 95% uci |
| 1 | 0.763 | 0.693 | 0.857 | 0.756 | 0.674 | 0.828 | 0.754 | 0.664 | 0.831 | 0.763 | 0.672 | 0.845 | 0.776 | 0.696 | 0.862 |
| 2 | 0.766 | 0.707 | 0.852 | 0.767 | 0.690 | 0.860 | 0.767 | 0.675 | 0.849 | 0.754 | 0.663 | 0.829 | 0.785 | 0.707 | 0.852 |
| 3 | 0.768 | 0.687 | 0.851 | 0.766 | 0.694 | 0.838 | 0.752 | 0.670 | 0.840 | 0.762 | 0.673 | 0.844 | 0.769 | 0.696 | 0.830 |
| 4 | 0.757 | 0.683 | 0.856 | 0.761 | 0.670 | 0.841 | 0.746 | 0.650 | 0.834 | 0.756 | 0.666 | 0.842 | 0.772 | 0.713 | 0.818 |
| 5 | 0.778 | 0.690 | 0.871 | 0.769 | 0.705 | 0.834 | 0.746 | 0.646 | 0.849 | 0.763 | 0.651 | 0.828 | 0.773 | 0.690 | 0.849 |
| 6 | 0.777 | 0.707 | 0.873 | 0.761 | 0.687 | 0.838 | 0.756 | 0.657 | 0.854 | 0.760 | 0.672 | 0.840 | 0.778 | 0.690 | 0.832 |
| 7 | 0.760 | 0.675 | 0.854 | 0.742 | 0.677 | 0.815 | 0.745 | 0.660 | 0.840 | 0.759 | 0.677 | 0.855 | 0.757 | 0.691 | 0.824 |
| 8 | 0.776 | 0.701 | 0.849 | 0.762 | 0.684 | 0.850 | 0.762 | 0.663 | 0.846 | 0.774 | 0.696 | 0.873 | 0.782 | 0.722 | 0.835 |
| 9 | 0.762 | 0.686 | 0.850 | 0.764 | 0.675 | 0.862 | 0.755 | 0.647 | 0.848 | 0.760 | 0.677 | 0.838 | 0.768 | 0.675 | 0.820 |
| 10 | 0.767 | 0.686 | 0.847 | 0.759 | 0.671 | 0.853 | 0.750 | 0.675 | 0.836 | 0.766 | 0.684 | 0.844 | 0.779 | 0.701 | 0.848 |
| 11 | 0.758 | 0.681 | 0.845 | 0.766 | 0.681 | 0.847 | 0.757 | 0.657 | 0.842 | 0.765 | 0.677 | 0.841 | 0.779 | 0.709 | 0.876 |
| 12 | 0.776 | 0.702 | 0.860 | 0.761 | 0.685 | 0.838 | 0.762 | 0.669 | 0.844 | 0.769 | 0.692 | 0.849 | 0.774 | 0.688 | 0.846 |
| 13 | 0.767 | 0.695 | 0.854 | 0.768 | 0.701 | 0.856 | 0.750 | 0.666 | 0.827 | 0.761 | 0.665 | 0.858 | 0.765 | 0.656 | 0.828 |
| 14 | 0.761 | 0.688 | 0.827 | 0.756 | 0.684 | 0.845 | 0.754 | 0.662 | 0.847 | 0.773 | 0.694 | 0.854 | 0.759 | 0.684 | 0.824 |
| 15 | 0.766 | 0.711 | 0.849 | 0.763 | 0.680 | 0.839 | 0.755 | 0.666 | 0.843 | 0.760 | 0.676 | 0.834 | 0.768 | 0.679 | 0.865 |
| 16 | 0.772 | 0.681 | 0.869 | 0.766 | 0.677 | 0.862 | 0.764 | 0.661 | 0.862 | 0.767 | 0.675 | 0.860 | 0.800 | 0.737 | 0.867 |
| 17 | 0.763 | 0.696 | 0.828 | 0.768 | 0.692 | 0.850 | 0.755 | 0.653 | 0.844 | 0.765 | 0.692 | 0.849 | 0.801 | 0.657 | 0.850 |
| 18 | 0.774 | 0.702 | 0.868 | 0.776 | 0.697 | 0.858 | 0.765 | 0.678 | 0.849 | 0.765 | 0.682 | 0.844 | 0.764 | 0.686 | 0.855 |
| 19 | 0.759 | 0.694 | 0.824 | 0.758 | 0.695 | 0.829 | 0.748 | 0.660 | 0.860 | 0.756 | 0.657 | 0.835 | 0.803 | 0.710 | 0.850 |
| 20 | 0.761 | 0.696 | 0.857 | 0.760 | 0.702 | 0.840 | 0.766 | 0.685 | 0.857 | 0.770 | 0.672 | 0.854 | 0.770 | 0.669 | 0.830 |
| 21 | 0.756 | 0.683 | 0.854 | 0.768 | 0.698 | 0.856 | 0.752 | 0.675 | 0.838 | 0.764 | 0.670 | 0.850 | 0.779 | 0.689 | 0.834 |
| 22 | 0.781 | 0.698 | 0.873 | 0.767 | 0.685 | 0.851 | 0.762 | 0.689 | 0.853 | 0.755 | 0.679 | 0.836 | 0.786 | 0.689 | 0.844 |
| 23 | 0.761 | 0.687 | 0.854 | 0.764 | 0.674 | 0.854 | 0.751 | 0.672 | 0.826 | 0.759 | 0.677 | 0.851 | 0.789 | 0.719 | 0.833 |
| 24 | 0.764 | 0.698 | 0.864 | 0.765 | 0.692 | 0.859 | 0.759 | 0.667 | 0.843 | 0.769 | 0.684 | 0.844 | 0.784 | 0.696 | 0.856 |
| 25 | 0.772 | 0.704 | 0.859 | 0.764 | 0.674 | 0.851 | 0.747 | 0.652 | 0.836 | 0.773 | 0.686 | 0.864 | 0.778 | 0.703 | 0.859 |
| 26 | 0.775 | 0.702 | 0.851 | 0.771 | 0.696 | 0.837 | 0.759 | 0.675 | 0.839 | 0.768 | 0.687 | 0.851 | 0.790 | 0.663 | 0.841 |
| 27 | 0.769 | 0.693 | 0.869 | 0.747 | 0.683 | 0.831 | 0.761 | 0.679 | 0.840 | 0.758 | 0.685 | 0.843 | 0.782 | 0.710 | 0.845 |
| 28 | 0.768 | 0.697 | 0.858 | 0.765 | 0.690 | 0.840 | 0.762 | 0.661 | 0.838 | 0.769 | 0.681 | 0.857 | 0.774 | 0.712 | 0.858 |
| 29 | 0.765 | 0.683 | 0.871 | 0.757 | 0.683 | 0.834 | 0.749 | 0.665 | 0.827 | 0.769 | 0.687 | 0.848 | 0.756 | 0.683 | 0.836 |
| 30 | 0.773 | 0.689 | 0.847 | 0.766 | 0.690 | 0.847 | 0.758 | 0.664 | 0.847 | 0.769 | 0.678 | 0.853 | 0.804 | 0.696 | 0.863 |
| 31 | 0.763 | 0.680 | 0.856 | 0.762 | 0.678 | 0.840 | 0.755 | 0.651 | 0.847 | 0.764 | 0.671 | 0.856 | 0.791 | 0.689 | 0.860 |
| 32 | 0.759 | 0.684 | 0.852 | 0.764 | 0.690 | 0.854 | 0.760 | 0.669 | 0.857 | 0.763 | 0.686 | 0.852 | 0.754 | 0.698 | 0.848 |
| 33 | 0.763 | 0.693 | 0.855 | 0.765 | 0.697 | 0.837 | 0.754 | 0.650 | 0.832 | 0.770 | 0.692 | 0.844 | 0.768 | 0.699 | 0.862 |
| 34 | 0.770 | 0.704 | 0.866 | 0.761 | 0.683 | 0.835 | 0.754 | 0.662 | 0.836 | 0.766 | 0.685 | 0.849 | 0.773 | 0.713 | 0.880 |
| 35 | 0.747 | 0.682 | 0.833 | 0.759 | 0.682 | 0.845 | 0.752 | 0.654 | 0.849 | 0.755 | 0.679 | 0.844 | 0.770 | 0.719 | 0.851 |
| 36 | 0.771 | 0.688 | 0.856 | 0.755 | 0.686 | 0.843 | 0.756 | 0.655 | 0.843 | 0.771 | 0.679 | 0.846 | 0.771 | 0.707 | 0.840 |
| 37 | 0.769 | 0.688 | 0.860 | 0.764 | 0.677 | 0.853 | 0.755 | 0.665 | 0.849 | 0.765 | 0.662 | 0.858 | 0.780 | 0.714 | 0.837 |
| 38 | 0.768 | 0.679 | 0.868 | 0.756 | 0.691 | 0.829 | 0.753 | 0.645 | 0.827 | 0.762 | 0.692 | 0.835 | 0.766 | 0.717 | 0.818 |
| 39 | 0.770 | 0.685 | 0.859 | 0.761 | 0.704 | 0.836 | 0.762 | 0.663 | 0.840 | 0.767 | 0.664 | 0.848 | 0.788 | 0.712 | 0.845 |
| 40 | 0.769 | 0.686 | 0.850 | 0.761 | 0.684 | 0.837 | 0.756 | 0.644 | 0.844 | 0.760 | 0.662 | 0.859 | 0.784 | 0.690 | 0.841 |
| **Total mean** and 95% CI | **0.767** | 0.692 | 0.855 | **0.762** | 0.686 | 0.844 | **0.756** | 0.663 | 0.843 | **0.764** | 0.677 | 0.848 | **0.777** | 0.697 | 0.845 |
| 12-year sensitivity | | | | | | | | | | | | | | | |
|  | CoxSf | | | CoxEn | | | Feedforward | | | Densenet | | | Tab transformer | | |
| imp | mean | 95% lci | 95% uci | mean | 95% lci | 95% uci | mean | 95% lci | 95% uci | mean | 95% lci | 95% uci | mean | 95% lci | 95% uci |
| 1 | 0.659 | 0.574 | 0.732 | 0.625 | 0.506 | 0.749 | 0.641 | 0.541 | 0.764 | 0.645 | 0.541 | 0.761 | 0.688 | 0.605 | 0.769 |
| 2 | 0.674 | 0.572 | 0.744 | 0.649 | 0.540 | 0.766 | 0.650 | 0.510 | 0.781 | 0.649 | 0.521 | 0.772 | 0.674 | 0.552 | 0.788 |
| 3 | 0.657 | 0.556 | 0.726 | 0.627 | 0.516 | 0.758 | 0.631 | 0.509 | 0.757 | 0.644 | 0.513 | 0.764 | 0.647 | 0.574 | 0.711 |
| 4 | 0.650 | 0.543 | 0.726 | 0.632 | 0.514 | 0.761 | 0.627 | 0.516 | 0.779 | 0.643 | 0.519 | 0.736 | 0.655 | 0.539 | 0.763 |
| 5 | 0.665 | 0.574 | 0.744 | 0.630 | 0.524 | 0.737 | 0.651 | 0.514 | 0.796 | 0.647 | 0.527 | 0.769 | 0.648 | 0.529 | 0.765 |
| 6 | 0.677 | 0.579 | 0.735 | 0.651 | 0.537 | 0.794 | 0.646 | 0.526 | 0.791 | 0.660 | 0.547 | 0.763 | 0.640 | 0.584 | 0.717 |
| 7 | 0.656 | 0.549 | 0.727 | 0.628 | 0.526 | 0.755 | 0.639 | 0.526 | 0.759 | 0.647 | 0.530 | 0.752 | 0.632 | 0.558 | 0.762 |
| 8 | 0.661 | 0.548 | 0.735 | 0.642 | 0.538 | 0.762 | 0.645 | 0.523 | 0.761 | 0.656 | 0.530 | 0.760 | 0.654 | 0.560 | 0.761 |
| 9 | 0.653 | 0.523 | 0.732 | 0.632 | 0.550 | 0.764 | 0.632 | 0.520 | 0.772 | 0.653 | 0.549 | 0.744 | 0.635 | 0.557 | 0.762 |
| 10 | 0.663 | 0.565 | 0.742 | 0.638 | 0.520 | 0.766 | 0.623 | 0.520 | 0.749 | 0.654 | 0.546 | 0.758 | 0.663 | 0.561 | 0.738 |
| 11 | 0.666 | 0.581 | 0.731 | 0.650 | 0.532 | 0.773 | 0.641 | 0.516 | 0.759 | 0.655 | 0.557 | 0.760 | 0.645 | 0.588 | 0.744 |
| 12 | 0.659 | 0.571 | 0.730 | 0.639 | 0.520 | 0.764 | 0.641 | 0.515 | 0.761 | 0.658 | 0.521 | 0.752 | 0.656 | 0.578 | 0.735 |
| 13 | 0.672 | 0.546 | 0.751 | 0.642 | 0.535 | 0.749 | 0.621 | 0.490 | 0.765 | 0.642 | 0.514 | 0.766 | 0.648 | 0.574 | 0.716 |
| 14 | 0.668 | 0.574 | 0.740 | 0.628 | 0.516 | 0.753 | 0.630 | 0.517 | 0.736 | 0.653 | 0.528 | 0.772 | 0.644 | 0.550 | 0.805 |
| 15 | 0.656 | 0.549 | 0.733 | 0.640 | 0.535 | 0.767 | 0.647 | 0.522 | 0.782 | 0.637 | 0.508 | 0.749 | 0.631 | 0.551 | 0.739 |
| 16 | 0.665 | 0.557 | 0.725 | 0.639 | 0.500 | 0.770 | 0.644 | 0.531 | 0.779 | 0.644 | 0.511 | 0.757 | 0.674 | 0.592 | 0.764 |
| 17 | 0.649 | 0.552 | 0.733 | 0.632 | 0.509 | 0.761 | 0.630 | 0.513 | 0.748 | 0.651 | 0.525 | 0.769 | 0.685 | 0.575 | 0.758 |
| 18 | 0.658 | 0.569 | 0.732 | 0.641 | 0.550 | 0.765 | 0.631 | 0.519 | 0.750 | 0.643 | 0.521 | 0.742 | 0.674 | 0.534 | 0.782 |
| 19 | 0.664 | 0.546 | 0.746 | 0.632 | 0.529 | 0.787 | 0.639 | 0.522 | 0.784 | 0.659 | 0.562 | 0.766 | 0.654 | 0.563 | 0.722 |
| 20 | 0.665 | 0.553 | 0.745 | 0.632 | 0.523 | 0.744 | 0.649 | 0.521 | 0.755 | 0.657 | 0.526 | 0.773 | 0.634 | 0.556 | 0.792 |
| 21 | 0.671 | 0.558 | 0.767 | 0.632 | 0.539 | 0.768 | 0.625 | 0.508 | 0.761 | 0.646 | 0.541 | 0.765 | 0.637 | 0.511 | 0.785 |
| 22 | 0.668 | 0.557 | 0.751 | 0.640 | 0.531 | 0.820 | 0.657 | 0.532 | 0.774 | 0.652 | 0.518 | 0.763 | 0.672 | 0.609 | 0.729 |
| 23 | 0.654 | 0.559 | 0.744 | 0.644 | 0.544 | 0.764 | 0.627 | 0.506 | 0.747 | 0.642 | 0.499 | 0.770 | 0.639 | 0.560 | 0.789 |
| 24 | 0.674 | 0.591 | 0.749 | 0.649 | 0.520 | 0.773 | 0.641 | 0.525 | 0.794 | 0.655 | 0.549 | 0.747 | 0.689 | 0.566 | 0.791 |
| 25 | 0.650 | 0.550 | 0.729 | 0.633 | 0.530 | 0.774 | 0.648 | 0.517 | 0.777 | 0.644 | 0.511 | 0.773 | 0.670 | 0.491 | 0.733 |
| 26 | 0.655 | 0.582 | 0.736 | 0.635 | 0.543 | 0.765 | 0.635 | 0.530 | 0.750 | 0.647 | 0.548 | 0.751 | 0.686 | 0.517 | 0.800 |
| 27 | 0.657 | 0.544 | 0.734 | 0.632 | 0.522 | 0.732 | 0.648 | 0.509 | 0.770 | 0.638 | 0.517 | 0.743 | 0.631 | 0.577 | 0.772 |
| 28 | 0.671 | 0.547 | 0.737 | 0.646 | 0.557 | 0.752 | 0.635 | 0.492 | 0.756 | 0.663 | 0.557 | 0.768 | 0.670 | 0.575 | 0.742 |
| 29 | 0.656 | 0.540 | 0.741 | 0.633 | 0.509 | 0.750 | 0.628 | 0.513 | 0.767 | 0.656 | 0.557 | 0.743 | 0.678 | 0.621 | 0.745 |
| 30 | 0.653 | 0.559 | 0.732 | 0.628 | 0.540 | 0.715 | 0.658 | 0.537 | 0.796 | 0.653 | 0.527 | 0.763 | 0.667 | 0.577 | 0.732 |
| 31 | 0.657 | 0.540 | 0.731 | 0.621 | 0.506 | 0.753 | 0.640 | 0.532 | 0.785 | 0.653 | 0.542 | 0.775 | 0.671 | 0.590 | 0.750 |
| 32 | 0.668 | 0.560 | 0.742 | 0.641 | 0.506 | 0.782 | 0.641 | 0.496 | 0.767 | 0.662 | 0.528 | 0.766 | 0.657 | 0.552 | 0.745 |
| 33 | 0.659 | 0.571 | 0.730 | 0.639 | 0.537 | 0.758 | 0.639 | 0.517 | 0.763 | 0.649 | 0.509 | 0.792 | 0.638 | 0.585 | 0.722 |
| 34 | 0.650 | 0.555 | 0.725 | 0.625 | 0.538 | 0.742 | 0.644 | 0.511 | 0.759 | 0.651 | 0.518 | 0.748 | 0.624 | 0.571 | 0.733 |
| 35 | 0.656 | 0.548 | 0.724 | 0.622 | 0.543 | 0.739 | 0.634 | 0.515 | 0.750 | 0.636 | 0.520 | 0.772 | 0.645 | 0.570 | 0.731 |
| 36 | 0.658 | 0.523 | 0.743 | 0.643 | 0.520 | 0.767 | 0.657 | 0.529 | 0.781 | 0.659 | 0.557 | 0.768 | 0.646 | 0.562 | 0.765 |
| 37 | 0.662 | 0.570 | 0.736 | 0.645 | 0.519 | 0.784 | 0.622 | 0.507 | 0.729 | 0.641 | 0.546 | 0.750 | 0.658 | 0.590 | 0.722 |
| 38 | 0.662 | 0.578 | 0.734 | 0.629 | 0.529 | 0.770 | 0.632 | 0.502 | 0.747 | 0.637 | 0.538 | 0.742 | 0.649 | 0.575 | 0.763 |
| 39 | 0.656 | 0.567 | 0.722 | 0.621 | 0.513 | 0.711 | 0.641 | 0.518 | 0.753 | 0.642 | 0.540 | 0.745 | 0.648 | 0.560 | 0.728 |
| 40 | 0.664 | 0.579 | 0.752 | 0.639 | 0.507 | 0.761 | 0.631 | 0.498 | 0.756 | 0.649 | 0.565 | 0.739 | 0.627 | 0.584 | 0.704 |
| **Total mean** and 95% CI | **0.661** | 0.559 | 0.737 | **0.636** | 0.527 | 0.761 | **0.639** | 0.517 | 0.765 | **0.649** | 0.532 | 0.759 | **0.655** | 0.565 | 0.752 |

Abbreviations: CoxSf: Cox models with selected features; CoxEn: Cox models with Elastic Net regularization; FeedForward: Feedforward neural network; Densenet: Densely Connected Convolutional Network; TabTransformer: TabTransformer neural network; imp: imputation number; LCI: 95% lower confidence interval; UCI: 95% upper confidence interval; 95% CI: 95% confidence intervals

## Supplementary Table 7. Time-dependent specificity mean and 95% confidence intervals

| 4-year specificity | | | | | | | | | | | | | | | |
| --- | --- | --- | --- | --- | --- | --- | --- | --- | --- | --- | --- | --- | --- | --- | --- |
|  | CoxSf | | | CoxEn | | | Feedforward | | | Densenet | | | Tab transformer | | |
| imp | mean | 95% lci | 95% uci | mean | 95% lci | 95% uci | mean | 95% lci | 95% uci | mean | 95% lci | 95% uci | mean | 95% lci | 95% uci |
| 1 | 0.829 | 0.745 | 0.892 | 0.823 | 0.688 | 0.894 | 0.808 | 0.687 | 0.902 | 0.795 | 0.687 | 0.908 | 0.831 | 0.740 | 0.910 |
| 2 | 0.815 | 0.686 | 0.895 | 0.819 | 0.676 | 0.904 | 0.810 | 0.646 | 0.918 | 0.777 | 0.606 | 0.916 | 0.860 | 0.770 | 0.923 |
| 3 | 0.828 | 0.705 | 0.887 | 0.821 | 0.712 | 0.907 | 0.792 | 0.662 | 0.910 | 0.790 | 0.634 | 0.919 | 0.889 | 0.763 | 0.928 |
| 4 | 0.838 | 0.781 | 0.896 | 0.827 | 0.694 | 0.905 | 0.810 | 0.683 | 0.912 | 0.799 | 0.699 | 0.908 | 0.891 | 0.812 | 0.908 |
| 5 | 0.845 | 0.681 | 0.898 | 0.825 | 0.664 | 0.904 | 0.809 | 0.658 | 0.906 | 0.799 | 0.644 | 0.915 | 0.835 | 0.752 | 0.910 |
| 6 | 0.832 | 0.683 | 0.899 | 0.825 | 0.718 | 0.917 | 0.788 | 0.640 | 0.890 | 0.787 | 0.647 | 0.896 | 0.874 | 0.824 | 0.934 |
| 7 | 0.842 | 0.746 | 0.899 | 0.822 | 0.656 | 0.903 | 0.816 | 0.705 | 0.907 | 0.816 | 0.697 | 0.905 | 0.880 | 0.771 | 0.923 |
| 8 | 0.849 | 0.729 | 0.891 | 0.835 | 0.702 | 0.898 | 0.796 | 0.613 | 0.903 | 0.803 | 0.687 | 0.919 | 0.867 | 0.747 | 0.926 |
| 9 | 0.840 | 0.775 | 0.906 | 0.822 | 0.695 | 0.895 | 0.812 | 0.655 | 0.911 | 0.807 | 0.671 | 0.925 | 0.809 | 0.769 | 0.922 |
| 10 | 0.833 | 0.703 | 0.900 | 0.814 | 0.669 | 0.899 | 0.802 | 0.633 | 0.910 | 0.795 | 0.668 | 0.906 | 0.840 | 0.824 | 0.932 |
| 11 | 0.840 | 0.749 | 0.895 | 0.830 | 0.691 | 0.905 | 0.819 | 0.683 | 0.919 | 0.786 | 0.663 | 0.904 | 0.838 | 0.779 | 0.924 |
| 12 | 0.842 | 0.720 | 0.902 | 0.836 | 0.709 | 0.908 | 0.788 | 0.638 | 0.899 | 0.777 | 0.617 | 0.910 | 0.860 | 0.767 | 0.925 |
| 13 | 0.840 | 0.785 | 0.896 | 0.824 | 0.675 | 0.915 | 0.816 | 0.675 | 0.925 | 0.808 | 0.675 | 0.918 | 0.827 | 0.807 | 0.920 |
| 14 | 0.836 | 0.769 | 0.893 | 0.822 | 0.725 | 0.896 | 0.804 | 0.653 | 0.913 | 0.795 | 0.686 | 0.901 | 0.838 | 0.774 | 0.921 |
| 15 | 0.835 | 0.778 | 0.894 | 0.817 | 0.688 | 0.892 | 0.814 | 0.655 | 0.914 | 0.781 | 0.674 | 0.896 | 0.881 | 0.774 | 0.915 |
| 16 | 0.838 | 0.787 | 0.891 | 0.834 | 0.736 | 0.898 | 0.806 | 0.674 | 0.909 | 0.798 | 0.665 | 0.906 | 0.890 | 0.782 | 0.934 |
| 17 | 0.822 | 0.716 | 0.885 | 0.812 | 0.674 | 0.889 | 0.797 | 0.672 | 0.899 | 0.792 | 0.667 | 0.911 | 0.826 | 0.748 | 0.919 |
| 18 | 0.832 | 0.715 | 0.897 | 0.816 | 0.679 | 0.895 | 0.811 | 0.672 | 0.908 | 0.785 | 0.631 | 0.904 | 0.891 | 0.748 | 0.929 |
| 19 | 0.828 | 0.700 | 0.897 | 0.833 | 0.699 | 0.910 | 0.811 | 0.692 | 0.898 | 0.813 | 0.697 | 0.931 | 0.828 | 0.757 | 0.902 |
| 20 | 0.825 | 0.767 | 0.897 | 0.825 | 0.715 | 0.898 | 0.793 | 0.628 | 0.899 | 0.799 | 0.657 | 0.904 | 0.832 | 0.757 | 0.919 |
| 21 | 0.832 | 0.746 | 0.905 | 0.815 | 0.670 | 0.912 | 0.796 | 0.666 | 0.900 | 0.781 | 0.649 | 0.908 | 0.843 | 0.783 | 0.904 |
| 22 | 0.844 | 0.784 | 0.891 | 0.824 | 0.671 | 0.897 | 0.802 | 0.660 | 0.898 | 0.798 | 0.626 | 0.912 | 0.880 | 0.768 | 0.916 |
| 23 | 0.842 | 0.736 | 0.898 | 0.835 | 0.717 | 0.912 | 0.801 | 0.627 | 0.895 | 0.810 | 0.624 | 0.929 | 0.841 | 0.795 | 0.922 |
| 24 | 0.832 | 0.773 | 0.891 | 0.818 | 0.705 | 0.884 | 0.803 | 0.670 | 0.900 | 0.796 | 0.696 | 0.898 | 0.887 | 0.732 | 0.931 |
| 25 | 0.849 | 0.786 | 0.906 | 0.835 | 0.732 | 0.892 | 0.818 | 0.706 | 0.916 | 0.801 | 0.679 | 0.907 | 0.875 | 0.783 | 0.905 |
| 26 | 0.837 | 0.701 | 0.905 | 0.824 | 0.690 | 0.901 | 0.804 | 0.672 | 0.894 | 0.790 | 0.661 | 0.898 | 0.833 | 0.786 | 0.916 |
| 27 | 0.844 | 0.703 | 0.889 | 0.823 | 0.677 | 0.900 | 0.789 | 0.608 | 0.892 | 0.793 | 0.676 | 0.896 | 0.841 | 0.732 | 0.924 |
| 28 | 0.842 | 0.710 | 0.900 | 0.837 | 0.692 | 0.920 | 0.826 | 0.700 | 0.902 | 0.821 | 0.719 | 0.926 | 0.895 | 0.789 | 0.933 |
| 29 | 0.835 | 0.741 | 0.888 | 0.814 | 0.700 | 0.907 | 0.809 | 0.646 | 0.908 | 0.792 | 0.658 | 0.916 | 0.879 | 0.726 | 0.928 |
| 30 | 0.823 | 0.689 | 0.894 | 0.821 | 0.698 | 0.892 | 0.809 | 0.687 | 0.897 | 0.806 | 0.660 | 0.931 | 0.840 | 0.794 | 0.928 |
| 31 | 0.820 | 0.693 | 0.900 | 0.817 | 0.673 | 0.901 | 0.803 | 0.645 | 0.896 | 0.789 | 0.652 | 0.896 | 0.866 | 0.762 | 0.939 |
| 32 | 0.841 | 0.775 | 0.901 | 0.829 | 0.666 | 0.909 | 0.799 | 0.647 | 0.902 | 0.786 | 0.623 | 0.922 | 0.902 | 0.805 | 0.924 |
| 33 | 0.835 | 0.757 | 0.887 | 0.822 | 0.691 | 0.898 | 0.794 | 0.627 | 0.900 | 0.780 | 0.621 | 0.926 | 0.866 | 0.826 | 0.922 |
| 34 | 0.835 | 0.738 | 0.890 | 0.815 | 0.671 | 0.895 | 0.806 | 0.683 | 0.893 | 0.789 | 0.673 | 0.895 | 0.879 | 0.770 | 0.924 |
| 35 | 0.828 | 0.756 | 0.894 | 0.815 | 0.703 | 0.898 | 0.811 | 0.634 | 0.900 | 0.786 | 0.642 | 0.912 | 0.839 | 0.686 | 0.916 |
| 36 | 0.848 | 0.765 | 0.892 | 0.829 | 0.723 | 0.897 | 0.807 | 0.699 | 0.892 | 0.808 | 0.691 | 0.916 | 0.827 | 0.791 | 0.901 |
| 37 | 0.820 | 0.694 | 0.897 | 0.815 | 0.705 | 0.902 | 0.804 | 0.664 | 0.891 | 0.794 | 0.656 | 0.921 | 0.829 | 0.784 | 0.900 |
| 38 | 0.821 | 0.689 | 0.894 | 0.819 | 0.711 | 0.901 | 0.794 | 0.635 | 0.892 | 0.785 | 0.646 | 0.924 | 0.793 | 0.765 | 0.941 |
| 39 | 0.819 | 0.720 | 0.894 | 0.813 | 0.676 | 0.893 | 0.789 | 0.651 | 0.894 | 0.785 | 0.650 | 0.913 | 0.838 | 0.822 | 0.927 |
| 40 | 0.824 | 0.718 | 0.899 | 0.824 | 0.689 | 0.905 | 0.792 | 0.652 | 0.895 | 0.785 | 0.616 | 0.908 | 0.871 | 0.767 | 0.917 |
| **Total mean** and 95% CI | **0.834** | 0.735 | 0.896 | **0.823** | 0.693 | 0.901 | **0.804** | 0.660 | 0.903 | **0.794** | 0.660 | 0.911 | **0.855** | 0.773 | 0.921 |
| 6-year specificity | | | | | | | | | | | | | | | |
|  | CoxSf | | | CoxEn | | | Feedforward | | | Densenet | | | Tab transformer | | |
| imp | mean | 95% lci | 95% uci | mean | 95% lci | 95% uci | mean | 95% lci | 95% uci | mean | 95% lci | 95% uci | mean | 95% lci | 95% uci |
| 1 | 0.824 | 0.737 | 0.892 | 0.826 | 0.707 | 0.885 | 0.808 | 0.710 | 0.890 | 0.802 | 0.722 | 0.882 | 0.847 | 0.759 | 0.909 |
| 2 | 0.800 | 0.716 | 0.883 | 0.804 | 0.704 | 0.895 | 0.797 | 0.635 | 0.890 | 0.795 | 0.692 | 0.888 | 0.834 | 0.711 | 0.899 |
| 3 | 0.821 | 0.726 | 0.889 | 0.828 | 0.717 | 0.896 | 0.795 | 0.684 | 0.888 | 0.808 | 0.681 | 0.890 | 0.817 | 0.742 | 0.879 |
| 4 | 0.832 | 0.741 | 0.896 | 0.821 | 0.726 | 0.894 | 0.795 | 0.699 | 0.885 | 0.803 | 0.672 | 0.880 | 0.848 | 0.826 | 0.904 |
| 5 | 0.816 | 0.715 | 0.905 | 0.809 | 0.698 | 0.902 | 0.807 | 0.713 | 0.886 | 0.793 | 0.668 | 0.886 | 0.835 | 0.756 | 0.884 |
| 6 | 0.810 | 0.707 | 0.896 | 0.811 | 0.697 | 0.892 | 0.793 | 0.663 | 0.887 | 0.797 | 0.655 | 0.897 | 0.840 | 0.817 | 0.912 |
| 7 | 0.812 | 0.716 | 0.896 | 0.817 | 0.717 | 0.890 | 0.800 | 0.681 | 0.887 | 0.809 | 0.684 | 0.891 | 0.846 | 0.804 | 0.889 |
| 8 | 0.822 | 0.716 | 0.894 | 0.813 | 0.709 | 0.893 | 0.805 | 0.659 | 0.891 | 0.798 | 0.679 | 0.883 | 0.831 | 0.771 | 0.900 |
| 9 | 0.810 | 0.699 | 0.892 | 0.814 | 0.695 | 0.884 | 0.809 | 0.701 | 0.892 | 0.813 | 0.699 | 0.895 | 0.841 | 0.793 | 0.908 |
| 10 | 0.820 | 0.721 | 0.896 | 0.818 | 0.689 | 0.891 | 0.813 | 0.736 | 0.892 | 0.791 | 0.671 | 0.872 | 0.826 | 0.802 | 0.867 |
| 11 | 0.823 | 0.744 | 0.896 | 0.816 | 0.696 | 0.894 | 0.808 | 0.701 | 0.899 | 0.794 | 0.695 | 0.889 | 0.832 | 0.811 | 0.903 |
| 12 | 0.817 | 0.702 | 0.906 | 0.820 | 0.704 | 0.881 | 0.794 | 0.648 | 0.885 | 0.799 | 0.651 | 0.888 | 0.835 | 0.799 | 0.918 |
| 13 | 0.812 | 0.711 | 0.876 | 0.819 | 0.709 | 0.896 | 0.808 | 0.674 | 0.891 | 0.810 | 0.681 | 0.888 | 0.849 | 0.793 | 0.868 |
| 14 | 0.831 | 0.756 | 0.898 | 0.830 | 0.736 | 0.902 | 0.811 | 0.701 | 0.903 | 0.813 | 0.678 | 0.896 | 0.838 | 0.773 | 0.905 |
| 15 | 0.815 | 0.711 | 0.883 | 0.811 | 0.707 | 0.892 | 0.821 | 0.714 | 0.891 | 0.809 | 0.714 | 0.882 | 0.844 | 0.722 | 0.914 |
| 16 | 0.819 | 0.714 | 0.895 | 0.815 | 0.664 | 0.891 | 0.803 | 0.694 | 0.886 | 0.795 | 0.673 | 0.882 | 0.838 | 0.814 | 0.870 |
| 17 | 0.818 | 0.700 | 0.902 | 0.809 | 0.700 | 0.881 | 0.793 | 0.675 | 0.872 | 0.797 | 0.662 | 0.888 | 0.848 | 0.775 | 0.871 |
| 18 | 0.822 | 0.726 | 0.887 | 0.816 | 0.721 | 0.883 | 0.807 | 0.683 | 0.896 | 0.790 | 0.679 | 0.889 | 0.832 | 0.760 | 0.916 |
| 19 | 0.803 | 0.721 | 0.896 | 0.815 | 0.692 | 0.894 | 0.794 | 0.668 | 0.873 | 0.801 | 0.691 | 0.887 | 0.837 | 0.755 | 0.866 |
| 20 | 0.822 | 0.718 | 0.894 | 0.818 | 0.709 | 0.893 | 0.787 | 0.651 | 0.895 | 0.803 | 0.641 | 0.887 | 0.841 | 0.802 | 0.881 |
| 21 | 0.800 | 0.704 | 0.895 | 0.795 | 0.692 | 0.895 | 0.793 | 0.657 | 0.873 | 0.782 | 0.657 | 0.870 | 0.847 | 0.782 | 0.891 |
| 22 | 0.828 | 0.703 | 0.910 | 0.832 | 0.743 | 0.895 | 0.805 | 0.662 | 0.897 | 0.810 | 0.682 | 0.886 | 0.852 | 0.796 | 0.911 |
| 23 | 0.835 | 0.740 | 0.910 | 0.814 | 0.706 | 0.884 | 0.803 | 0.712 | 0.882 | 0.805 | 0.684 | 0.894 | 0.857 | 0.797 | 0.910 |
| 24 | 0.822 | 0.730 | 0.897 | 0.819 | 0.719 | 0.890 | 0.800 | 0.674 | 0.892 | 0.801 | 0.695 | 0.881 | 0.835 | 0.757 | 0.919 |
| 25 | 0.829 | 0.701 | 0.897 | 0.820 | 0.708 | 0.890 | 0.817 | 0.702 | 0.897 | 0.802 | 0.677 | 0.888 | 0.848 | 0.792 | 0.903 |
| 26 | 0.820 | 0.707 | 0.907 | 0.810 | 0.693 | 0.884 | 0.790 | 0.667 | 0.883 | 0.803 | 0.677 | 0.884 | 0.852 | 0.835 | 0.871 |
| 27 | 0.830 | 0.702 | 0.900 | 0.817 | 0.708 | 0.893 | 0.796 | 0.661 | 0.895 | 0.796 | 0.655 | 0.888 | 0.849 | 0.762 | 0.888 |
| 28 | 0.820 | 0.700 | 0.900 | 0.808 | 0.711 | 0.896 | 0.793 | 0.662 | 0.892 | 0.801 | 0.675 | 0.874 | 0.854 | 0.814 | 0.916 |
| 29 | 0.804 | 0.707 | 0.893 | 0.816 | 0.711 | 0.890 | 0.801 | 0.673 | 0.890 | 0.787 | 0.662 | 0.888 | 0.840 | 0.761 | 0.882 |
| 30 | 0.815 | 0.721 | 0.898 | 0.816 | 0.707 | 0.899 | 0.814 | 0.701 | 0.899 | 0.785 | 0.613 | 0.877 | 0.838 | 0.822 | 0.880 |
| 31 | 0.809 | 0.729 | 0.899 | 0.802 | 0.694 | 0.887 | 0.791 | 0.643 | 0.881 | 0.797 | 0.710 | 0.904 | 0.858 | 0.802 | 0.880 |
| 32 | 0.821 | 0.714 | 0.900 | 0.813 | 0.704 | 0.897 | 0.799 | 0.671 | 0.887 | 0.795 | 0.661 | 0.884 | 0.852 | 0.785 | 0.918 |
| 33 | 0.821 | 0.710 | 0.893 | 0.821 | 0.719 | 0.897 | 0.798 | 0.689 | 0.868 | 0.800 | 0.637 | 0.887 | 0.822 | 0.747 | 0.906 |
| 34 | 0.819 | 0.714 | 0.893 | 0.814 | 0.718 | 0.894 | 0.804 | 0.682 | 0.889 | 0.798 | 0.692 | 0.876 | 0.849 | 0.771 | 0.879 |
| 35 | 0.818 | 0.724 | 0.889 | 0.810 | 0.724 | 0.885 | 0.808 | 0.690 | 0.896 | 0.795 | 0.656 | 0.893 | 0.841 | 0.704 | 0.918 |
| 36 | 0.834 | 0.705 | 0.905 | 0.834 | 0.759 | 0.905 | 0.801 | 0.683 | 0.883 | 0.800 | 0.668 | 0.891 | 0.843 | 0.813 | 0.872 |
| 37 | 0.802 | 0.727 | 0.878 | 0.807 | 0.697 | 0.883 | 0.798 | 0.687 | 0.883 | 0.799 | 0.693 | 0.871 | 0.836 | 0.810 | 0.888 |
| 38 | 0.813 | 0.721 | 0.884 | 0.814 | 0.700 | 0.886 | 0.801 | 0.686 | 0.894 | 0.787 | 0.662 | 0.874 | 0.817 | 0.791 | 0.872 |
| 39 | 0.804 | 0.715 | 0.893 | 0.808 | 0.710 | 0.887 | 0.790 | 0.657 | 0.888 | 0.792 | 0.671 | 0.879 | 0.850 | 0.785 | 0.897 |
| 40 | 0.817 | 0.711 | 0.890 | 0.803 | 0.699 | 0.886 | 0.797 | 0.673 | 0.889 | 0.786 | 0.634 | 0.880 | 0.845 | 0.794 | 0.903 |
| **Total mean** and 95% CI | **0.818** | 0.717 | 0.895 | **0.815** | 0.708 | 0.891 | **0.801** | 0.681 | 0.888 | **0.798** | 0.674 | 0.885 | **0.841** | 0.783 | 0.894 |
| 8-year specificity | | | | | | | | | | | | | | | |
|  | CoxSf | | | CoxEn | | | Feedforward | | | Densenet | | | Tab transformer | | |
| imp | mean | 95% lci | 95% uci | mean | 95% lci | 95% uci | mean | 95% lci | 95% uci | mean | 95% lci | 95% uci | mean | 95% lci | 95% uci |
| 1 | 0.853 | 0.797 | 0.899 | 0.833 | 0.755 | 0.887 | 0.797 | 0.685 | 0.872 | 0.795 | 0.685 | 0.882 | 0.833 | 0.687 | 0.886 |
| 2 | 0.841 | 0.789 | 0.879 | 0.820 | 0.736 | 0.870 | 0.794 | 0.695 | 0.876 | 0.794 | 0.713 | 0.870 | 0.785 | 0.745 | 0.884 |
| 3 | 0.844 | 0.766 | 0.886 | 0.827 | 0.766 | 0.883 | 0.793 | 0.695 | 0.869 | 0.800 | 0.716 | 0.892 | 0.816 | 0.749 | 0.889 |
| 4 | 0.841 | 0.790 | 0.876 | 0.830 | 0.739 | 0.887 | 0.802 | 0.718 | 0.868 | 0.799 | 0.723 | 0.874 | 0.790 | 0.740 | 0.888 |
| 5 | 0.841 | 0.785 | 0.879 | 0.831 | 0.759 | 0.886 | 0.801 | 0.703 | 0.883 | 0.799 | 0.685 | 0.873 | 0.841 | 0.731 | 0.897 |
| 6 | 0.832 | 0.707 | 0.888 | 0.832 | 0.740 | 0.880 | 0.792 | 0.699 | 0.876 | 0.793 | 0.724 | 0.872 | 0.785 | 0.749 | 0.879 |
| 7 | 0.844 | 0.781 | 0.887 | 0.838 | 0.761 | 0.893 | 0.798 | 0.685 | 0.881 | 0.803 | 0.704 | 0.880 | 0.811 | 0.770 | 0.904 |
| 8 | 0.840 | 0.735 | 0.890 | 0.822 | 0.743 | 0.887 | 0.794 | 0.699 | 0.891 | 0.794 | 0.689 | 0.896 | 0.819 | 0.741 | 0.892 |
| 9 | 0.836 | 0.770 | 0.878 | 0.830 | 0.744 | 0.887 | 0.805 | 0.714 | 0.875 | 0.809 | 0.728 | 0.889 | 0.842 | 0.752 | 0.876 |
| 10 | 0.833 | 0.756 | 0.892 | 0.825 | 0.753 | 0.889 | 0.801 | 0.706 | 0.876 | 0.791 | 0.704 | 0.885 | 0.841 | 0.731 | 0.894 |
| 11 | 0.845 | 0.793 | 0.887 | 0.825 | 0.734 | 0.892 | 0.785 | 0.703 | 0.861 | 0.786 | 0.708 | 0.866 | 0.845 | 0.705 | 0.886 |
| 12 | 0.839 | 0.748 | 0.889 | 0.832 | 0.730 | 0.884 | 0.792 | 0.676 | 0.874 | 0.789 | 0.697 | 0.875 | 0.839 | 0.719 | 0.900 |
| 13 | 0.844 | 0.768 | 0.889 | 0.823 | 0.740 | 0.887 | 0.809 | 0.733 | 0.888 | 0.789 | 0.695 | 0.855 | 0.866 | 0.749 | 0.903 |
| 14 | 0.852 | 0.795 | 0.888 | 0.832 | 0.762 | 0.878 | 0.807 | 0.734 | 0.872 | 0.803 | 0.711 | 0.881 | 0.834 | 0.749 | 0.895 |
| 15 | 0.840 | 0.764 | 0.887 | 0.831 | 0.732 | 0.897 | 0.803 | 0.712 | 0.878 | 0.801 | 0.702 | 0.881 | 0.820 | 0.680 | 0.890 |
| 16 | 0.846 | 0.793 | 0.886 | 0.831 | 0.754 | 0.889 | 0.803 | 0.725 | 0.876 | 0.801 | 0.719 | 0.873 | 0.867 | 0.700 | 0.901 |
| 17 | 0.846 | 0.799 | 0.882 | 0.828 | 0.760 | 0.889 | 0.793 | 0.703 | 0.876 | 0.787 | 0.684 | 0.862 | 0.804 | 0.716 | 0.908 |
| 18 | 0.836 | 0.775 | 0.887 | 0.834 | 0.773 | 0.891 | 0.795 | 0.689 | 0.871 | 0.799 | 0.711 | 0.883 | 0.807 | 0.703 | 0.883 |
| 19 | 0.844 | 0.783 | 0.884 | 0.831 | 0.763 | 0.883 | 0.799 | 0.709 | 0.873 | 0.803 | 0.707 | 0.884 | 0.775 | 0.737 | 0.882 |
| 20 | 0.848 | 0.796 | 0.888 | 0.831 | 0.757 | 0.887 | 0.794 | 0.708 | 0.868 | 0.786 | 0.689 | 0.880 | 0.851 | 0.740 | 0.886 |
| 21 | 0.841 | 0.790 | 0.878 | 0.827 | 0.737 | 0.894 | 0.788 | 0.701 | 0.871 | 0.785 | 0.712 | 0.856 | 0.829 | 0.731 | 0.894 |
| 22 | 0.840 | 0.760 | 0.896 | 0.830 | 0.741 | 0.884 | 0.798 | 0.710 | 0.877 | 0.808 | 0.726 | 0.887 | 0.866 | 0.723 | 0.894 |
| 23 | 0.845 | 0.781 | 0.891 | 0.828 | 0.756 | 0.882 | 0.812 | 0.706 | 0.878 | 0.792 | 0.696 | 0.874 | 0.769 | 0.741 | 0.907 |
| 24 | 0.846 | 0.791 | 0.888 | 0.835 | 0.763 | 0.886 | 0.803 | 0.718 | 0.870 | 0.796 | 0.706 | 0.894 | 0.798 | 0.733 | 0.870 |
| 25 | 0.842 | 0.787 | 0.885 | 0.833 | 0.753 | 0.885 | 0.811 | 0.710 | 0.888 | 0.808 | 0.715 | 0.891 | 0.823 | 0.735 | 0.902 |
| 26 | 0.840 | 0.763 | 0.883 | 0.827 | 0.755 | 0.885 | 0.789 | 0.688 | 0.861 | 0.796 | 0.722 | 0.878 | 0.860 | 0.741 | 0.890 |
| 27 | 0.844 | 0.775 | 0.890 | 0.834 | 0.744 | 0.881 | 0.799 | 0.705 | 0.864 | 0.795 | 0.709 | 0.879 | 0.773 | 0.705 | 0.886 |
| 28 | 0.839 | 0.773 | 0.878 | 0.823 | 0.763 | 0.881 | 0.800 | 0.716 | 0.886 | 0.789 | 0.703 | 0.876 | 0.798 | 0.745 | 0.895 |
| 29 | 0.846 | 0.800 | 0.880 | 0.831 | 0.762 | 0.883 | 0.794 | 0.700 | 0.857 | 0.792 | 0.693 | 0.865 | 0.835 | 0.734 | 0.880 |
| 30 | 0.835 | 0.762 | 0.882 | 0.819 | 0.746 | 0.880 | 0.798 | 0.699 | 0.874 | 0.788 | 0.697 | 0.869 | 0.758 | 0.695 | 0.888 |
| 31 | 0.845 | 0.778 | 0.884 | 0.822 | 0.730 | 0.883 | 0.794 | 0.714 | 0.876 | 0.801 | 0.697 | 0.877 | 0.864 | 0.706 | 0.883 |
| 32 | 0.853 | 0.798 | 0.894 | 0.829 | 0.736 | 0.885 | 0.800 | 0.689 | 0.874 | 0.793 | 0.703 | 0.868 | 0.835 | 0.713 | 0.901 |
| 33 | 0.843 | 0.773 | 0.889 | 0.837 | 0.774 | 0.888 | 0.804 | 0.720 | 0.888 | 0.797 | 0.701 | 0.882 | 0.849 | 0.734 | 0.882 |
| 34 | 0.843 | 0.780 | 0.876 | 0.827 | 0.755 | 0.879 | 0.795 | 0.696 | 0.866 | 0.798 | 0.700 | 0.878 | 0.804 | 0.698 | 0.891 |
| 35 | 0.845 | 0.773 | 0.889 | 0.836 | 0.732 | 0.893 | 0.798 | 0.699 | 0.878 | 0.801 | 0.702 | 0.872 | 0.800 | 0.739 | 0.878 |
| 36 | 0.837 | 0.777 | 0.887 | 0.832 | 0.751 | 0.891 | 0.795 | 0.671 | 0.887 | 0.794 | 0.692 | 0.864 | 0.794 | 0.752 | 0.873 |
| 37 | 0.839 | 0.775 | 0.877 | 0.820 | 0.723 | 0.894 | 0.788 | 0.675 | 0.873 | 0.790 | 0.706 | 0.879 | 0.820 | 0.740 | 0.888 |
| 38 | 0.845 | 0.773 | 0.893 | 0.828 | 0.738 | 0.884 | 0.790 | 0.708 | 0.874 | 0.792 | 0.697 | 0.874 | 0.800 | 0.755 | 0.888 |
| 39 | 0.841 | 0.775 | 0.891 | 0.828 | 0.751 | 0.889 | 0.798 | 0.684 | 0.878 | 0.801 | 0.705 | 0.874 | 0.798 | 0.727 | 0.904 |
| 40 | 0.849 | 0.762 | 0.891 | 0.827 | 0.755 | 0.886 | 0.791 | 0.711 | 0.886 | 0.794 | 0.706 | 0.885 | 0.791 | 0.733 | 0.899 |
| **Total mean** and 95% CI | **0.843** | 0.776 | 0.886 | **0.829** | 0.749 | 0.886 | **0.798** | 0.703 | 0.875 | **0.796** | 0.705 | 0.877 | **0.818** | 0.729 | 0.890 |
| 10-year specificity | | | | | | | | | | | | | | | |
|  | CoxSf | | | CoxEn | | | Feedforward | | | Densenet | | | Tab transformer | | |
| imp | mean | 95% lci | 95% uci | mean | 95% lci | 95% uci | mean | 95% lci | 95% uci | mean | 95% lci | 95% uci | mean | 95% lci | 95% uci |
| 1 | 0.850 | 0.750 | 0.910 | 0.845 | 0.766 | 0.905 | 0.811 | 0.735 | 0.885 | 0.811 | 0.726 | 0.879 | 0.826 | 0.733 | 0.891 |
| 2 | 0.856 | 0.754 | 0.904 | 0.834 | 0.746 | 0.893 | 0.799 | 0.702 | 0.879 | 0.817 | 0.728 | 0.882 | 0.815 | 0.743 | 0.863 |
| 3 | 0.847 | 0.759 | 0.914 | 0.836 | 0.742 | 0.903 | 0.807 | 0.728 | 0.878 | 0.809 | 0.707 | 0.874 | 0.823 | 0.769 | 0.882 |
| 4 | 0.856 | 0.757 | 0.912 | 0.842 | 0.754 | 0.903 | 0.814 | 0.723 | 0.886 | 0.818 | 0.736 | 0.893 | 0.831 | 0.776 | 0.884 |
| 5 | 0.835 | 0.746 | 0.903 | 0.835 | 0.752 | 0.896 | 0.808 | 0.707 | 0.893 | 0.807 | 0.720 | 0.898 | 0.816 | 0.732 | 0.881 |
| 6 | 0.838 | 0.734 | 0.904 | 0.835 | 0.752 | 0.901 | 0.806 | 0.713 | 0.876 | 0.817 | 0.732 | 0.894 | 0.817 | 0.785 | 0.902 |
| 7 | 0.852 | 0.755 | 0.918 | 0.854 | 0.792 | 0.906 | 0.817 | 0.725 | 0.884 | 0.812 | 0.726 | 0.887 | 0.839 | 0.750 | 0.902 |
| 8 | 0.838 | 0.755 | 0.907 | 0.835 | 0.747 | 0.904 | 0.801 | 0.700 | 0.872 | 0.801 | 0.708 | 0.880 | 0.808 | 0.776 | 0.880 |
| 9 | 0.854 | 0.757 | 0.909 | 0.838 | 0.737 | 0.915 | 0.805 | 0.698 | 0.888 | 0.811 | 0.729 | 0.878 | 0.835 | 0.792 | 0.907 |
| 10 | 0.847 | 0.753 | 0.905 | 0.842 | 0.733 | 0.907 | 0.815 | 0.727 | 0.881 | 0.806 | 0.726 | 0.879 | 0.821 | 0.757 | 0.889 |
| 11 | 0.855 | 0.770 | 0.906 | 0.837 | 0.764 | 0.895 | 0.805 | 0.709 | 0.879 | 0.808 | 0.705 | 0.877 | 0.824 | 0.732 | 0.882 |
| 12 | 0.837 | 0.758 | 0.899 | 0.838 | 0.735 | 0.906 | 0.805 | 0.709 | 0.879 | 0.806 | 0.730 | 0.863 | 0.823 | 0.741 | 0.899 |
| 13 | 0.847 | 0.740 | 0.913 | 0.839 | 0.751 | 0.896 | 0.811 | 0.728 | 0.877 | 0.810 | 0.722 | 0.886 | 0.833 | 0.775 | 0.913 |
| 14 | 0.851 | 0.769 | 0.908 | 0.846 | 0.762 | 0.905 | 0.809 | 0.718 | 0.887 | 0.805 | 0.716 | 0.863 | 0.828 | 0.772 | 0.893 |
| 15 | 0.853 | 0.764 | 0.912 | 0.843 | 0.764 | 0.906 | 0.807 | 0.704 | 0.879 | 0.815 | 0.731 | 0.888 | 0.824 | 0.723 | 0.898 |
| 16 | 0.840 | 0.733 | 0.916 | 0.834 | 0.747 | 0.914 | 0.802 | 0.695 | 0.867 | 0.811 | 0.713 | 0.887 | 0.806 | 0.741 | 0.844 |
| 17 | 0.852 | 0.780 | 0.905 | 0.836 | 0.760 | 0.906 | 0.806 | 0.710 | 0.901 | 0.811 | 0.728 | 0.877 | 0.787 | 0.743 | 0.905 |
| 18 | 0.841 | 0.742 | 0.898 | 0.837 | 0.739 | 0.909 | 0.804 | 0.705 | 0.881 | 0.816 | 0.737 | 0.896 | 0.829 | 0.720 | 0.896 |
| 19 | 0.860 | 0.793 | 0.910 | 0.845 | 0.773 | 0.902 | 0.814 | 0.726 | 0.883 | 0.816 | 0.742 | 0.899 | 0.803 | 0.774 | 0.871 |
| 20 | 0.856 | 0.764 | 0.910 | 0.844 | 0.765 | 0.892 | 0.803 | 0.709 | 0.880 | 0.802 | 0.710 | 0.892 | 0.825 | 0.782 | 0.909 |
| 21 | 0.857 | 0.733 | 0.913 | 0.832 | 0.732 | 0.894 | 0.812 | 0.730 | 0.892 | 0.810 | 0.722 | 0.881 | 0.822 | 0.760 | 0.901 |
| 22 | 0.835 | 0.748 | 0.908 | 0.833 | 0.749 | 0.905 | 0.810 | 0.716 | 0.891 | 0.823 | 0.747 | 0.885 | 0.827 | 0.765 | 0.902 |
| 23 | 0.853 | 0.769 | 0.908 | 0.839 | 0.761 | 0.910 | 0.808 | 0.725 | 0.875 | 0.813 | 0.716 | 0.876 | 0.806 | 0.774 | 0.865 |
| 24 | 0.850 | 0.743 | 0.911 | 0.842 | 0.748 | 0.905 | 0.805 | 0.727 | 0.876 | 0.810 | 0.733 | 0.876 | 0.830 | 0.734 | 0.891 |
| 25 | 0.841 | 0.747 | 0.906 | 0.836 | 0.754 | 0.904 | 0.813 | 0.725 | 0.887 | 0.802 | 0.707 | 0.878 | 0.829 | 0.732 | 0.874 |
| 26 | 0.840 | 0.750 | 0.898 | 0.836 | 0.751 | 0.911 | 0.807 | 0.714 | 0.867 | 0.811 | 0.736 | 0.881 | 0.804 | 0.769 | 0.914 |
| 27 | 0.849 | 0.759 | 0.912 | 0.859 | 0.791 | 0.910 | 0.808 | 0.718 | 0.877 | 0.820 | 0.745 | 0.879 | 0.820 | 0.761 | 0.861 |
| 28 | 0.849 | 0.740 | 0.908 | 0.836 | 0.757 | 0.903 | 0.801 | 0.715 | 0.878 | 0.810 | 0.722 | 0.882 | 0.824 | 0.727 | 0.883 |
| 29 | 0.848 | 0.738 | 0.906 | 0.847 | 0.757 | 0.904 | 0.813 | 0.727 | 0.881 | 0.805 | 0.715 | 0.879 | 0.847 | 0.752 | 0.895 |
| 30 | 0.836 | 0.758 | 0.902 | 0.836 | 0.752 | 0.899 | 0.804 | 0.704 | 0.873 | 0.802 | 0.704 | 0.887 | 0.795 | 0.743 | 0.897 |
| 31 | 0.848 | 0.751 | 0.913 | 0.836 | 0.737 | 0.898 | 0.811 | 0.705 | 0.906 | 0.814 | 0.719 | 0.889 | 0.821 | 0.745 | 0.909 |
| 32 | 0.857 | 0.761 | 0.917 | 0.837 | 0.752 | 0.894 | 0.808 | 0.701 | 0.883 | 0.810 | 0.718 | 0.883 | 0.843 | 0.734 | 0.898 |
| 33 | 0.851 | 0.755 | 0.907 | 0.838 | 0.761 | 0.902 | 0.807 | 0.728 | 0.874 | 0.805 | 0.717 | 0.865 | 0.840 | 0.733 | 0.891 |
| 34 | 0.844 | 0.764 | 0.899 | 0.843 | 0.770 | 0.900 | 0.809 | 0.724 | 0.890 | 0.810 | 0.728 | 0.886 | 0.828 | 0.720 | 0.863 |
| 35 | 0.864 | 0.790 | 0.913 | 0.844 | 0.727 | 0.907 | 0.811 | 0.711 | 0.879 | 0.820 | 0.717 | 0.895 | 0.833 | 0.751 | 0.870 |
| 36 | 0.841 | 0.742 | 0.906 | 0.850 | 0.768 | 0.909 | 0.808 | 0.727 | 0.884 | 0.805 | 0.711 | 0.884 | 0.841 | 0.784 | 0.865 |
| 37 | 0.837 | 0.750 | 0.907 | 0.835 | 0.761 | 0.906 | 0.803 | 0.709 | 0.886 | 0.810 | 0.714 | 0.895 | 0.827 | 0.773 | 0.878 |
| 38 | 0.845 | 0.758 | 0.916 | 0.844 | 0.766 | 0.900 | 0.809 | 0.724 | 0.888 | 0.815 | 0.745 | 0.878 | 0.829 | 0.774 | 0.862 |
| 39 | 0.846 | 0.753 | 0.915 | 0.845 | 0.768 | 0.896 | 0.807 | 0.714 | 0.872 | 0.810 | 0.731 | 0.869 | 0.818 | 0.755 | 0.871 |
| 40 | 0.838 | 0.736 | 0.910 | 0.834 | 0.751 | 0.893 | 0.804 | 0.722 | 0.886 | 0.805 | 0.704 | 0.884 | 0.810 | 0.762 | 0.879 |
| **Total mean** and 95% CI | **0.847** | 0.754 | 0.909 | **0.840** | 0.755 | 0.903 | **0.808** | 0.716 | 0.882 | **0.811** | 0.723 | 0.883 | **0.823** | 0.754 | 0.887 |
| 12-year specificity | | | | | | | | | | | | | | | |
|  | CoxSf | | | CoxEn | | | Feedforward | | | Densenet | | | Tab transformer | | |
| imp | mean | 95% lci | 95% uci | mean | 95% lci | 95% uci | mean | 95% lci | 95% uci | mean | 95% lci | 95% uci | mean | 95% lci | 95% uci |
| 1 | 0.799 | 0.727 | 0.892 | 0.810 | 0.689 | 0.911 | 0.780 | 0.667 | 0.891 | 0.786 | 0.675 | 0.873 | 0.782 | 0.678 | 0.851 |
| 2 | 0.792 | 0.723 | 0.882 | 0.792 | 0.688 | 0.891 | 0.771 | 0.643 | 0.897 | 0.778 | 0.660 | 0.897 | 0.783 | 0.669 | 0.896 |
| 3 | 0.800 | 0.732 | 0.887 | 0.819 | 0.686 | 0.911 | 0.785 | 0.651 | 0.884 | 0.783 | 0.661 | 0.880 | 0.827 | 0.754 | 0.894 |
| 4 | 0.804 | 0.728 | 0.894 | 0.812 | 0.687 | 0.898 | 0.786 | 0.629 | 0.895 | 0.781 | 0.676 | 0.878 | 0.809 | 0.699 | 0.913 |
| 5 | 0.799 | 0.728 | 0.880 | 0.813 | 0.711 | 0.900 | 0.766 | 0.630 | 0.886 | 0.781 | 0.675 | 0.887 | 0.805 | 0.670 | 0.917 |
| 6 | 0.787 | 0.727 | 0.877 | 0.796 | 0.649 | 0.896 | 0.775 | 0.619 | 0.889 | 0.776 | 0.670 | 0.869 | 0.828 | 0.753 | 0.861 |
| 7 | 0.802 | 0.731 | 0.899 | 0.811 | 0.684 | 0.916 | 0.781 | 0.653 | 0.897 | 0.780 | 0.657 | 0.886 | 0.839 | 0.683 | 0.902 |
| 8 | 0.797 | 0.709 | 0.888 | 0.796 | 0.691 | 0.888 | 0.780 | 0.659 | 0.893 | 0.773 | 0.679 | 0.880 | 0.816 | 0.706 | 0.889 |
| 9 | 0.804 | 0.717 | 0.910 | 0.813 | 0.671 | 0.900 | 0.776 | 0.649 | 0.880 | 0.775 | 0.661 | 0.867 | 0.836 | 0.696 | 0.897 |
| 10 | 0.798 | 0.718 | 0.880 | 0.805 | 0.690 | 0.886 | 0.794 | 0.695 | 0.894 | 0.774 | 0.663 | 0.865 | 0.793 | 0.729 | 0.897 |
| 11 | 0.792 | 0.723 | 0.880 | 0.794 | 0.670 | 0.890 | 0.778 | 0.649 | 0.894 | 0.772 | 0.652 | 0.863 | 0.827 | 0.733 | 0.867 |
| 12 | 0.795 | 0.724 | 0.872 | 0.801 | 0.669 | 0.914 | 0.779 | 0.657 | 0.887 | 0.775 | 0.658 | 0.866 | 0.812 | 0.744 | 0.861 |
| 13 | 0.788 | 0.715 | 0.899 | 0.805 | 0.693 | 0.906 | 0.790 | 0.649 | 0.909 | 0.781 | 0.653 | 0.883 | 0.825 | 0.733 | 0.876 |
| 14 | 0.793 | 0.731 | 0.879 | 0.812 | 0.691 | 0.896 | 0.788 | 0.673 | 0.900 | 0.775 | 0.660 | 0.880 | 0.819 | 0.648 | 0.897 |
| 15 | 0.799 | 0.729 | 0.892 | 0.803 | 0.642 | 0.892 | 0.773 | 0.657 | 0.882 | 0.793 | 0.683 | 0.899 | 0.833 | 0.695 | 0.891 |
| 16 | 0.797 | 0.739 | 0.891 | 0.805 | 0.655 | 0.913 | 0.776 | 0.637 | 0.877 | 0.789 | 0.685 | 0.890 | 0.797 | 0.703 | 0.863 |
| 17 | 0.808 | 0.728 | 0.899 | 0.810 | 0.695 | 0.910 | 0.786 | 0.655 | 0.886 | 0.778 | 0.659 | 0.871 | 0.779 | 0.714 | 0.879 |
| 18 | 0.793 | 0.720 | 0.880 | 0.803 | 0.672 | 0.910 | 0.790 | 0.670 | 0.884 | 0.785 | 0.674 | 0.894 | 0.795 | 0.723 | 0.909 |
| 19 | 0.797 | 0.708 | 0.893 | 0.811 | 0.680 | 0.893 | 0.781 | 0.656 | 0.890 | 0.775 | 0.666 | 0.861 | 0.813 | 0.753 | 0.900 |
| 20 | 0.796 | 0.734 | 0.886 | 0.809 | 0.692 | 0.909 | 0.775 | 0.668 | 0.891 | 0.772 | 0.633 | 0.883 | 0.828 | 0.652 | 0.903 |
| 21 | 0.793 | 0.716 | 0.898 | 0.811 | 0.686 | 0.893 | 0.798 | 0.669 | 0.900 | 0.786 | 0.692 | 0.872 | 0.824 | 0.667 | 0.909 |
| 22 | 0.795 | 0.716 | 0.883 | 0.805 | 0.645 | 0.878 | 0.772 | 0.660 | 0.883 | 0.780 | 0.650 | 0.888 | 0.807 | 0.746 | 0.841 |
| 23 | 0.806 | 0.733 | 0.902 | 0.803 | 0.682 | 0.894 | 0.785 | 0.661 | 0.903 | 0.782 | 0.657 | 0.898 | 0.816 | 0.664 | 0.883 |
| 24 | 0.786 | 0.723 | 0.878 | 0.799 | 0.670 | 0.907 | 0.782 | 0.646 | 0.896 | 0.778 | 0.660 | 0.868 | 0.786 | 0.674 | 0.892 |
| 25 | 0.810 | 0.732 | 0.891 | 0.804 | 0.663 | 0.897 | 0.766 | 0.658 | 0.869 | 0.784 | 0.664 | 0.887 | 0.784 | 0.714 | 0.929 |
| 26 | 0.798 | 0.732 | 0.860 | 0.808 | 0.675 | 0.897 | 0.787 | 0.663 | 0.901 | 0.788 | 0.672 | 0.875 | 0.789 | 0.672 | 0.933 |
| 27 | 0.802 | 0.730 | 0.893 | 0.815 | 0.711 | 0.907 | 0.775 | 0.639 | 0.882 | 0.788 | 0.671 | 0.891 | 0.835 | 0.722 | 0.870 |
| 28 | 0.789 | 0.717 | 0.890 | 0.805 | 0.703 | 0.901 | 0.780 | 0.650 | 0.895 | 0.771 | 0.659 | 0.869 | 0.797 | 0.742 | 0.899 |
| 29 | 0.799 | 0.719 | 0.900 | 0.804 | 0.685 | 0.921 | 0.785 | 0.662 | 0.885 | 0.777 | 0.691 | 0.865 | 0.794 | 0.718 | 0.842 |
| 30 | 0.803 | 0.737 | 0.888 | 0.815 | 0.714 | 0.906 | 0.765 | 0.605 | 0.862 | 0.775 | 0.663 | 0.895 | 0.797 | 0.746 | 0.865 |
| 31 | 0.796 | 0.723 | 0.891 | 0.818 | 0.700 | 0.902 | 0.785 | 0.648 | 0.900 | 0.779 | 0.639 | 0.872 | 0.799 | 0.730 | 0.867 |
| 32 | 0.787 | 0.719 | 0.908 | 0.798 | 0.673 | 0.904 | 0.784 | 0.664 | 0.898 | 0.772 | 0.653 | 0.881 | 0.819 | 0.704 | 0.909 |
| 33 | 0.799 | 0.728 | 0.868 | 0.804 | 0.672 | 0.885 | 0.773 | 0.646 | 0.892 | 0.776 | 0.653 | 0.880 | 0.831 | 0.761 | 0.876 |
| 34 | 0.810 | 0.739 | 0.892 | 0.820 | 0.702 | 0.909 | 0.774 | 0.680 | 0.893 | 0.775 | 0.679 | 0.876 | 0.843 | 0.739 | 0.878 |
| 35 | 0.797 | 0.739 | 0.885 | 0.820 | 0.706 | 0.898 | 0.788 | 0.677 | 0.898 | 0.795 | 0.668 | 0.888 | 0.807 | 0.726 | 0.879 |
| 36 | 0.803 | 0.720 | 0.903 | 0.803 | 0.673 | 0.909 | 0.766 | 0.646 | 0.884 | 0.778 | 0.662 | 0.866 | 0.825 | 0.704 | 0.894 |
| 37 | 0.793 | 0.723 | 0.885 | 0.796 | 0.660 | 0.897 | 0.793 | 0.683 | 0.894 | 0.788 | 0.676 | 0.899 | 0.813 | 0.746 | 0.868 |
| 38 | 0.801 | 0.749 | 0.885 | 0.819 | 0.664 | 0.896 | 0.788 | 0.674 | 0.898 | 0.791 | 0.692 | 0.888 | 0.819 | 0.721 | 0.880 |
| 39 | 0.804 | 0.740 | 0.887 | 0.826 | 0.738 | 0.902 | 0.786 | 0.671 | 0.903 | 0.794 | 0.687 | 0.889 | 0.819 | 0.742 | 0.898 |
| 40 | 0.795 | 0.718 | 0.862 | 0.803 | 0.693 | 0.906 | 0.779 | 0.638 | 0.887 | 0.775 | 0.675 | 0.867 | 0.832 | 0.758 | 0.879 |
| **Total mean** and 95% CI | **0.798** | 0.726 | 0.888 | **0.807** | 0.683 | 0.901 | **0.781** | 0.655 | 0.891 | **0.780** | 0.667 | 0.880 | **0.812** | 0.713 | 0.886 |

Abbreviations: CoxSf: Cox models with selected features; CoxEn: Cox models with Elastic Net regularization; FeedForward: Feedforward neural network; Densenet: Densely Connected Convolutional Network; TabTransformer: TabTransformer neural network; imp: imputation number; LCI: 95% lower confidence interval; UCI: 95% upper confidence interval; 95% CI: 95% confidence intervals

## Supplementary Figure 1. Flowchart of participant’s selection at baseline (2004-2005) and attrition from 2004-2005 to 2016-2017. The English Longitudinal Study of Ageing


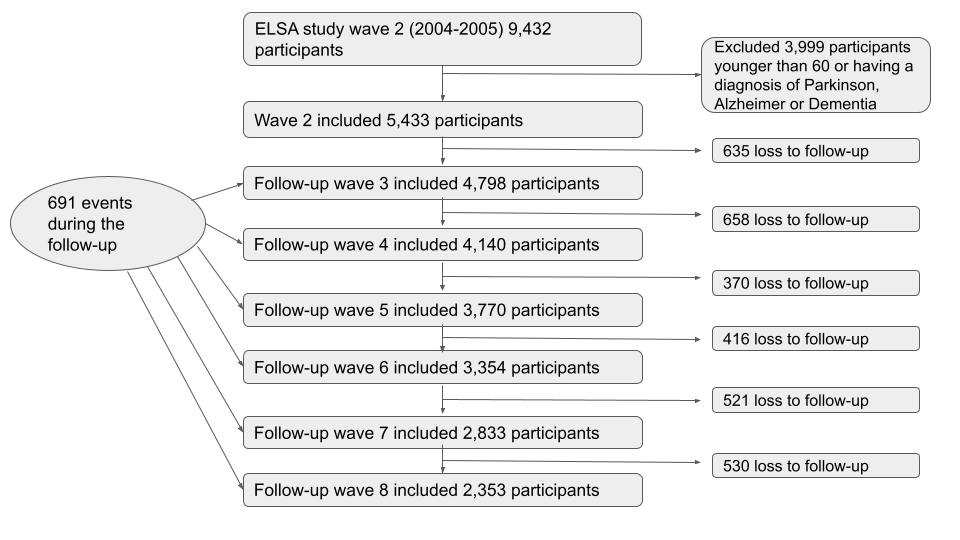


## Supplementary Figure 2. Observed and imputed data: Memory and Executive scores


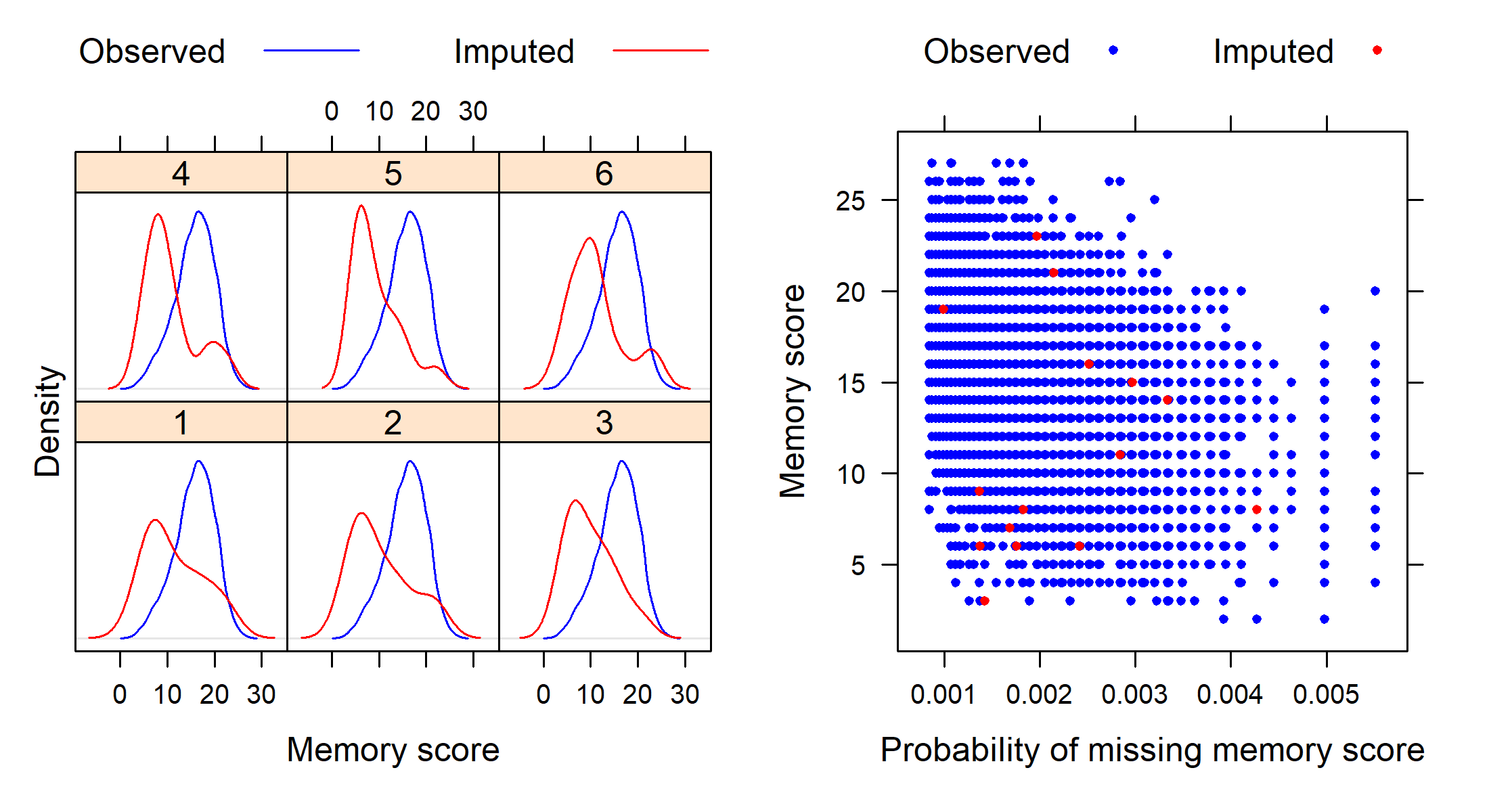


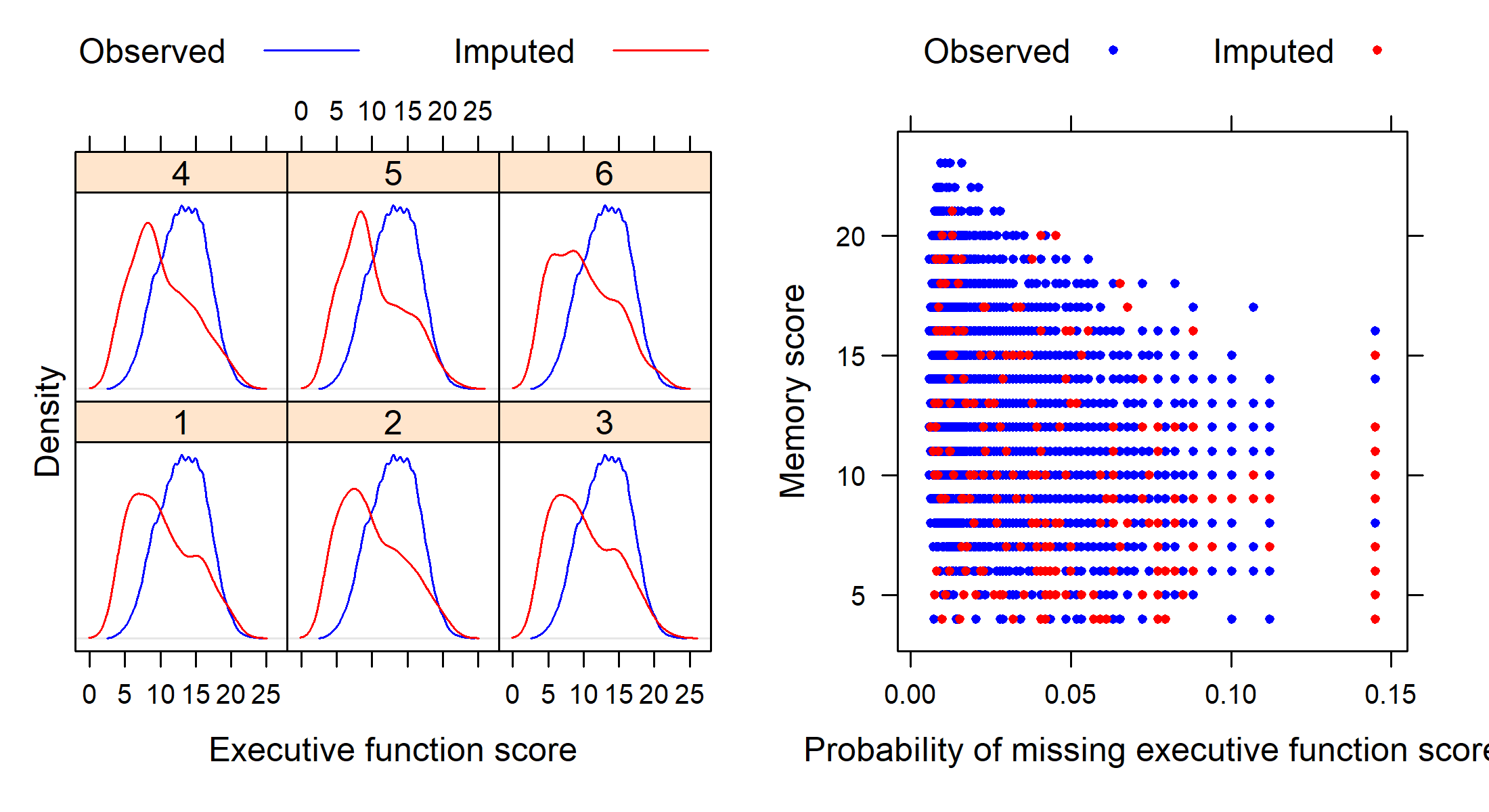


## Supplementary Figure 3. Observed and imputed data: Gait speed and BMI and Executive scores


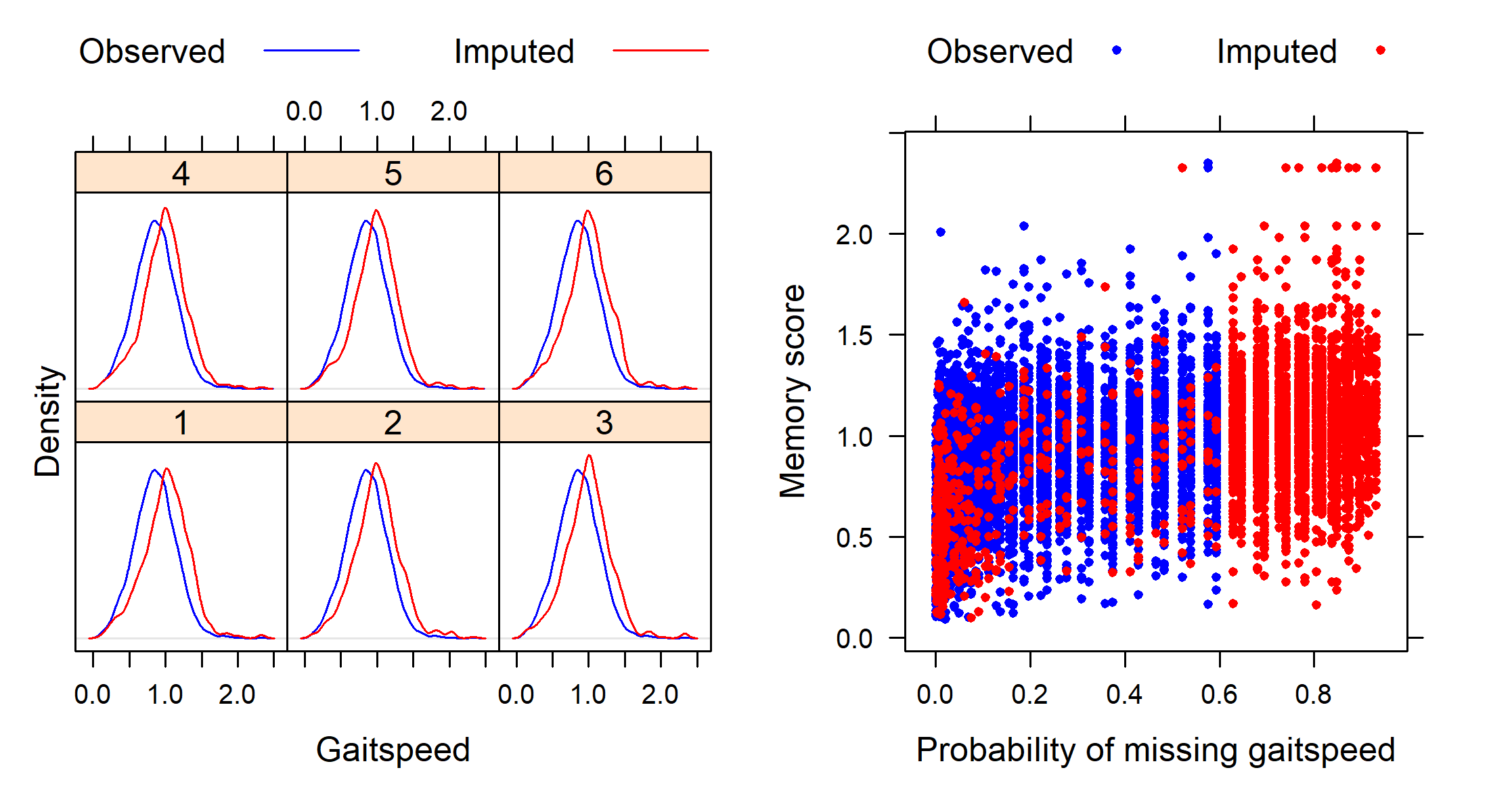


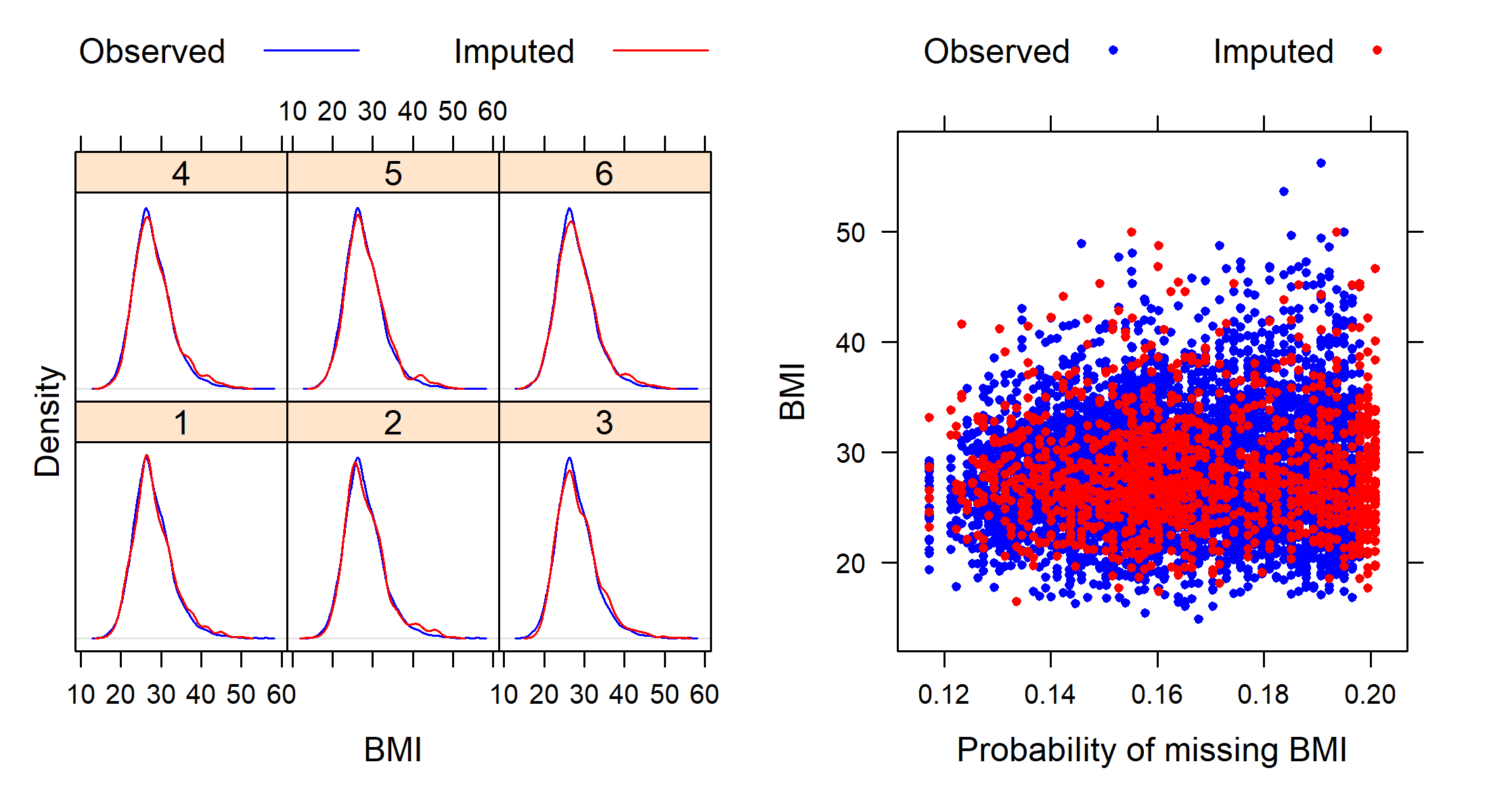


## Supplementary Figure 4. Observed and imputed data: Chair rise time and pulse rate


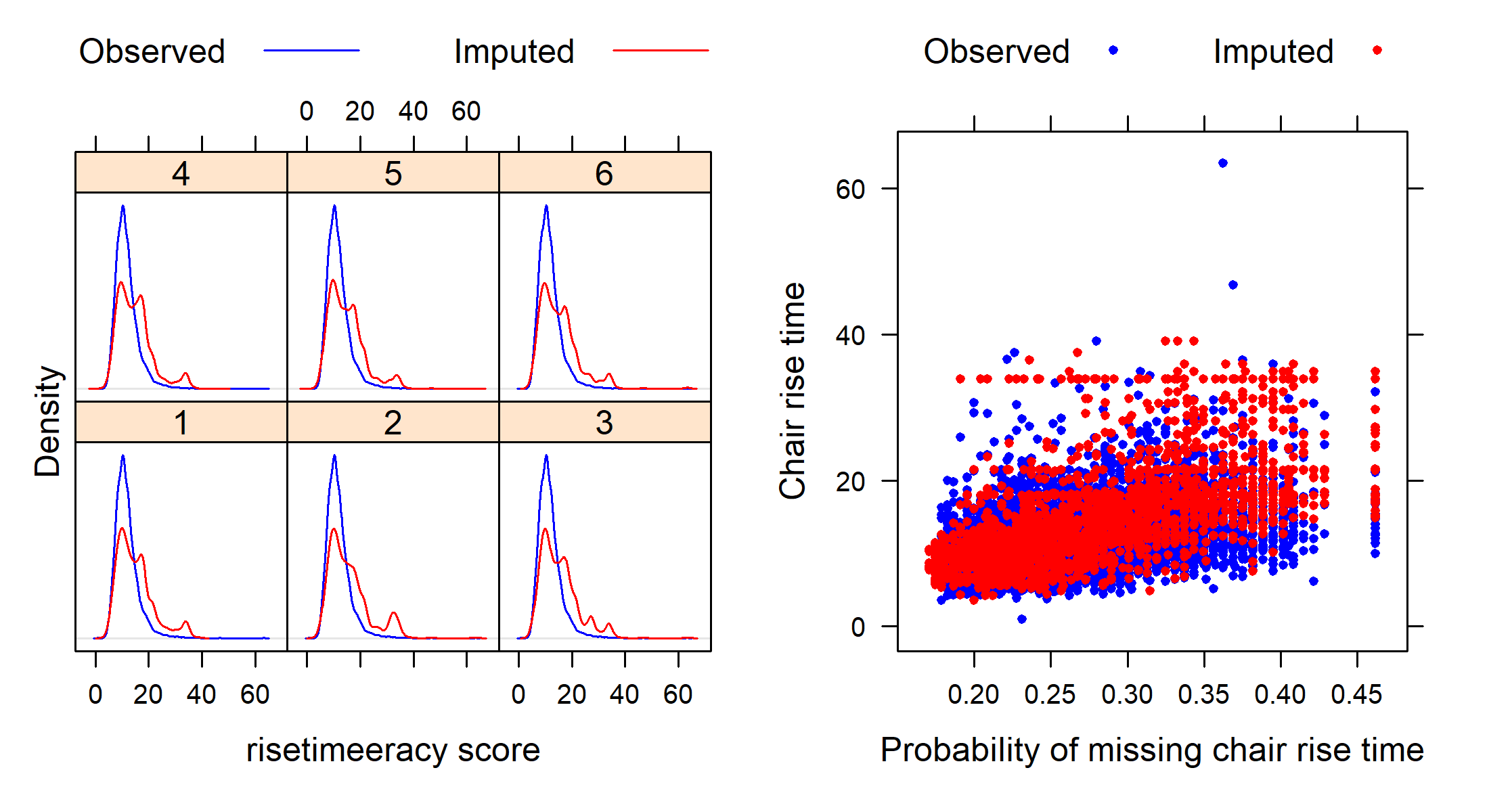


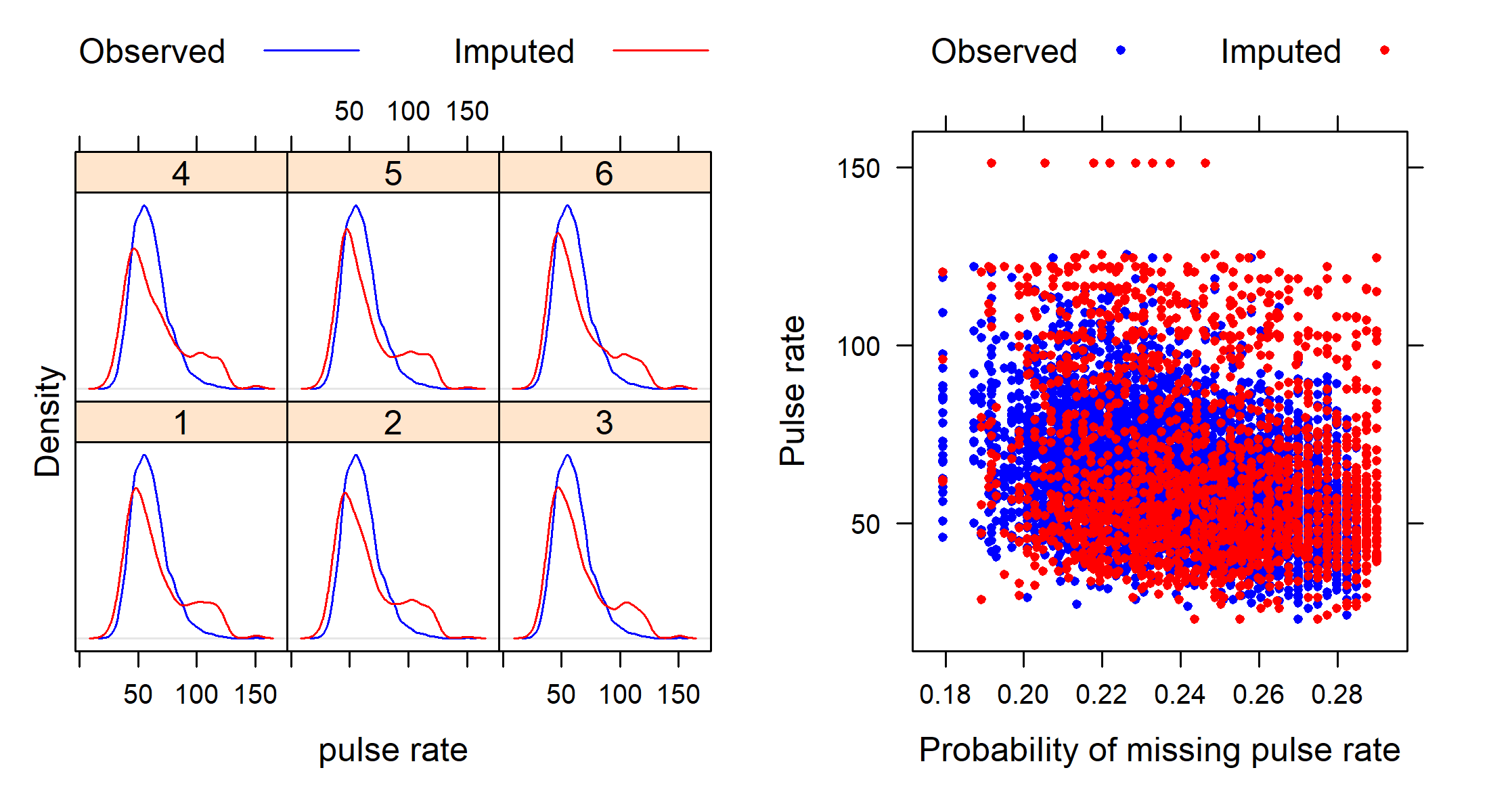


## Supplementary Figure 5. Evaluation of overfitting of machine learning models predicting new events of neurodegenerative diseases


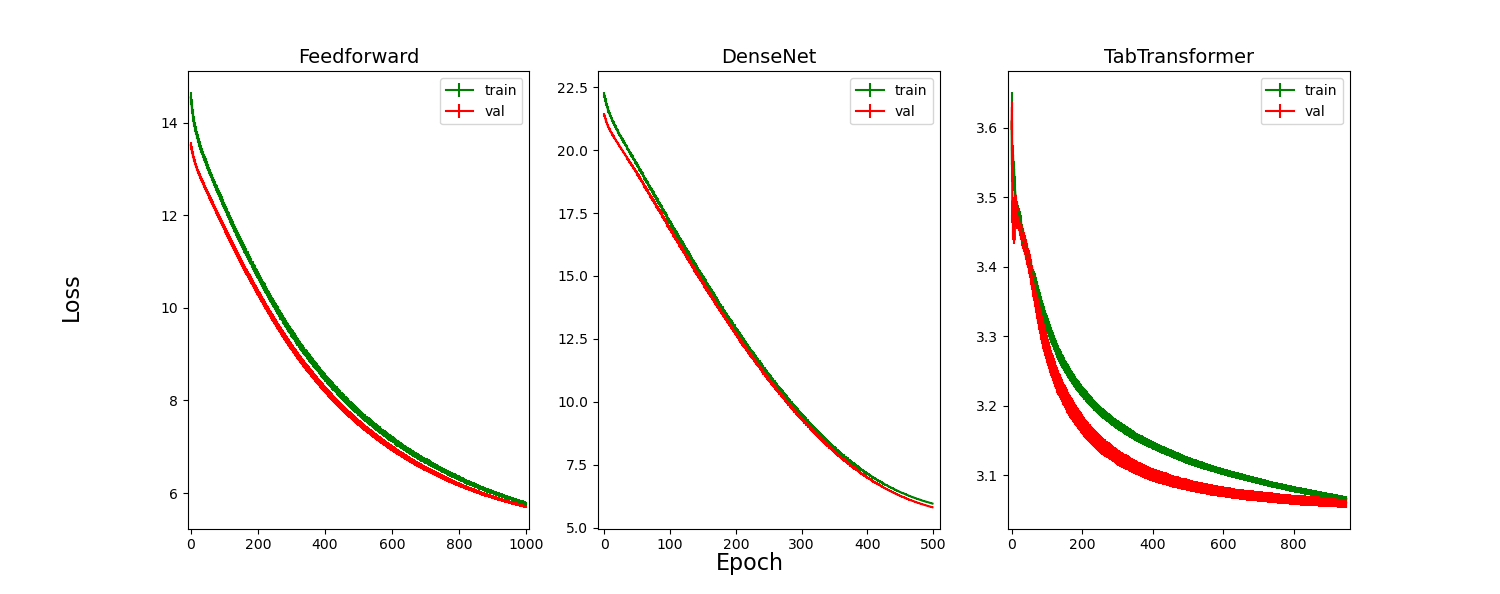


## Supplementary Figure 6. SHAP feature importance and summary plots in deep neural models

Feedforward Densenet TabTransformer


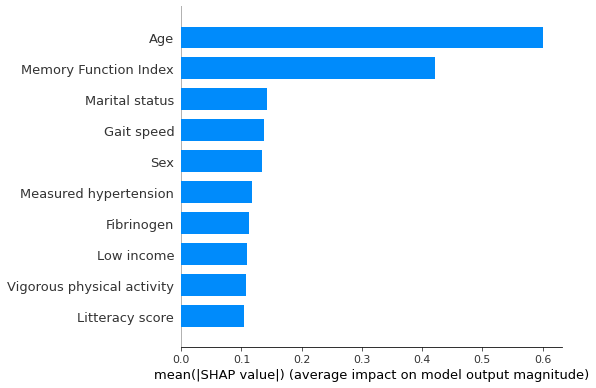

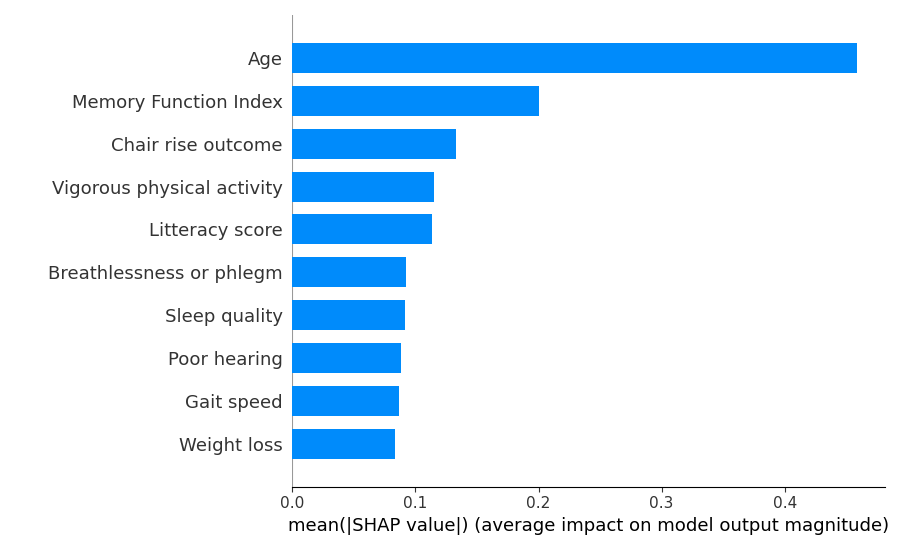

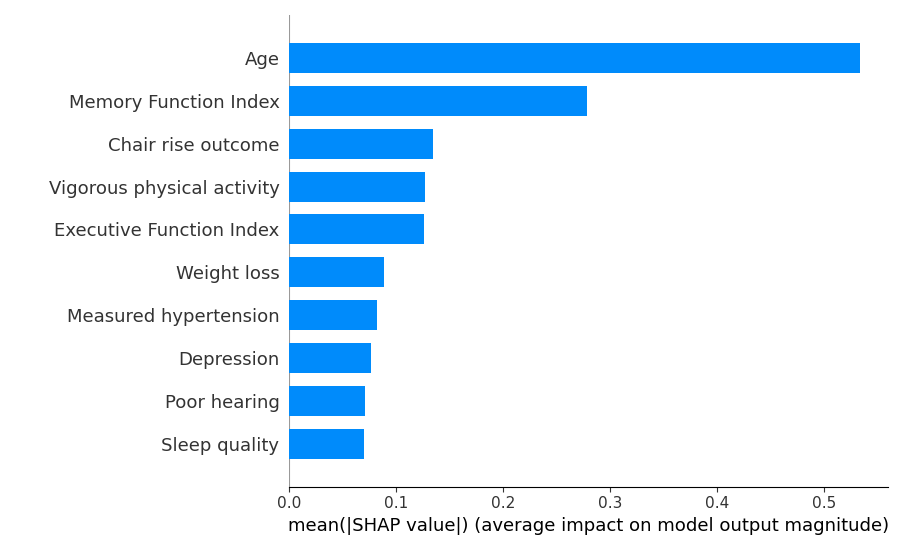


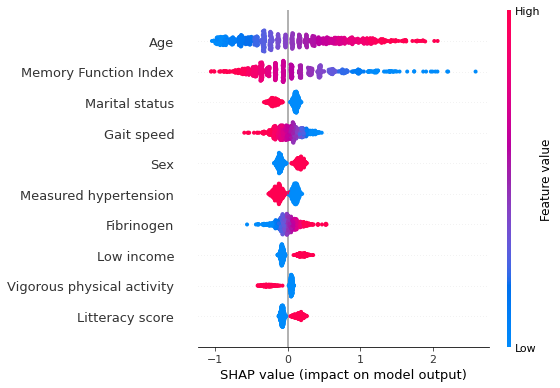


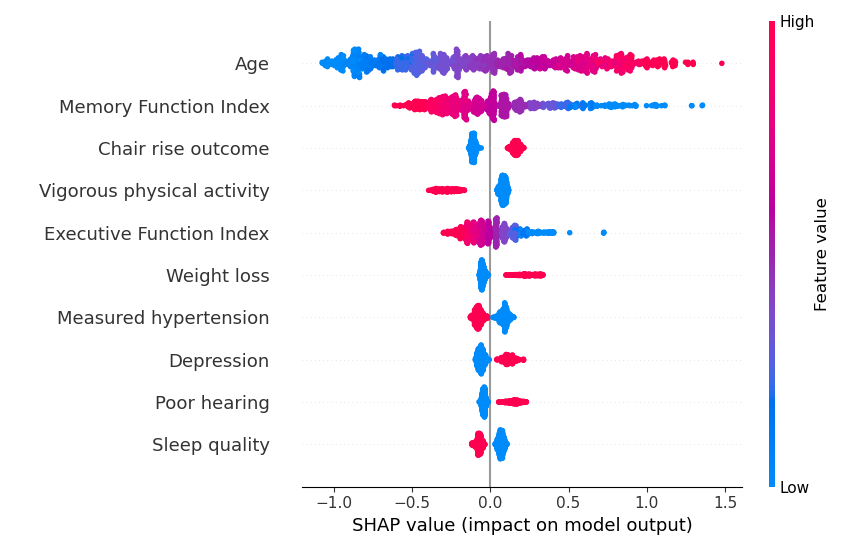

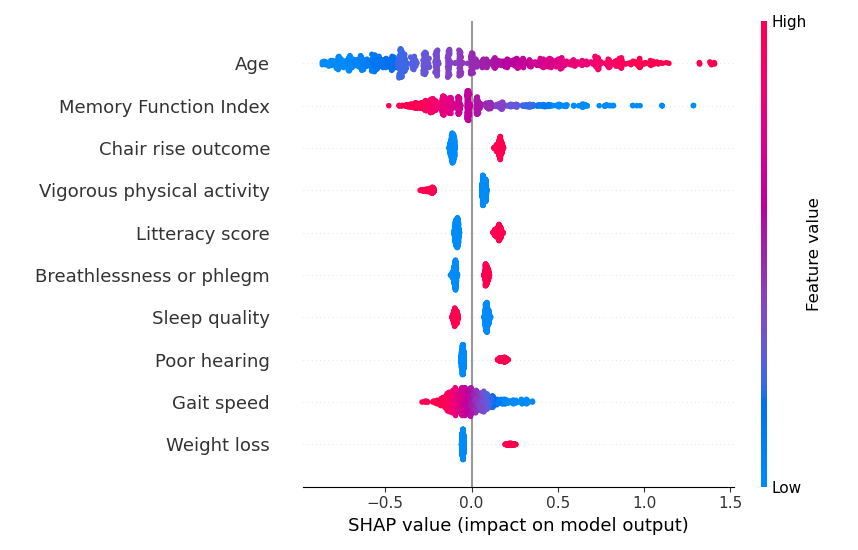


The first, second and third columns show the top ten feature importance of the Feedforward, the Densenet, and the TabTransformer neural networks respectively. The first row shows the feature importance as the mean absolute SHAP values, and the second row is a summary plot showing the impact of each feature value on the prediction and the direction of the association.

## Supplementary Figure 7. Intercept of variables among deep neural models and Cox models


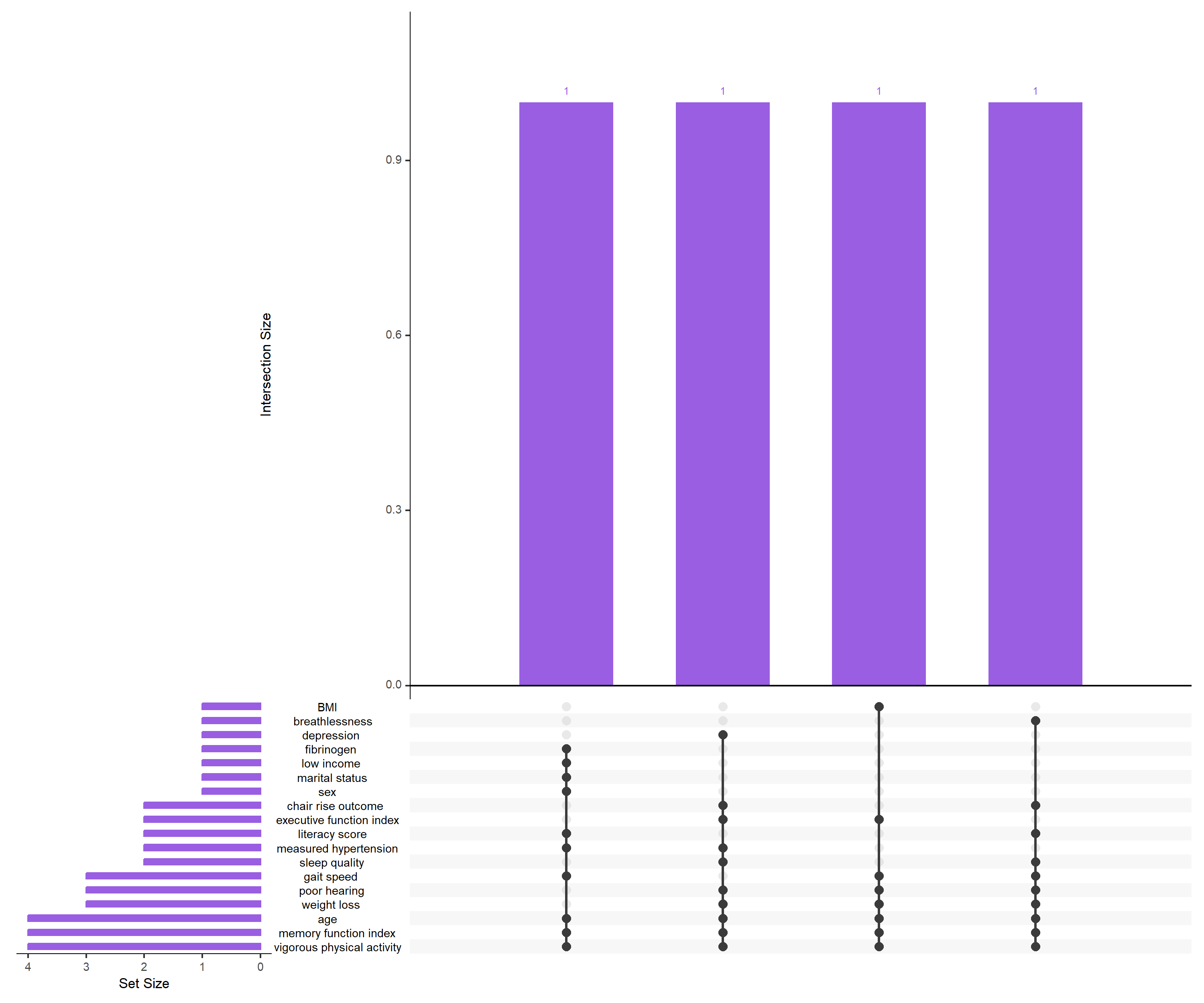

Supplement: Supplementary file 1 — Additional file 1: Supplementary Table 1. Features candidates to predictors to be included in the models. Supplementary Table 2. Comparison between imputed and non-imputed original values. Supplementary Table 3. Uno’s C Statistics mean and 95% confidence intervals. Supplementary Table 4. Time-dependent AUC mean and 95% confidence intervals. Supplementary Table 5. Time-dependent balanced accuracy mean and 95% confidence intervals. Supplementary Table 6. Time-dependent sensitivity mean and 95% confidence intervals. Supplementary Table 7. Time-dependent specificity mean and 95% confidence intervals. Supplementary Fig. 1. Flowchart of participant’s selection at baseline (2004–2005) and attrition from 2004 to 2005 to 2016–2017. The English Longitudinal Study of Ageing. Supplementary Fig. 2. Observed and imputed data: Memory and Executive scores. Supplementary Fig. 3. Observed and imputed data: Gait speed and BMI and Executive scores. Supplementary Fig. 4. Observed and imputed data: Chair rise time and pulse rate. Supplementary Fig. 5. Evaluation of overfitting of machine learning models predicting new events of neurodegenerative diseases. Supplementary Fig. 6. SHAP feature importance and summary plots in deep neural models. Supplementary Fig. 7. Intercept of variables among deep neural models and Cox models. [file 12874_2023_1837_MOESM1_ESM.docx]
